# Heterogeneous genetic diversity pattern in *Plasmodium vivax* genes encoding merozoite surface proteins (MSP) -7E, −7F and -7L

**DOI:** 10.1186/1475-2875-13-495

**Published:** 2014-12-13

**Authors:** Diego Garzón-Ospina, Johanna Forero-Rodríguez, Manuel A Patarroyo

**Affiliations:** Molecular Biology and Immunology Department, Fundación Instituto de Inmunología de Colombia (FIDIC), Carrera 50 No. 26-20, Bogotá, DC Colombia; Basic Sciences Department, School of Medicine and Health Sciences, Universidad del Rosario, Carrera 24 No. 63C-69, Bogotá, DC Colombia

**Keywords:** Plasmodium vivax, msp-7, Genetic diversity, Natural selection, Selective sweep

## Abstract

**Background:**

The *msp-7* gene has become differentially expanded in the *Plasmodium* genus; *Plasmodium vivax* has the highest copy number of this gene, several of which encode antigenic proteins in merozoites.

**Methods:**

DNA sequences from thirty-six Colombian clinical isolates from *P. vivax* (*pv*) *msp-7E*, −*7F* and *-7L* genes were analysed for characterizing and studying the genetic diversity of these *pvmsp-7* members which are expressed during the intra-erythrocyte stage; natural selection signals producing the variation pattern so observed were evaluated.

**Results:**

The *pvmsp-7E* gene was highly polymorphic compared to *pvmsp-7F* and *pvmsp-7L* which were seen to have limited genetic diversity; *pvmsp-7E* polymorphism was seen to have been maintained by different types of positive selection. Even though these copies seemed to be species-specific duplications, a search in the *Plasmodium cynomolgi* genome (*P. vivax* sister taxon) showed that both species shared the whole *msp-7* repertoire. This led to exploring the long-term effect of natural selection by comparing the orthologous sequences which led to finding signatures for lineage-specific positive selection.

**Conclusions:**

The results confirmed that the *P. vivax msp-7* family has a heterogeneous genetic diversity pattern; some members are highly conserved whilst others are highly diverse. The results suggested that the 3′-end of these genes encode MSP-7 proteins’ functional region whilst the central region of *pvmsp-7E* has evolved rapidly. The lineage-specific positive selection signals found suggested that mutations occurring in *msp-7s* genes during host switch may have succeeded in adapting the ancestral *P. vivax* parasite population to humans.

**Electronic supplementary material:**

The online version of this article (doi:10.1186/1475-2875-13-495) contains supplementary material, which is available to authorized users.

## Background

Malaria remains a major public health problem worldwide. *Plasmodium falciparum* is the parasite species causing the lethal form of the disease whilst *Plasmodium vivax* has long been considered a parasite causing mild disease, thereby diverting attention away from this species regarding research; however, recent studies have reported that this species also causes severe clinical syndromes [1, 2]. Even though both species infect humans, they both emerged from different evolutionary lineages; whilst *P. vivax* shares a common ancestor with Asian non-human primate malaria, *P. falciparum* has diverged from parasites infecting great apes [3].

The different evolutionary paths leading to the appearance of *P. vivax* and *P. falciparum* have also led to important differences regarding hosts being invaded by both species [4, 5]. In spite of such differences, initial interaction between the parasite and red blood cells (RBC) seems to be directed by the MSP-1 protein [6–8] which is present in all species from the genus. MSP-1 forms a complex with MSP-6 and MSP-7 in *P. falciparum* [9–11]; the latter protein is encoded by a gene forming part of a multigene family which has been differentially expanded amongst *Plasmodium* species [12]. Studies involving *msp-7* family members have shown that the resulting protein products are located on the parasite membrane and that a 22 kDa C-terminal fragment (derived from proteolytic processing during parasite development) [10] has regions interacting with RBC [13]. The *msp-7* knockout in *P. falciparum* (*pfmsp-7I*) and *Plasmodium berghei* (*pbmsp-7B*) has shown that even though its absence is not lethal, it does reduce mutant parasite invasion ability [14, 15]. These results, together with prior *in silico* analysis, have suggested that the members of this family could have functional redundancy [12, 15, 16] and their protein products (or some of them) could thus be involved in invasion. On the other hand, antigenicity studies have shown that some of these genes’ protein products are recognized by sera from infected patients [17, 18]. Antibodies directed against these proteins can inhibit parasite invasion of RBC [19], whilst immunization with members of the *Plasmodium yoelii msp-7* family has shown that they can confer protection in vaccinated mice following experimental challenge [20].

The genetic variability patterns observed in *msp-7* family members have been different between *P. falciparum* and *P. vivax* [21–24]; whilst members of the former species have low polymorphism [23, 24], some members of *P. vivax* (*pvmsp-7C*, *pvmsp-7H* and *pvmsp-7I*) are highly polymorphic [21]. However, other members, such as *pvmsp-7A* and *pvmsp-7K*, are amongst the most conserved *P. vivax* antigens [22]. There are thirteen *msp-7* genes in this species’ chromosome 12; these have been named in alphabetical order according to their location regarding the PVX_082640 gene [12]. Eleven of these genes are transcribed, but only seven of them are transcribed during the last hours of the intra-erythrocyte stage [25]. The genetic diversity of four of these seven genes has already been evaluated [21, 22]; this study was therefore aimed at evaluating the genetic variability of the three remaining members (*pvmsp-7E*, *pvmsp-7F* and *pvmsp-7L*) which are expressed during the intra-erythrocyte stage. *pvmsp-7E* displayed high polymorphism and its central region had undergone rapid evolution whilst *pvmsp-7F* and *pvmsp-7L* were seen to be highly conserved. The genes’ 3′-ends tended to be conserved by negative selection, suggesting that they encode the functional region for these proteins. Similar to what happened with the *msp-1* gene [26, 27], *msp-7* genes seem to have diverged due to positive selection, which could have resulted from malaria parasites adaptation to different hosts.

## Methods

### Ethics statement

All *P. vivax*-infected patients who provided us with the blood samples were informed about the purpose of the study and all gave their written consent. All procedures carried out in this study were approved by the ethics committee of the Fundación Instituto de Inmunología de Colombia.

### Parasite DNA and genotyping

Thirty-six peripheral blood samples from patients proving positive for *P. vivax* malaria by microscope examination were collected from some of Colombia’s departments (Chocó and Nariño in the south-west, Guainía, Guaviare and Meta in the south-east, Tolima in the Midwest, and Atlántico, Antioquia and Córdoba in the north-west) between 2007 and 2010 (nine isolates in 2007, seven isolates in 2008, 8 isolates in 2009 and twelve isolates in 2010). DNA was obtained using a Wizard Genomic DNA Purification kit (Promega), following the manufacturer’s instructions, and stored at −20°C until use. The parasite samples were genotyped by PCR-RFLP of the *pvmsp-1* gene as previously described [28]. Samples having single *P. vivax msp-1* allele infection were used for PCR amplification.

### PCR amplification and sequencing

Primers were designed for amplifying *pvmsp-7E*, *pvmsp-7F* and *pvmsp-7L* DNA fragments, based on Sal-I sequences (PlasmoDB IDs: PVX_082665, PVX_082670 and PVX_082700, respectively). The *pvmsp-7E* gene fragment was amplified with 7Edto 5′ GCCGATCTGTTGTCTTTTCC 3′ and 7Erev 5′ CCTTACGACACGTCAAATGG 3′ primers*. pvmsp-7F* was amplified by using 7Fdto 5′ TCCTCTCCTTGCTGATACTCC 3′ and 7Frev 5′ CAGCCGCTTAAATCACTTC 3′ primers whilst *pvmsp-7L* was amplified with 7Ldto 5′ AGTACTATTCTTCTTGCCGTCC 3′ and 7Lrev 5′ TCCCCTCAGTAGTAAAACATCG 3′ primers. All PCR reactions were performed using KAPA HiFi HotStart Readymix containing 0.3 μM of each primer in a final 25 μL volume. Thermal conditions were set as follows: one cycle of 5 min at 95°C, 30 cycles of 20 sec at 98°C, 15 sec at 63°C, 30 sec at 72°C, followed by a 5 min final extension at 72°C. PCR products were purified using the UltraClean PCR Clean-up (MO BIO) kit, and then sequenced with a BigDye Terminator kit (Macrogen, Seoul, South Korea) in both directions. Three PCR products obtained from independent PCR amplifications were sequenced per isolate to discard errors. Sequences having a different haplotype to the previously reported ones were deposited in the GenBank database (accession numbers KM212276 - KM212302).

### Phylogenetic analysis for *Plasmodium cynomolgi msp-7*orthologous identification

A similar approach used for *msp-7* identification in other *Plasmodium* species [12] was adopted for identifying *msp-7* genes in *Plasmodium cynomolgi* (*pc*) and establishing their orthologous relationships. The genomic region (obtained from GenBank, accession number: NC_020405) encoded by the PCYB_122860 and PCYB_122720 genes (homologues to PVX_082640 and PVX_082715 which circumscribed the *msp-7* region in *P. vivax*) was analysed using ORF Finder [29] and Gene Runner software for identifying open reading frames (ORFs) encoding proteins larger than 300 amino acids. Deduced amino acid sequences obtained with Gene Runner were aligned with *P. vivax* (12 proteins) and *Plasmodium knowlesi* (5 proteins) MSP-7 sequences using the MUSCLE algorithm [30]. The best model for amino acid substitutions was selected by Akaike’s information criterion using the ProtTest program [31]. Phylogenetic trees were inferred through Maximum Likelihood (ML) and Bayesian (BY) methods using the JTT+G model. The observed amino acid frequencies (JTT+G+F) were also considered in Bayesian phylogenetic analysis and the analysis was run for one million generations. ML topology reliability was evaluated by bootstrap, using 1,000 iterations, whilst the sump and sumt commands in Bayesian analysis were used for tabulating posterior probability and building consensus trees. MEGA v.5 software was used for ML analysis and MrBayes v.3.2 software for assessing Bayesian inference. The *P. falciparum* MSP-7H (*Pf*MSP-7H) sequence was used as outgroup in both methods.

### DNA diversity and evolutionary analysis in *pvmsp-7*genes

CLC Main workbench (CLC bio, Cambridge, MA, USA) software was used to assemble forward and reverse sequences from three independent PCR fragments per isolate. Deduced amino acids from Colombian isolates’ *pvmsp-7* sequences and those obtained from several sequencing projects (Sal-I, Brazil-I, Mauritania-I, India-VII and North Korean reference sequences) [4, 32] were aligned using the MUSCLE algorithm [30], followed by manual editing. PAL2NAL software [33] was then used for inferring codon alignments from the aligned amino acid sequences. The T-REKS algorithm [34] was used for searching for repeats having 90% similarity with the deduced *msp-7* amino acid sequences.

DnaSP v.5 software [35] was used for calculating the number of polymorphic segregating sites (Ss), the number of singleton sites (s), the number of parsimony-informative sites (Ps), the number of haplotypes (H), the Watterson estimator (θ^w^) and nucleotide diversity per site (π) for all available sequences (reference sequences and Colombian ones), as well as for the Colombian population alone. Departure from the neutral model was assessed in the Colombian population by frequency spectrum-based tests (Tajima’s D, Fu and Li’s D* and F* statistics Fay and Wu’s H) and tests based on the distribution of haplotypes (Fu’s Fs and K-tests and H-test (for the latter test haplotype diversity obtained from DnaSP software was multiplied by (n-1)/n according to Depaulis and Veuille [35, 36])). DnaSP v.5 and/or ALLELIX software were used for these tests, coalescent simulations being used for obtaining confidence intervals [35]. Positions containing gaps or repeats in the alignment were not taken into account.

Natural selection was assessed by using the modified Nei-Gojobori method [37] which calculated non-synonymous (d_N_) and synonymous (d_S_) rate substitution. Differences between d_N_ and d_S_ were assessed by applying Fisher’s exact test (suitable for d_N_ and d_S_ < 10 [38]) and the Z-test available in MEGA software v.5 [39]. The Datamonkey web server [40] was used for assessing codon sites under positive or negative selection at population level, along with the IFEL codon-based maximum likelihood method [41]. Positive or negatively selected sites were also assessed by FEL, SLAC, REL [42], MEME [43] and FUBAR [44] methods. A <0.1 p-value was considered significant for IFEL, FEL, SLAC and MEME methods, a >50 Bayes factor for REL and a >0.9 posterior probability for FUBAR. Recombination was considered before running these tests. The branch-site REL method was used for identifying branches (lineages) when a percentage of sites have evolved under positive selection for exploring the long-term selection effect. Non-synonymous divergence (K_N_) and synonymous divergence (K_S_) rate substitutions were also calculated using the modified Nei-Gojobori method [37] with Jukes-Cantor correction [45]. Positive and negative selection at every codon for *P. vivax*/*P. cynomolgi msp-7* alignments were also evaluated by FEL, SLAC, MEME, REL and FUBAR methods.

Linkage disequilibrium (LD) was evaluated by calculating the Z_nS_ statistic [46]. Linear regression between LD and nucleotide distances was evaluated to ascertain whether recombination was taking place in *pvmsp-7* genes. Recombination was also assessed by the GARD method [47] and by ZZ [48] and RM tests [49]. RDP3 v3.4 software was used for detecting recombinant fragments in *pvmsp-7* genes [50].

## Results and discussion

### Genotyping natural isolates

The thirty-six samples used in this study were genotyped by PCR-RFLP of the *pvmsp-1* marker. All samples were infected by a single strain (a single *P. vivax msp-1* allele was detected) and considered for PCR amplification of *pvmsp-7* genes. The RFLP pattern confirmed the presence of different genotypes in the isolates so obtained. In spite of all samples amplifying the *pvmsp-1* fragment, no amplimers were detected in some samples for some of the *msp-7* genes (*pvmsp-7E* n = 31, *pvmsp-7F* n = 36 and *pvmsp-7L* n = 31).

### The *msp-7*family structure in *Plasmodium cynomolgi*and phylogenetic analysis

**Figure 1:**
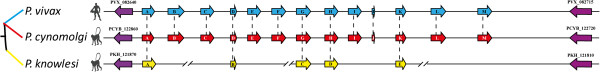
**Schematic representation of the** ***msp-7*** **family in** ***Plasmodium vivax*** **,** ***P. cynomolgi*** **and** ***P. knowlesi*** **.** The genes flanking the *msp-7* chromosome region in these three species are represented by purple boxes. The blue boxes represent *P. vivax msp-7* genes, the red ones represent *P. cynomolgi msp-7* genes and the yellow ones represent *P. knowlesi msp-7* genes. The genes are given in alphabetical order from left to right. The dashed lines connect orthologous genes. All genes are represented to scale, but in *P. cynomolgi* and *P. knowlesi* the distance between them is not representative.

Prior analysis has suggested that there are several *msp-7* species-specific duplications in *P. vivax* [12]. The recent sequencing of the *P. cynomolgi* genome [51], a species phylogenetically close to *P. vivax* [3], has meant that new sequences from this multigene family are now available. The *P. cynomolgi* genomic region flanked by the PCYB_122860 and PCYB_122720 genes contained eleven 0.9 to 1.4 Kb length ORFs having the same transcription orientation. A shorter 0.5 Kb fragment having 30% similarity with the identified ORFs was also observed. A 314 bp region having 75.8% identity with the 285 bp fragment in *P. vivax* between the *pvmsp-7I* and *pvmsp-7K* genes was also found (Figure 1). The *P. cynomolgi msp-7* genes (and/or fragments) were named in alphabetical order, according to their location regarding the PCYB_122860 gene (Figure 1). Contrasting with PlasmoDB annotation, our group found that PcMSP-7C, −7F, −7H, −7I, −7K proteins might be encoded by a single exon like *P. vivax* MSP-7K [52] (Additional file 1). The deduced amino acid sequences from these ORFs had a signal peptide, but no membrane-anchoring regions. The domain characteristic of the MSP-7 family (MSP_7C, Pfam domain ID: PF12948) was absent in the deduced PcMSP-7L protein sequences due to a premature stop codon.

**Figure 2:**
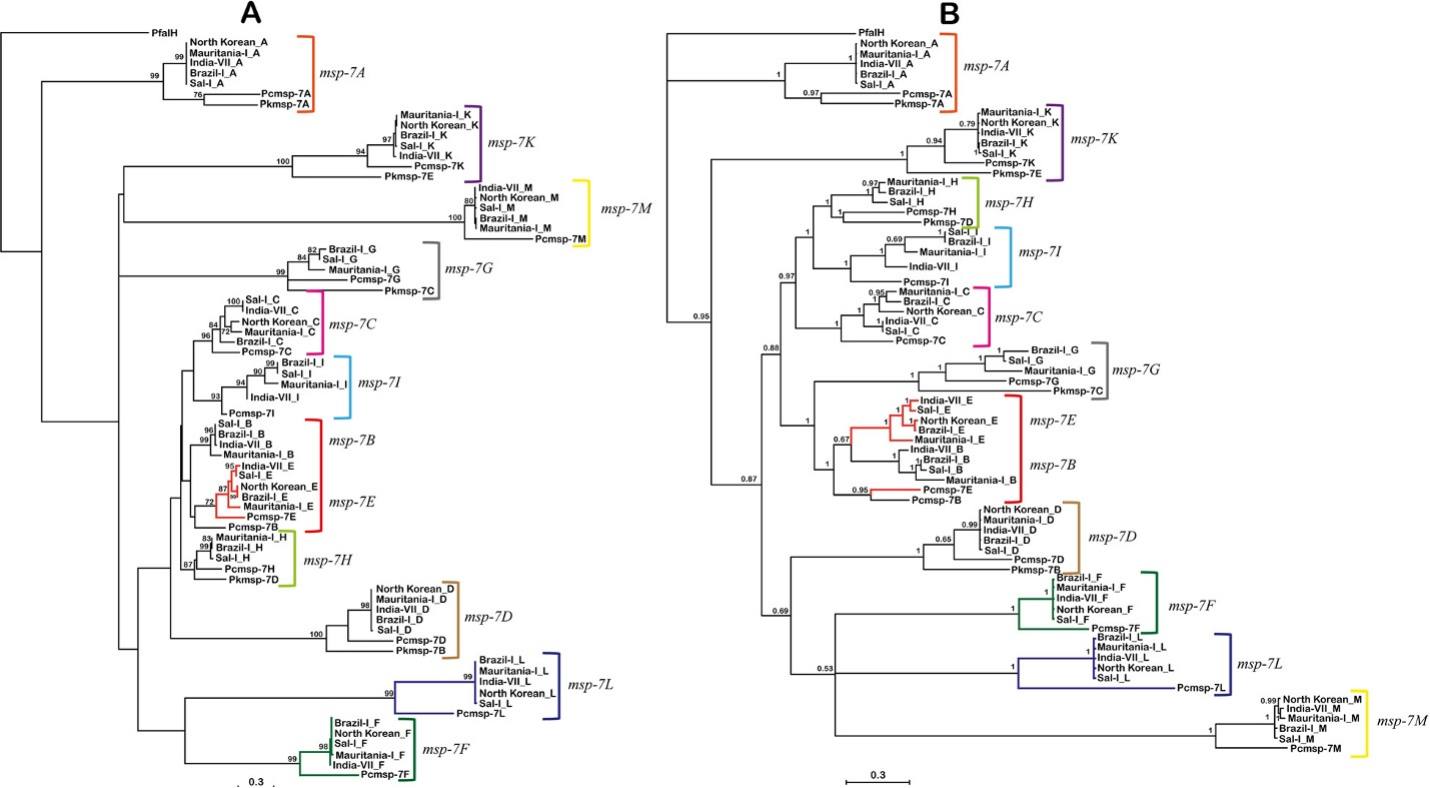
***msp-7*** **gene family phylogeny inferred by Maximum Likelihood (A) and Bayesian (B) methods for** ***P. vivax*** **and two closely related species.** The coloured branches show the MSP-7E (red), MSP-7F (green) and MSP-7L (blue) sequences. Sequence clustering reflects orthologous relationships between the *msp-7* genes from these three species. The *P. falciparum* MSP-7F (PfalH) sequence was used as outgroup. Numbers over the branches are bootstrap and probability values.

Orthologous relationships were established for *P. cynomolgi* MSP-7 (PcMSP-7) sequences by inferring phylogenies, using these sequences together with *P. vivax* (Pv) and *P. knowlesi* (Pk) MSP-7 sequences (Figure 2) with the *P. falciparum* MSP-7H (PfMSP-7H) as outgroup. The topologies revealed eleven clades having good statistical support (Figure 2); each *P. cynomolgi* MSP-7 had a counterpart in *P. vivax* or *P. knowlesi*. However, clustering for PcMSP-7B and PcMSP-7E differed regarding the other MSP-7s. PcMSP-7E formed a group with PvMSP-7E (Figure 2A) in ML topology, even though not having very high statistical support (72%). The BY topology method (Figure 2B) gave a subgroup formed by PcMSP-7B and PcMSP-7E which appeared as an external PvMSP-7B/PvMSP-7E group suggesting that these genes are inparalogous and, therefore, the duplication events occurred after the divergence of *P. cynomolgi* and *P. vivax*. Even though *pcmsp-7B* and *pcmsp-7E* did not form a group with *pvmsp-7B* and *pvmsp-7E*, respectively, their location regarding PVX_082640 and PCYB_122860 genes was the same (Figure 1). Moreover, genetic distances between *pcmsp-7B* and *pcmsp-7E* (and/or *pvmsp-7B* and *pvmsp-7E*) were similar to *pcmsp-7B* and *pvmsp-7B* (or *pcmsp-7E* and *pvmsp-7E*). Furthermore, genetic distance between *pcmsp-7B* and *pvmsp-7B* was smaller than that between *pcmsp-7B* and *pvmsp-7E* and the distance between *pcmsp-7E* and *pvmsp-7E* was less than the distance between *pcmsp-7E* and *pvmsp-7B* (Additional file 2). Consequently, there is little probability that duplication events following the divergence of these two species independently led to the same order of *msp-7B* and *msp-7E* genes. Evidence of gene conversion (mechanism conducting paralogous homogenization) between *P. vivax msp-7* members has been reported previously [21]; if such mechanism occurs between *msp-7B* and *msp-7E* genes it would be expected that they would become clustered in the phylogeny and not with their counterparts from a sister taxon; however, further analysis is needed for confirming such hypothesis.

The aforementioned results have shown that *P. vivax* and *P. cynomolgi* share the whole *msp-7* repertoire described to date, suggesting that the duplications which gave rise to the *msp-7B*, −*7C*, −*7E*, −*7F*, −*7I*, −*7L* and *-7M* genes occurred before the divergence between *P. vivax* and *P. cynomolgi* and after their divergence from *P. knowlesi*, and thus *msp-7B*, −*7C*, −*7E*, −*7F*, −*7I*, −*7L* and *-7M* are not exclusive to *P. vivax*.

### *pvmsp-7E*genetic diversity

168 segregant sites were found in the *pvmsp-7E* gene (Table  1), showing that this gene is highly polymorphic. The nucleotide diversity (π) estimated for this gene (Table  1) was comparable to that found in other genes encoding surface proteins (*pvmsp-1* [53], *pvmsp-3* [54] and *pvmsp-5* [55, 56]) as well as other members of *pvmsp-7* family [21]. Even though genes such as *pvmsp-1*, *pvmsp-3* and *pvmsp-5* are highly polymorphic, their diversity at protein level is usually located in determined regions. These regions are usually immune response targets and have thus tended to evolve more rapidly, accumulating mutations which alter the protein sequence and thereby evading the host’s immune system. Around 60% of the polymorphism found in *pvmsp-7E* was located at the gene’s central region whilst the gene’s ends were relatively high conserved (Additional files 3 and 4). This pattern has been previously observed in other *pvmsp-*7 genes [21].

**Table 1:** **DNA polymorphism measurements for** ***pvmsp-7*** **genes**

n	Gene	Sites	Ss	S	Ps	H	θ^w^(SD)	π (SD)
**Worldwide genetic diversity**								
35	***msp-7E***	1,044	168	8	160	23	0.0390 (0.0030)	0.0573 (0.0040)
41	***msp-7F***	1,164	3	1	2	5	0.0006 (0.0003)	0.0008 (0.0001)
36	***msp-7L***	1,212	5	1	4	7	0.0010 (0.0004)	0.0006 (0.0001)
**Local genetic diversity**								
31	***msp-7E***	1,044	164	6	158	19	0.0393 (0.0031)	0.0558 (0.0045)
36	***msp-7F***	1,176	2	0	2	4	0.0004 (0.0003)	0.0007 (0.0004)
31	***msp-7L***	1,212	4	0	4	6	0.0008 (0.0004)	0.0006 (0.0001)

Ss: number of segregating sites, S: number of singleton sites, Ps: number of parsimony-informative sites, H: number of haplotypes, θ^W^: Watterson estimator, π: nucleotide diversity. (SD): standard deviation. Worldwide genetic diversity: the analysis involved the reference sequences together with the Colombian sequences. Local genetic diversity: Analysis for the Colombian sequences.

Regarding DNA, 23 haplotypes have been found worldwide in *pvmsp-7E* (Table 1 and Additional file 4); fourteen different haplotypes have been found in this gene’s 3′-end, whilst eleven haplotypes have been found in the central region and five in the 5′-end (Additional file 3). Nineteen of these 23 haplotypes were found in the Colombian population (Table 1 and Additional file 4; haplotype 10: 26%; haplotypes 5, 9: 15%; haplotypes 7, 11, 13, 18: 10%; and haplotypes 6, 8, 12, 14–17, 19–23: 5%) over the course of a 3-year period (2007–2010) without any longitudinal or spatial trends. Thirteen haplotypes were found in Colombia at amino acid level (Additional file 5); ten haplotypes having similar frequencies were observed in the *pvmsp-*7*E* central region in the Colombian population whilst three and four haplotypes were distinguished towards the N- and C-terminals, respectively (Additional file 5).

The T-REKS algorithm did not find repeats in the deduced protein sequences. Of the thirty-six sequences analysed for this gene, the North Korean sequence had a premature stop codon, suggesting that *pvmsp-7E* is a pseudogene in this strain; however, the annotation in the Broad institute for the North Korean *pvmsp-7E* gene (accession: PVNG_00513.1) suggests that this could have an intron. Further cDNA analysis is needed to confirm such issue regarding this strain.

### *pvmsp-7E*neutrality and selection tests

Several tests based on the neutral model of molecular evolution were used with *pvmsp-7E* sequences from the Colombian population for evaluating whether this gene deviated from neutral expectations (Table 2 and Additional file 6). Tests based on the polymorphism frequency spectrum had significant values (Table 2). Fu and Li’s D* and F* estimators had values greater than zero whilst Fay and Wu’s H estimator had statistically significant negative values (Table 2), indicating deviation from the neutral model of evolution. In addition, the haplotype distribution-based tests also gave statistically significant values; Fu’s Fs test gave values greater than zero and the haplotype number (18) and haplotype diversity (0.922) were lower than that expected under neutrality (Table 2).

**Table 2:** **Neutrality, linkage disequilibrium and recombination tests for** ***pvmsp-7*** **genes for the Colombian population**

n	Gene	Tajima	Fu and Li		Fay and Wu’s H	Fu’s Fs	K-test	H-test (SD)	Z_nS_	ZZ	RM
		D	D*	F*							
31	***msp-7E***	1.452	1.603**	1.839*	−51.002*	7.501**	18*	0.922 (0.02)*	0.246*	0.453*	12
36	***msp-7F***	1.401	0.783	1.112	−0.365	0.114	4	0.560 (0.07)	0.327	0.000	1
31	***msp-7L***	−0.610	1.054	0.658	0.656	−2.453	6	0.578 (0.08)	0.050	−0.031	1

*msp-7E* haplotype number (K-test) and diversity (H-test) were lower than expected under neutrality. (SD): standard deviation. *: p <0.05, **: p <0.02.

A sliding window for D, D*, F* and H statistics gave values greater than zero (D, D* and F*) in the gene’s central region and lower than zero in the 3′-end, this being the region where the most negative value for H was located (Additional file 7). The gene was divided into three fragments: 5′-end (nucleotide 1 to 390), central (nucleotide 391 to 747) and 3′-end (nucleotide 748 to 1158); the aforementioned neutrality estimators were calculated for each of them (Additional file 6). Fu and Li’s D* and Fu’s Fs tests gave values greater than zero at the 5′-end whilst Tajima’s D, Fu and Li’s D*, F* and Fu’s Fs test scores were greater than zero in the central region. The haplotype number and haplotype diversity were lower than expected under neutrality in the 5′-end and central region. Only Fay and Wu’s H tests gave statistically significant values at the 3′-end (Additional file 6).

**Figure 3:**
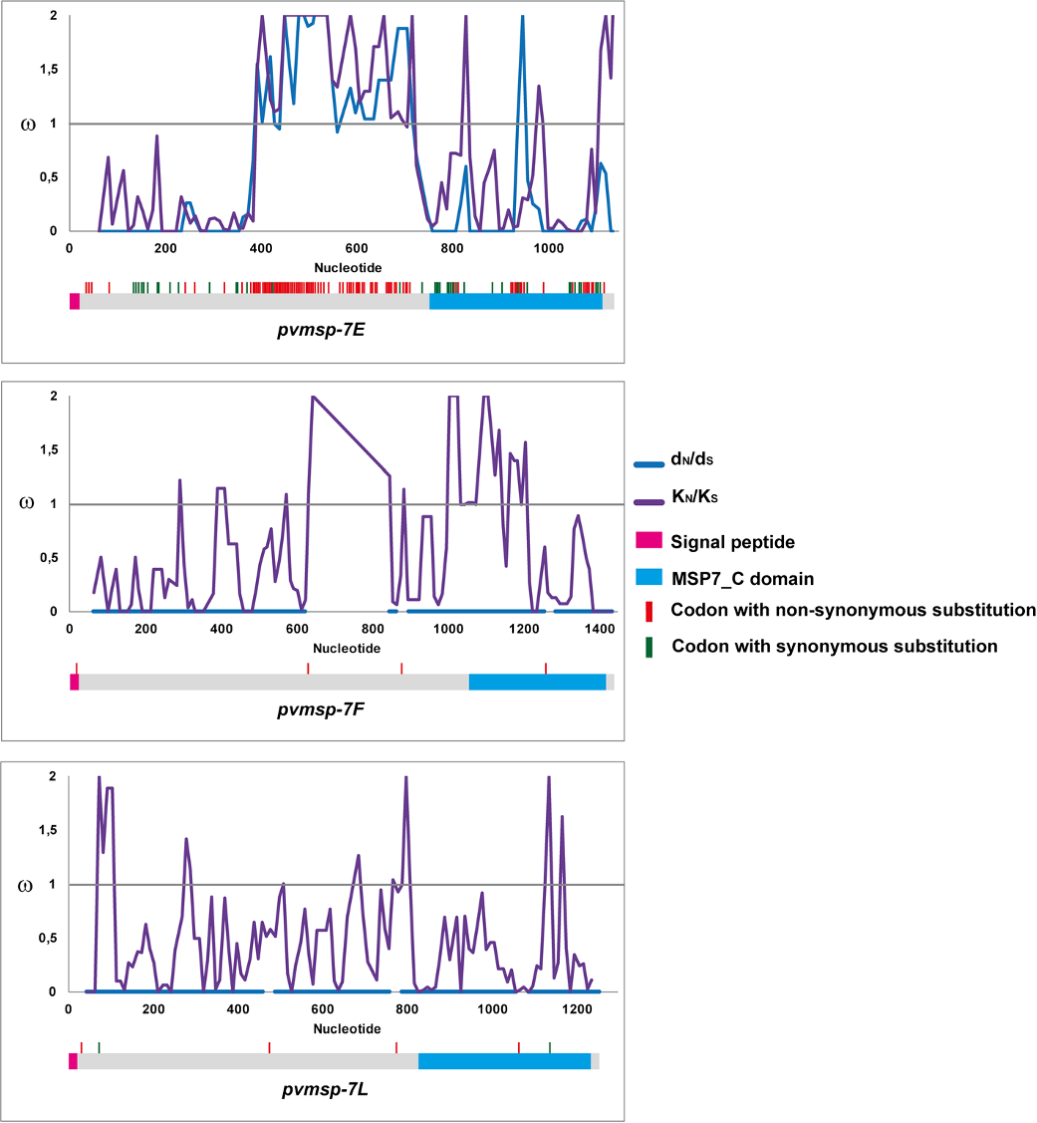
**Sliding window for** **ω** **rate.** *Plasmodium vivax msp-7E*, *msp-7F* and *msp-7L* genes’ ω (d_N_/d_S_) values represented in blue, whilst the divergence omega (ω: K_N_/K_S_) between *P. vivax* and *P. cynomolgi* is shown in purple. A diagram of each gene is given below the sliding window. Intra-species non-synonymous substitutions (red) and synonymous substitutions (green) are shown by vertical lines above each gene.

Natural selection seemed to act in different ways within the *pvmsp-7E* gene. A sliding window for the non-synonymous substitutions per non-synonymous site and synonymous substitutions per synonymous site rate (d_N_/d_S_ = ω) gave values greater than 1 (ω > 1) in the central region and a peak at the 3′-end (Figure 3). The d_S_ rate at the 5′ and 3′-ends was significantly greater than d_N_, whilst d_N_ was significantly greater than d_S_ in this gene’s central region (Table 3). Eight positively selected sites were identified in the central region and one in the 3′-end. Twenty-six negatively selected sites were identified, mainly in the 5′-end (9 sites) and 3′-end (14 sites) (Additional files 4 and 8).

**Table 3:** **Average number of** ***pvmsp-7*** **gene synonymous substitutions per synonymous site (d**_**S**_ **) and non-synonymous substitutions per non-synonymous site (d**_**N**_ **)**

n	Gene	5′-end		Central		3′-end		Full-length gene	
Worldwide isolates		d_S_(SE)	d_N_(SE)	d_S_(SE)	d_N_(SE)	d_S_(SE)	d_N_(SE)	d_S_(SE)	d_N_(SE)
35	***msp-7E***	0.0488 (0.0118)•	0.0082 (0.0032)	0.0717 (0.0163)	0.1573 (0.0134)•	0.0665 (0.0130)•	0.0171 (0.0045)	0.0626 (0.0078)	0.0551 (0.0055)
41	***msp-7F***	0.0000 (0.0000)	0.0000 (0.0000)	0.0000 (0.0000)	0.0013 (0.0012)	0.0000 (0.0000)	0.0018 (0.0018)	0.0000 (0.0000)	0.0011 (0.0007)^
36	***msp-7L***	0.0006 (0.0005)	0.0000 (0.0000)	0.0000 (0.0000)	0.0012 (0.0008)	0.0013 (0.0012)	0.0008 (0.0008)	0.0006 (0.0005)	0.0007 (0.0004)
**Colombian isolates**									
31	***msp-7E***	0.0473 (0.0115)•	0.0079 (0.0032)	0.0706 (0.0168)	0.1549 (0.0135)•	0.0633 (0.0124)•	0.0161 (0.0042)	0.0606 (0.0077)	0.0539 (0.0056)
36	***msp-7F***	0.0000 (0.0000)	0.0000 (0.0000)	0.0000 (0.0000)	0.0012 (0.0011)	0.0000 (0.0000)	0.0017 (0.0016)	0.0000 (0.0000)	0.0010 (0.0007)^
31	***msp-7L***	0.0000 (0.0000)	0.0000 (0.0000)	0.0000 (0.0000)	0.0012 (0.0009)	0.0010 (0.0010)	0.0009 (0.0008)	0.0004 (0.0004)	0.0007 (0.0004)

SE: standard error. 5′-end (*pvmsp-7E*: nucleotide 1–390, *pvmsp-7F*: nucleotide 1–432, *pvmsp-7L*: nucleotide 1–381), central (*pvmsp-7E*: nucleotide 391–747, *pvmsp-7F*: nucleotide 433–1,053, *pvmsp-7L*: nucleotide 382–816) and 3′-end (*pvmsp-7E*: nucleotide 748–1,158, *pvmsp-7F*: nucleotide 1,054–1,449, *pvmsp-7L*: nucleotide 817–1,275). Numbering based on Additional files 4, 9 and 10. ^: p = 0.06, •: p <0.001.

These results suggested that the central region was under natural positive selection. According to Tajima and Fu and Li tests (which had significant values higher than zero) *pvmsp-7E* seemed to be under balancing selection favouring the existence of different alleles in the population. This type of selection frequently occurs in antigens exposed to the immune system; immune responses therefore seemed to be directed towards the *pvmsp-7E* central region in which the mutations were accumulated at a greater rate by positive selection (Additional files 4 and 8).

**Figure 4:**
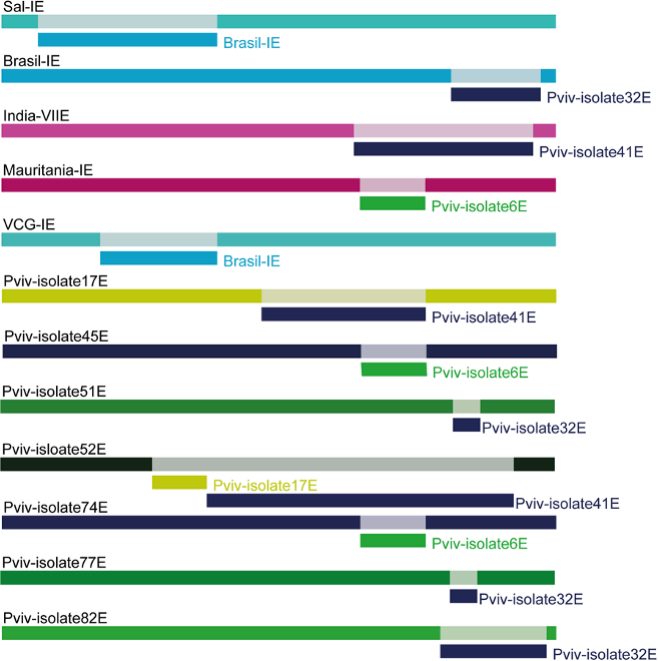
**Schematic representation of recombination segments identified in** ***pvmsp-7E*** **by RDP3.** Recombination fragments were only found in *pvmsp-7E*. Each variant is represented by a colour bar; a segment having a different colour below the bar represents the recombination event so identified.

Interestingly, as Fay and Wu’s H tests gave significant negative values and as K-test and H-test were lower than expected under neutrality (Table 2), then a selective sweep would be probable and strong LD and low genetic diversity would thus be expected. Z_nS_ values suggested that *pvmsp-7E* had non-random polymorphism association, as expected in a selective sweep (Table 2). However, differently to what was expected, *pvmsp-7* had high genetic diversity (Table 1). When recombination is present in a locus under selective sweep, then it would be expected that genetic diversity would only become reduced close to the selection site [57]. There was evidence of recombination throughout the *pvmsp-7E* gene (Table 1 and Figure 4, see below) and since the deepest H “valley” was located at the 3′-end (Additional file 7), the selective sweep may not have affected the gene completely but just this region. The 5′-end and central regions showed no evidence of selective sweep (Additional file 6) and π was high in the central region and low at the 5′ extreme (Additional file 3). The 3′-end had significant values in H and Z_nS_ tests (Additional file 6), suggesting that the selection site should have been located in this region. The π value in 3′-end was considerably reduced regarding that for the central region (Additional file 3). However, the number of SNPs seems to be higher than that expected in a selective sweep. Our results suggested that a selective sweep affected the *pvmsp-7E* gene, the selection site was located in the gene’s 3′-end and this seemed to be an incomplete selective sweep due to the presence of recombination since not all variability had been lost. However, if the selective sweep has not been recent, new mutations could have become fixed following such sweep [57].

Fu’s Fs test gave values greater than zero (Table 2 and Additional file 6) which may have resulted from a reduction in haplotypes due to a recent bottleneck. Consequently, a low genetic diversity throughout the gene pool is expected in *P. vivax* Colombia population. Prior studies have shown high genetic diversity in parasitic antigens in the Colombian population [21, 55], meaning that such demographic event is highly unlikely. This test’s result may have been due to a reduction of *pvmsp-7E* haplotypes by the selective sweep, causing the number of haplotypes to be lower than that expected.

### *pvmsp-7F*and *pvmsp-7L*genetic diversity

In contrast to *pvmsp-7E*, *pvmsp-7F* and *pvmsp-7L* had low genetic diversity (Table 1). This genetic diversity was similar to that observed in *pvmsp-4* [58, 59], *pvmsp-8* [60], *pvmsp-10* [22, 60], *pv12*, *pv38* [61], *pv41* [62], as well as in *pvmsp-7A* and *pvmsp-7K* [22]. Aligning the Colombian sequences with those obtained from the databases (reference sequences, Additional files 9 and 10) revealed that these genes only had four and six segregant sites, respectively. The π values and the number of haplotypes for these genes were low (Table 1). The most frequently occurring *pvmsp-7F* allele in Colombia was haplotype 2 (61%), followed by haplotype 1 (19%), haplotype 3 (17%) and haplotype 4 (3%), whilst haplotype 1 (61%) was the most frequent for *pvmsp-7L*, followed by haplotype 3 (16%), haplotype 2 (14%) and haplotypes 4, 5 and 6 (3%).

### *pvmsp-7F*and *pvmsp-7L*neutrality and selection tests

Neutrality for *pvmsp-7F* and *-7L* genes in the Colombian population could not be ruled out as no statistically significant values were found for the tests based on the neutral model of molecular evolution (Table 2 and Additional file 6). Likewise, no natural selection signals were found to be acting on these genes when d_N_ and d_S_ rates were calculated (Table 3). However, when the effect of selection on each codon was evaluated, it was seen that codon 424 regarding *pvmsp-7F*, was under positive selection. Concerning *pvmsp-7L*, codons 159, 260 and 357 showed positive selection signals (Additional files 8, 9 and 10).

### Intragene linkage disequilibrium (LD) and recombination in *pvmsp-7*genes

As mentioned above, there were non-random associations regarding polymorphism for *pvmsp-7E* according to the Z_nS_ test (Table 2 and Additional file 6). No evidence of LD was found in *pvmsp-7F* or *pvmsp-7L* (Table 2 and Additional file 6), indicating that polymorphism within these genes was not associated. A linear regression between LD and nucleotide distance for *pvmsp-7s* gave a line sloping downwards as nucleotide distance increased in *pvmsp-7E*, suggesting intragene recombination. Twelve minimal recombination (RM) events were found for *pvmsp-7E* whilst only one RM was found in *pvmsp-7F* and *pvmsp-7L* (Table 2). The ZZ test and GARD method suggested recombination in *pvmsp-7E* (ZZ = p < 0.05 and GARD 2 breakpoints, p < 0.0004) but not in *pvmsp-7F* or *pvmsp-7L*. Figure 4 shows the fragments produced by recombination in *pvmsp-7E*.

### *pcmsp-7*and *pvmsp-7*genes appear to have diverged by positive selection

**Figure 5:**
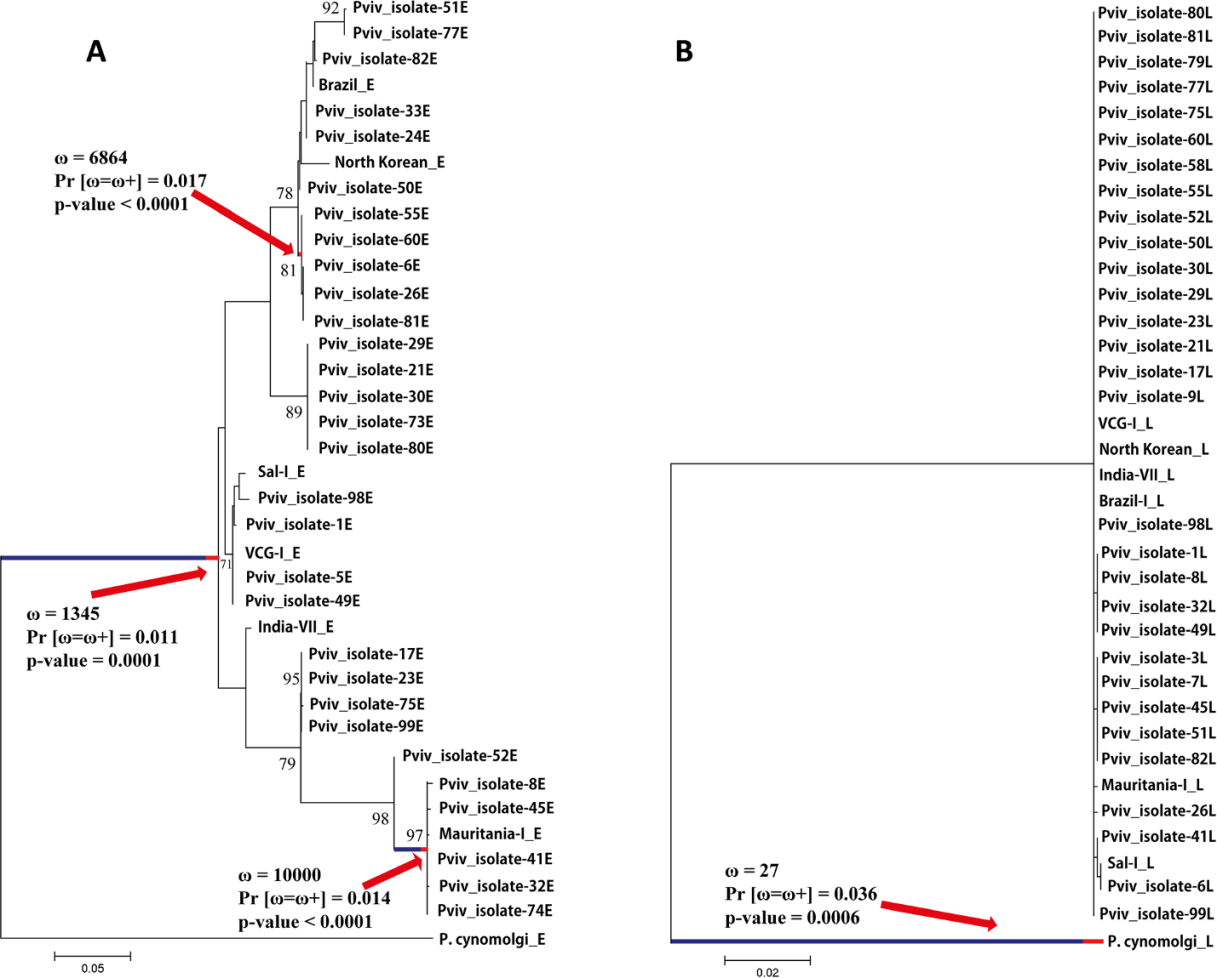
**Positive lineage-specific selection in** ***msp-7E*** **(A) and** ***msp-7L*** **(B) genes.** Branches under diversifying selection were identified by Branch-site REL method. ω values, the percentage of selected sites (Pr [ω = ω+]) and p-values are shown.

Natural selection’s long-term effect on evolutionary history can be evaluated by comparing the orthologous genes from phylogenetically-related species [60–64]. *msp-7E* was highly divergent when compared to the *pvmsp-7E* and *pcmsp-7E* genes (Figure 3). In spite of *msp-7F* and *msp-7L* being highly conserved in *P. vivax*, they also have been shown to be highly divergent when compared to *P. cynomolgi* orthologous genes (Figure 3). The random effects branch-site model (Branch-site REL) was performed for determining how natural selection had acted during *P. vivax* and *P. cynomolgi* evolutionary history. This test displayed lineage-specific diversifying selection signals in *msp-7E* and *msp-7L* (ω > 1, Figure 5). Moreover, the sliding window for the non-synonymous divergence per non-synonymous site rate (K_N_) and the synonymous divergence per synonymous site rate (Ks) (divergent omega, K_N_/Ks = ω) gave highly divergent areas in the central region and 3′-end of these three genes (Figure 3). A statistically significant K_N_ > K_S_ was found in the central region (p < 0.001) in *pvmsp-7E* (Table 4). No significant values were found in *msp-7F*, but in *msp-7L*, K_S_ was significantly higher than K_N_ (Table 4). However, when intraspecific polymorphism was compared to interspecific divergence using the McDonald-Kreitman test (MKT) no statistically significant values were found for these genes. The methods for estimating ω values for each codon (SLAC, FEL, REL, MEME and FUBAR) identified twenty-five (for *msp-7E*), four (for *msp-7F*) and seven (for *msp-7L*) codons under positive selection between *pvmsp-7* and *pcmsp-7* sequences (Additional files 4, 9, 10 and 11).

**Table 4:** **Average number of** ***msp-7*** **gene synonymous divergence per synonymous site (K**_**S**_ **) and non-synonymous divergence per non-synonymous site (K**_**N**_ **)**

n	Gene	5′ end		Central		3′ end		Full-length gene	
Worldwide isolates		K_S_(SE)	K_N_(SE)	K_S_(SE)	K_N_(SE)	K_S_(SE)	K_N_(SE)	K_S_(SE)	K_N_(SE)
36	***msp-7E***	0.0746 (0.0156)•	0.0126 (0.0034)	0.0813 (0.0185)	0.1976 (0.0191)•	0.0980 (0.0163)•	0.0299 (0.0056)	0.0833 (0.0092)	0.0676 (0.0066)
42	***msp-7F***	0.0098 (0.0023)†	0.0047 (0.0009)	0.0146 (0.0029)	0.0141 (0.0022)	0.0086 (0.0022)	0.0100 (0.0022)	0.0110 (0.0014)	0.0096 (0.0010)
37	***msp-7L***	0.0140 (0.0031)‡	0.0092 (0.0017)	0.0172 (0.0035)	0.0149 (0.0020)	0.0111 (0.0025)‡	0.0070 (0.0013)	0.0139 (0.0017)*	0.0102 (0.0010)
**Colombian isolates**									
32	***msp-7E***	0.0755 (0.0151)•	0.0128 (0.0032)	0.0808 (0.0183)	0.1963 (0.0191)•	0.0982 (0.0156)•	0.0305 (0.0053)	0.0834 (0.0089)	0.0676 (0.0065)
37	***msp-7F***	0.0111 (0.0027)†	0.0053 (0.0011)	0.0165 (0.0033)	0.0156 (0.0023)	0.0098 (0.0025)	0.0110 (0.0022)	0.0125 (0.0016)	0.0107 (0.0011)
32	***msp-7L***	0.0156 (0.0037)‡	0.0107 (0.0020)	0.0199 (0.0041)	0.0170 (0.0023)	0.0123 (0.0027)‡	0.0081 (0.0015)	0.0157 (0.0019)*	0.0118 (0.0011)

SE: standard error. 5′-end (*pvmsp-7E*: nucleotide 1–390, *pvmsp-7F*: nucleotide 1–432, *pvmsp-7L*: nucleotide 1–381), central (*pvmsp-7E*: nucleotide 391–747, *pvmsp-7F*: nucleotide 433–1,053, *pvmsp-7L*: nucleotide 382–816) and 3′-end (*pvmsp-7E*: nucleotide 748–1,158, *pvmsp-7F*: nucleotide 1,054–1,449, *pvmsp-7L*: nucleotide 817–1,275). Numbering based on Additional files 4, 9 and 10. ‡: p <0.09, *: p <0.04, †: p <0.02, •: p <0.001.

These results suggested that these genes have become diversified by positive selection; a similar pattern which have been reported for the *pvmsp-1* gene [26, 27]. Divergence due to positive selection in *msp-1* coinciding with Asian macaque radiation [26, 65] 3 to 6 million years ago means that divergence by positive selection in *msp-1* appears to be the result of adaptations to available new hosts [26, 65]. *P. falciparum* MSP-1 and MSP-7 form a protein complex involved in invasion [9, 10]. Assuming the formation of a protein complex between MSP-1 and MSP-7 in *P. vivax*, MSP-7s would be under the same selective pressures and may thus have evolved in a similar way. Theoretically [66, 67], it has been suggested that a strong selective sweep may result in population differentiation at the hitchhiking locus, provided that the gene flow between these populations is low. Since malarial parasites could become diversified by sympatric events [68, 69], *msp-7* (similar to *msp-1*) may have become diversified by positive selection (Figure 5) as a mechanism for adapting the ancestral *P. vivax* population to a new host during the switch to humans [70] and thus the selective sweep detected in *msp-7E* might have been an effect of such adaptation.

### Negative selection within and between species supports the idea that the 3′-end encodes the functional region in MSP-7 proteins

In spite of divergence by positive selection, *msp-7* functional regions could have evolved more slowly due to their role during invasion and thus the accumulation of substitutions would have been mainly synonymous. K_S_ > K_N_ was revealed in *msp-7E* and *msp-7L* when comparing *P. vivax* and *P. cynomolgi* sequences (Table 4). Fifty-seven sites were revealed to be under negative selection in *msp-7E*, twenty-four in *msp-7F* and thirty-six in *msp-7L* (Additional files 4, 9, 10 and 11). A large percentage of negatively selected sites were located in the gene’s 3′-end encoding the *msp-7* family’s characteristic domain (MSP7_C, Pfam domain ID: PF12948). The protein’s C-terminal region encoded by these genes was highly conserved in *pvmsp-7A*, −*7C*, −*7H*, −*7I*, −*7K* [21, 22], −*7E*, −*7F* and *-7L*; furthermore, this region has been conserved for a long period of time (2.6 to 5.2 million years ago [3]), at least in *msp-7E* (84.8% similarity between *P. vivax* and *P. cynomolgi*), −*7F* (86.8%) and *-7L* (95.4%). The negative selection signals identified at the 3’-end of these three genes (Additional files 8 and 11) suggested that the biological structure encoded by this region has been stable slowly evolving since divergence between *P. vivax* and *P. cynomolgi* due to its functional importance. These results support the idea that this region encodes this family’s functional domain [21].

### The *pvmsp-7*and *pcmsp-7*sequences have different gene structures

Marked differences were observed between *P. vivax* and *P. cynomolgi msp-7* genes. *pcmsp-7F* had a long insertion (one hundred ninety-two nucleotides) compared to *pvmsp-7F* (Additional file 9); however, the ORF remained open. *pcmsp-7L* had a premature stop codon caused by the deletion of one or two nucleotides from the sequence (Additional file 10). The protein encoded by this gene thus had no domain characteristic of this family (MSP7_C, Pfam domain ID: PF12948); however, many synonymous substitutions between species were observed in the region encoding this domain (the gene’s 3‵-end) when *P. vivax* and *P. cynomolgi* sequences were compared. Thirteen sites in this region were under negative selection in *msp-7L* (Additional files 10 and 11). The GeneScan algorithm [71] was then used for searching for exon/intron splice sites in *pcmsp-7F* and *pcmsp-7L* sequences. GeneScan analysis revealed regions which could act as donor and acceptor sequences in *pcmsp-7L* but not in *pcmsp-7F*. There was a thymine in *pcmsp-7L* nucleotide 609, whilst there was a cytosine in the homologous position in its orthologue in *P. vivax* (nucleotide 615 in the Sal-I sequence). Such change may have produced a putative donor (GT) site in *pcmsp-7L* whilst a putative acceptor site was located in position 1,030/1,031 (Additional file 12); an intron region was thus located in *pcmsp-7L* between nucleotides 608 and 1,031. Such exon-intron-exon structure in *pcmsp-7L* can be observed in the annotation of the *P. cynomolgi* genome available from PlasmoDB; however, the intron predicted in PlasmoDB was shorter than that predicted by GeneScan. This exon-intron-exon structure allowed *pcmsp-7L* to encode a protein having the MSP7_C domain.

## Conclusions

Our results confirmed that the *P. vivax msp-7* family has a heterogeneous genetic diversity pattern. Some members were seen to be highly conserved whilst other had high genetic diversity. Consequently, *P. vivax msp-7* genes must have evolved differently from those in *P. falciparum* which have low polymorphism [23, 24]. The PvMSP-7s C-terminal region (the gene’s 3′-end) tended to be conserved within and between genes [21]. This region’s conservation tended to be maintained by negative selection in *msp-7E*, *msp-7F* and *msp-7L*, suggesting that this is the functional region for this group of proteins. On the other hand, PvMSP-7 highly diverse members (*pvmsp-7C*, −*7H*, −*7I* [21] and *-7E*) were seen to have undergone rapid evolution at the protein’s central region; immune responses would thus been directed towards this portion of the protein. New alleles have consequently arisen in the population and been maintained by balancing selection as a mechanism for evading an immune response. In addition to this type of evasion, the *P. vivax msp-7* family (similar to that suggested for the *pvmsp-3* family [72]) would follow a model of multi-allele diversifying selection where functionally redundant paralogues [12] would increase evasion of the immune responses by antigenic diversity.

Our results have shown that *P. vivax* and *P. cynomolgi* share the whole *msp-7* repertoire described to date and have revealed lineage-specific positive selection signals which are similar to those reported for *pvmsp-1*. Mutations occurring in *msp-7s* genes during host switch may thus have succeeded in adapting the ancestral *P. vivax* parasite to humans.

## Electronic supplementary material

Additional file 1: **Putative** ***P. cynomolgi msp-7*** **gene sequences obtained from chromosome 12, whole genome shotgun sequence GenBank accession number: NC_020405.** ORF Finder and Gene Runner software were used to identify open reading frames encoding *P. cynomolgi* MSP-7 proteins. (TXT 13 KB)

Additional file 2: **Genetic distance between** ***pcmsp-7B*** **,** ***pcmsp-7E*** **and** ***pvmsp-7B*** **,** ***pvmsp-7E*** **sequences from 5** ***P. vivax isolates.*** The number of nucleotide differences per site was estimated as well as the standard error regarding the *pvmsp-7E* and *pvmsp-7B* reference sequences and the *pcmsp-7E* and *pcmsp-7B* sequences. (PDF 186 KB)

Additional file 3: **DNA polymorphism measurements at the 5′-end, central region and 3′-end for** ***pvmsp*** **-** ***7*** **genes in the Colombian population.** Ss: number of segregating sites, S: number of singleton sites, Ps: number of parsimony-informative sites, H: number of haplotypes, θ^W^: Watterson estimator, π: nucleotide diversity. (SD): standard deviation. 5′-end (*pvmsp-7E*: nucleotide 1–390, *pvmsp-7F*: nucleotide 1–432, *pvmsp-7L*: nucleotide 1–381), central (*pvmsp-7E*: nucleotide 391–747, *pvmsp-7F*: nucleotide 433–1,053, *pvmsp-7L*: nucleotide 382–816) and 3′-end (*pvmsp-7E*: nucleotide 748–1,158, *pvmsp-7F*: nucleotide 1,054–1,449, *pvmsp-7L*: nucleotide 817–1,275). Numbers based on Additional files 4, 9 and 10. (PDF 212 KB)

Additional file 4: ***pvmsp-7E*** **gene alignment.** The alignment shows the 23 haplotypes found in *pvmsp-7E* together with the *pcmsp-7E* haplotype. Haplotype 1, Sal-I; haplotype 2, Brazil-I; haplotype 3, India-VII; haplotype 4, Mauritania-I; haplotype 5–23, Colombian isolates. Dots represent nucleotide identity. Codons under positive selection are shown in green (intra-species) and turquoise (inter-species) and those under negative selection are shown in yellow (intra-species) and fuchsia (inter-species). (PDF 162 KB)

Additional file 5: **Haplotype alignment at PvMSP-7E protein level in Colombia.** The alignment shows the 13 haplotypes at protein level found in PvMSP-7E. Dots represent amino acid identity. (PDF 74 KB)

Additional file 6: **Neutrality, linkage disequilibrium and recombination tests at the 5′-end, central region and 3′-end for** ***pvmsp-7*** **genes in the Colombian population.** 5′-end (*pvmsp-7E*: nucleotide 1–390, *pvmsp-7F*: nucleotide 1–432, *pvmsp-7L*: nucleotide 1–381), central (*pvmsp-7E*: nucleotide 391–747, *pvmsp-7F*: nucleotide 433–1,053, *pvmsp-7L*: nucleotide 382–816) and 3′-end (*pvmsp-7E*: nucleotide 748–1,158, *pvmsp-7F*: nucleotide 1,054–1,449, *pvmsp-7L*: nucleotide 817–1,275). Numbers based on Additional files 4, 9 and 10. •: p <0.02, *: p <0.05. (PDF 203 KB)

Additional file 7: **Neutrality test sliding window for the** ***pvmsp-7E*** **gene.** Tajima’s D (blue), Fu and Li’s D* (red), F* (green) and Fay and Wu’s H (purple). The gene was divided into 3 regions: the 5′-end (nucleotide 1 to 390), central (nucleotide 391 to 747) and 3′-end region (nucleotide 748 to 1,158). The bars at the bottom indicate that a test gave significant values in each region. Numbering based on the alignment shown in Additional file 4. (TIFF 523 KB)

Additional file 8: **Intra-species positively and negatively selected sites detected for** ***pvmsp-7*** **genes.** 5′-end (*pvmsp-7E*: nucleotide 1–390, *pvmsp-7F*: nucleotide 1–432, *pvmsp-7L*: nucleotide 1–381), central (*pvmsp-7E*: nucleotide 391–747, *pvmsp-7F*: nucleotide 433–1,053, *pvmsp-7L*: nucleotide 382–816) and 3′-end (*pvmsp-7E*: nucleotide 748–1,158, *pvmsp-7F*: nucleotide 1,054–1,449, *pvmsp-7L*: nucleotide 817–1,275). Numbers based on Additional files 4, 9 and 10. (PDF 57 KB)

Additional file 9: ***pvmsp-7F*** **gene alignment.** The alignment shows the 8 haplotypes found in *pvmsp-7F* together with *pcmsp-7F* haplotype. Haplotype 1, Sal-I; haplotype 2, Brazil-I and North Korea; haplotype 3, India-VII; haplotype 4, Mauritania-I; haplotypes 5–8, Colombian isolates. Dots represent nucleotide identity. Codons under positive selection are shown in green (intra-species) and turquoise (inter-species) and those under negative selection are shown in fuchsia (inter-species). (PDF 99 KB)

Additional file 10: ***pvmsp-7L*** **gene alignment.** The alignment shows the 7 haplotypes found in *pvmsp-7L* together with *pcmsp-7L* haplotype. Haplotype 1, Sal-I; haplotype 2, Brazil-I, India-VII and North Korea; haplotype 3, Mauritania-I; haplotypes 4–7, Colombian isolates. The dots represent nucleotide identity. Codons under positive selection are shown in green (intra-species) and in turquoise (inter-species) and those under negative selection are shown in fuchsia (inter-species). (PDF 91 KB)

Additional file 11: **Inter-species positively and negatively selected sites detected for** ***msp-7*** **genes.** 5′-end (*msp-7E*: nucleotide 1–390, *msp-7F*: nucleotide 1–432, *msp-7L*: nucleotide 1–381), central (*msp-7E*: nucleotide 391–747, *msp-7F*: nucleotide 433–1,053, *msp-7L*: nucleotide 382–816) and 3′-end (*msp-7E*: nucleotide 748–1,158, *msp-7F*: nucleotide 1,054–1,449, *msp-7L*: nucleotide 817–1,275). Numbers based on Additional files 4, 9 and 10. (PDF 58 KB)

Additional file 12: ***pcmsp-7L*** **putative donor and acceptor sites.** An alignment was made between the Sal-I strain *pvmsp-7L* sequences, *pcmsp-7L* and the sequence resulting from GeneScan analysis (*pcmsp-7L*_mRNA). The red arrows indicate the putative donor and acceptor sites in *pcmsp-7L*. (PDF 62 KB)

## References

[1] Singh J, Purohit B, Desai A, Savardekar L, Shanbag P, Kshirsagar N (2013). Clinical Manifestations, treatment, and outcome of hospitalized patients with *Plasmodium vivax* malaria in two Indian States: A Retrospective Study. Malar Res Treat.

[2] Jain V, Agrawal A, Singh N (2013). Malaria in a tertiary health care facility of Central India with special reference to severe vivax: implications for malaria control. Pathog Glob Health.

[3] Pacheco MA, Battistuzzi FU, Junge RE, Cornejo OE, Williams CV, Landau I, Rabetafika L, Snounou G, Jones-Engel L, Escalante AA (2011). Timing the origin of human malarias: the lemur puzzle. BMC Evol Biol.

[4] Carlton JM, Adams JH, Silva JC, Bidwell SL, Lorenzi H, Caler E, Crabtree J, Angiuoli SV, Merino EF, Amedeo P, Cheng Q, Coulson RM, Crabb BS, Del Portillo HA, Essien K, Feldblyum TV, Fernandez-Becerra C, Gilson PR, Gueye AH, Guo X, Kang’a S, Kooij TW, Korsinczky M, Meyer EV, Nene V, Paulsen I, White O, Ralph SA, Ren Q, Sargeant TJ (2008). Comparative genomics of the neglected human malaria parasite *Plasmodium vivax*. Nature.

[5] Iyer J, Gruner AC, Renia L, Snounou G, Preiser PR (2007). Invasion of host cells by malaria parasites: a tale of two protein families. Mol Microbiol.

[6] Chitnis CE, Blackman MJ (2000). Host cell invasion by malaria parasites. Parasitol Today.

[7] Rodriguez LE, Urquiza M, Ocampo M, Curtidor H, Suarez J, Garcia J, Vera R, Puentes A, Lopez R, Pinto M, Rivera Z, Patarroyo ME (2002). *Plasmodium vivax* MSP-1 peptides have high specific binding activity to human reticulocytes. Vaccine.

[8] Urquiza M, Rodriguez LE, Suarez JE, Guzman F, Ocampo M, Curtidor H, Segura C, Trujillo E, Patarroyo ME (1996). Identification of *Plasmodium falciparum* MSP-1 peptides able to bind to human red blood cells. Parasite Immunol.

[9] Kauth CW, Woehlbier U, Kern M, Mekonnen Z, Lutz R, Mucke N, Langowski J, Bujard H (2006). Interactions between merozoite surface proteins 1, 6, and 7 of the malaria parasite *Plasmodium falciparum*. J Biol Chem.

[10] Pachebat JA, Ling IT, Grainger M, Trucco C, Howell S, Fernandez-Reyes D, Gunaratne R, Holder AA (2001). The 22 kDa component of the protein complex on the surface of *Plasmodium falciparum* merozoites is derived from a larger precursor, merozoite surface protein 7. Mol Biochem Parasitol.

[11] Trucco C, Fernandez-Reyes D, Howell S, Stafford WH, Scott-Finnigan TJ, Grainger M, Ogun SA, Taylor WR, Holder AA (2001). The merozoite surface protein 6 gene codes for a 36 kDa protein associated with the *Plasmodium falciparum* merozoite surface protein-1 complex. Mol Biochem Parasitol.

[12] Garzon-Ospina D, Cadavid LF, Patarroyo MA (2010). Differential expansion of the merozoite surface protein (msp)-7 gene family in Plasmodium species under a birth-and-death model of evolution. Mol Phylogenet Evol.

[13] Garcia Y, Puentes A, Curtidor H, Cifuentes G, Reyes C, Barreto J, Moreno A, Patarroyo ME (2007). Identifying merozoite surface protein 4 and merozoite surface protein 7 *Plasmodium falciparum* protein family members specifically binding to human erythrocytes suggests a new malarial parasite-redundant survival mechanism. J Med Chem.

[14] Kadekoppala M, O’Donnell RA, Grainger M, Crabb BS, Holder AA (2008). Deletion of the *Plasmodium falciparum* merozoite surface protein 7 gene impairs parasite invasion of erythrocytes. Eukaryot Cell.

[15] Tewari R, Ogun SA, Gunaratne RS, Crisanti A, Holder AA (2005). Disruption of *Plasmodium berghei* merozoite surface protein 7 gene modulates parasite growth in vivo. Blood.

[16] Mello K, Daly TM, Morrisey J, Vaidya AB, Long CA, Bergman LW (2002). A multigene family that interacts with the amino terminus of Plasmodium MSP-1 identified using the yeast two-hybrid system. Eukaryot Cell.

[17] Chen JH, Jung JW, Wang Y, Ha KS, Lu F, Lim CS, Takeo S, Tsuboi T, Han ET (2010). Immunoproteomics profiling of blood stage *Plasmodium vivax* infection by high-throughput screening assays. J Proteome Res.

[18] Wang L, Crouch L, Richie TL, Nhan DH, Coppel RL (2003). Naturally acquired antibody responses to the components of the *Plasmodium falciparum* merozoite surface protein 1 complex. Parasite Immunol.

[19] Woehlbier U, Epp C, Hackett F, Blackman MJ, Bujard H (2010). Antibodies against multiple merozoite surface antigens of the human malaria parasite *Plasmodium falciparum* inhibit parasite maturation and red blood cell invasion. Malar J.

[20] Mello K, Daly TM, Long CA, Burns JM, Bergman LW (2004). Members of the merozoite surface protein 7 family with similar expression patterns differ in ability to protect against *Plasmodium yoelii* malaria. Infect Immun.

[21] Garzon-Ospina D, Lopez C, Forero-Rodriguez J, Patarroyo MA (2012). Genetic diversity and selection in three *Plasmodium vivax* merozoite surface protein 7 (Pvmsp-7) genes in a Colombian population. PLoS One.

[22] Garzon-Ospina D, Romero-Murillo L, Tobon LF, Patarroyo MA (2011). Low genetic polymorphism of merozoite surface proteins 7 and 10 in Colombian *Plasmodium vivax* isolates. Infect Genet Evol.

[23] Roy SW, Weedall GD, da Silva RL, Polley SD, Ferreira MU (2009). Sequence diversity and evolutionary dynamics of the dimorphic antigen merozoite surface protein-6 and other Msp genes of *Plasmodium falciparum*. Gene.

[24] Tetteh KK, Stewart LB, Ochola LI, Amambua-Ngwa A, Thomas AW, Marsh K, Weedall GD, Conway DJ (2009). Prospective identification of malaria parasite genes under balancing selection. PLoS One.

[25] Bozdech Z, Mok S, Hu G, Imwong M, Jaidee A, Russell B, Ginsburg H, Nosten F, Day NP, White NJ, Carlton JM, Preiser PR (2008). The transcriptome of *Plasmodium vivax* reveals divergence and diversity of transcriptional regulation in malaria parasites. Proc Natl Acad Sci U S A.

[26] Sawai H, Otani H, Arisue N, Palacpac N, de Oliveira ML, Pathirana S, Handunnetti S, Kawai S, Kishino H, Horii T, Tanabe K (2010). Lineage-specific positive selection at the merozoite surface protein 1 (msp1) locus of *Plasmodium vivax* and related simian malaria parasites. BMC Evol Biol.

[27] Tanabe K, Escalante A, Sakihama N, Honda M, Arisue N, Horii T, Culleton R, Hayakawa T, Hashimoto T, Longacre S, Pathirana S, Handunnetti S, Kishino H (2007). Recent independent evolution of msp1 polymorphism in *Plasmodium vivax* and related simian malaria parasites. Mol Biochem Parasitol.

[28] Imwong M, Pukrittayakamee S, Gruner AC, Renia L, Letourneur F, Looareesuwan S, White NJ, Snounou G (2005). Practical PCR genotyping protocols for *Plasmodium vivax* using *Pvcs* and *Pvmsp1*. Malar J.

[29] *ORF Finder (Open Reading Frame Finder)*. [http://www.ncbi.nlm.nih.gov/projects/gorf/]

[30] Edgar RC (2004). MUSCLE: multiple sequence alignment with high accuracy and high throughput. Nucleic Acids Res.

[31] Abascal F, Zardoya R, Posada D (2005). ProtTest: selection of best-fit models of protein evolution. Bioinformatics.

[32] Neafsey DE, Galinsky K, Jiang RH, Young L, Sykes SM, Saif S, Gujja S, Goldberg JM, Young S, Zeng Q, Chapman SB, Dash AP, Anvikar AR, Sutton PL, Birren BW, Escalante AA, Barnwell JW, Carlton JM (2012). The malaria parasite *Plasmodium vivax* exhibits greater genetic diversity than *Plasmodium falciparum*. Nat Genet.

[33] Suyama M, Torrents D, Bork P (2006). PAL2NAL: robust conversion of protein sequence alignments into the corresponding codon alignments. Nucleic Acids Res.

[34] Jorda J, Kajava AV (2009). T-REKS: identification of Tandem REpeats in sequences with a K-meanS based algorithm. Bioinformatics.

[35] Librado P, Rozas J (2009). DnaSP v5: a software for comprehensive analysis of DNA polymorphism data. Bioinformatics.

[36] Depaulis F, Veuille M (1998). Neutrality tests based on the distribution of haplotypes under an infinite-site model. Mol Biol Evol.

[37] Zhang J, Rosenberg HF, Nei M (1998). Positive Darwinian selection after gene duplication in primate ribonuclease genes. Proc Natl Acad Sci U S A.

[38] Nei M, Kumar S (2000). Molecular evolution and phylogenetics.

[39] Tamura K, Peterson D, Peterson N, Stecher G, Nei M, Kumar S (2011). MEGA5: molecular evolutionary genetics analysis using maximum likelihood, evolutionary distance, and maximum parsimony methods. Mol Biol Evol.

[40] Delport W, Poon AF, Frost SD, Kosakovsky Pond SL (2010). Datamonkey 2010: a suite of phylogenetic analysis tools for evolutionary biology. Bioinformatics.

[41] Pond SL, Frost SD, Grossman Z, Gravenor MB, Richman DD, Brown AJ (2006). Adaptation to different human populations by HIV-1 revealed by codon-based analyses. PLoS Comput Biol.

[42] Kosakovsky Pond SL, Frost SD (2005). Not so different after all: a comparison of methods for detecting amino acid sites under selection. Mol Biol Evol.

[43] Murrell B, Wertheim JO, Moola S, Weighill T, Scheffler K, Kosakovsky Pond SL (2012). Detecting individual sites subject to episodic diversifying selection. PLoS Genet.

[44] Murrell B, Moola S, Mabona A, Weighill T, Sheward D, Kosakovsky Pond SL, Scheffler K (2013). FUBAR: a fast, unconstrained bayesian approximation for inferring selection. Mol Biol Evol.

[45] Jukes TH, Cantor CR, Munro HN (1969). Evolution of protein molecules. Mammalian Protein Metabolism.

[46] Kelly JK (1997). A test of neutrality based on interlocus associations. Genetics.

[47] Kosakovsky Pond SL, Posada D, Gravenor MB, Woelk CH, Frost SD (2006). Automated phylogenetic detection of recombination using a genetic algorithm. Mol Biol Evol.

[48] Rozas J, Gullaud M, Blandin G, Aguade M (2001). DNA variation at the rp49 gene region of *Drosophila simulans*: evolutionary inferences from an unusual haplotype structure. Genetics.

[49] Hudson RR, Kaplan NL (1985). Statistical properties of the number of recombination events in the history of a sample of DNA sequences. Genetics.

[50] Martin DP, Lemey P, Lott M, Moulton V, Posada D, Lefeuvre P (2010). RDP3: a flexible and fast computer program for analyzing recombination. Bioinformatics.

[51] Tachibana S, Sullivan SA, Kawai S, Nakamura S, Kim HR, Goto N, Arisue N, Palacpac NM, Honma H, Yagi M, Tougan T, Katakai Y, Kaneko O, Mita T, Kita K, Yasutomi Y, Sutton PL, Shakhbatyan R, Horii T, Yasunaga T, Barnwell JW, Escalante AA, Carlton JM, Tanabe K (2012). *Plasmodium cynomolgi* genome sequences provide insight into *Plasmodium vivax* and the monkey malaria clade. Nat Genet.

[52] Mongui A, Perez-Leal O, Soto SC, Cortes J, Patarroyo MA (2006). Cloning, expression, and characterisation of a *Plasmodium vivax* MSP7 family merozoite surface protein. Biochem Biophys Res Commun.

[53] Figtree M, Pasay CJ, Slade R, Cheng Q, Cloonan N, Walker J, Saul A (2000). *Plasmodium vivax* synonymous substitution frequencies, evolution and population structure deduced from diversity in AMA 1 and MSP 1 genes. Mol Biochem Parasitol.

[54] Mascorro CN, Zhao K, Khuntirat B, Sattabongkot J, Yan G, Escalante AA, Cui L (2005). Molecular evolution and intragenic recombination of the merozoite surface protein MSP-3alpha from the malaria parasite *Plasmodium vivax* in Thailand. Parasitology.

[55] Gomez A, Suarez CF, Martinez P, Saravia C, Patarroyo MA (2006). High polymorphism in *Plasmodium vivax* merozoite surface protein-5 (MSP5). Parasitology.

[56] Putaporntip C, Udomsangpetch R, Pattanawong U, Cui L, Jongwutiwes S (2010). Genetic diversity of the *Plasmodium vivax* merozoite surface protein-5 locus from diverse geographic origins. Gene.

[57] Nurminsky D (2005). Selective sweep.

[58] Putaporntip C, Jongwutiwes S, Ferreira MU, Kanbara H, Udomsangpetch R, Cui L (2009). Limited global diversity of the *Plasmodium vivax* merozoite surface protein 4 gene. Infect Genet Evol.

[59] Martinez P, Suarez CF, Gomez A, Cardenas PP, Guerrero JE, Patarroyo MA (2005). High level of conservation in Plasmodium vivax merozoite surface protein 4 (PvMSP4). Infect Genet Evol.

[60] Pacheco MA, Elango AP, Rahman AA, Fisher D, Collins WE, Barnwell JW, Escalante AA (2012). Evidence of purifying selection on merozoite surface protein 8 (MSP8) and 10 (MSP10) in Plasmodium spp. Infect Genet Evol.

[61] Forero-Rodriguez J, Garzon-Ospina D, Patarroyo MA (2014). Low genetic diversity and functional constraint in loci encoding *Plasmodium vivax* P12 and P38 proteins in the Colombian population. Malar J.

[62] Forero-Rodriguez J, Garzon-Ospina D, Patarroyo MA (2014). Low genetic diversity in the locus encoding the *Plasmodium vivax* P41 protein in Colombia’s parasite population. Malar J.

[63] Chenet SM, Pacheco MA, Bacon DJ, Collins WE, Barnwell JW, Escalante AA (2013). The evolution and diversity of a low complexity vaccine candidate, merozoite surface protein 9 (MSP-9), in *Plasmodium vivax* and closely related species. Infect Genet Evol.

[64] Pacheco MA, Ryan EM, Poe AC, Basco L, Udhayakumar V, Collins WE, Escalante AA (2010). Evidence for negative selection on the gene encoding rhoptry-associated protein 1 (RAP-1) in Plasmodium spp. Infect Genet Evol.

[65] Carlton JM, Das A, Escalante AA (2013). Genomics, population genetics and evolutionary history of *Plasmodium vivax*. Adv Parasitol.

[66] Slatkin M, Wiehe T (1998). Genetic hitch-hiking in a subdivided population. Genet Res.

[67] Nurminsky DI (2001). Genes in sweeping competition. Cell Mol Life Sci.

[68] Perez-Tris J, Hellgren O, Krizanauskiene A, Waldenstrom J, Secondi J, Bonneaud C, Fjeldsa J, Hasselquist D, Bensch S (2007). Within-host speciation of malaria parasites. PLoS One.

[69] Sutherland CJ, Tanomsing N, Nolder D, Oguike M, Jennison C, Pukrittayakamee S, Dolecek C, Hien TT, do Rosario VE, Arez AP, Pinto J, Michon P, Escalante AA, Nosten F, Burke M, Lee R, Blaze M, Otto TD, Barnwell JW, Pain A, Williams J, White NJ, Day NP, Snounou G, Lockhart PJ, Chiodini PL, Imwong M, Polley SD (2010). Two nonrecombining sympatric forms of the human malaria parasite *Plasmodium ovale* occur globally. J Infect Dis.

[70] Mu J, Joy DA, Duan J, Huang Y, Carlton J, Walker J, Barnwell J, Beerli P, Charleston MA, Pybus OG, Su XZ (2005). Host switch leads to emergence of *Plasmodium vivax* malaria in humans. Mol Biol Evol.

[71] Burge C, Karlin S (1997). Prediction of complete gene structures in human genomic DNA. J Mol Biol.

[72] Rice BL, Acosta MM, Pacheco MA, Carlton JM, Barnwell JW, Escalante AA (2014). The origin and diversification of the merozoite surface protein 3 (msp3) multi-gene family in *Plasmodium vivax* and related parasites. Mol Phylogenet Evol.

