# Supplementary material for: Heterogeneous genetic diversity pattern in Plasmodium vivax genes encoding merozoite surface proteins (MSP) -7E, −7F and -7L
Source: Malar J. 2014 Dec 13;13:495. doi: 10.1186/1475-2875-13-495 (PMC4300842; doi:10.1186/1475-2875-13-495)
Supplement: Supplementary file 2 — Additional file 2: Genetic distance between pcmsp-7B , pcmsp-7E and pvmsp-7B , pvmsp-7E sequences from 5 P. vivax isolates. The number of nucleotide differences per site was estimated as well as the standard error regarding the pvmsp-7E and pvmsp-7B reference sequences and the pcmsp-7E and pcmsp-7B sequences. (PDF 186 KB) [file 12936_2014_3635_MOESM2_ESM.pdf]

Heterogeneous genetic diversity pattern in *Plasmodium vivax* genes encoding merozoite surface proteins (MSP) -7E, -7F and -7L

Additional file 2 Genetic distance between *pcmsp-7B*, *pcmsp-7E* and *pvmsp-7B*, *pvmsp-7E* sequences from 5 *P. vivax* isolates.

Sequence 1	Sequence 2	Dist	Std. Err	Sequence 2	Dist	Std. Err
pcmsp-7B	*pcmsp-7E*	0.202	0.011	-	-	-
	Sal-IB	0.216	0.011	Sal-IE	0.282	0.014
	Brazil-IB	0.216	0.011	Brazil-IE	0.284	0.014
	India-VIIB	0.216	0.011	India-VIIE	0.279	0.013
	Mauritania-IB	0.212	0.011	Mauritania-IE	0.281	0.014
	North KoreanB	0.212	0.012	North KoreanE	0.296	0.014
pcmsp-7E	Sal-IB	0.292	0.013	Sal-IE	0.207	0.013
	Brazil-IB	0.292	0.013	Brazil-IE	0.214	0.013
	India-VIIB	0.300	0.013	India-VIIE	0.206	0.013
	Mauritania-IB	0.285	0.014	Mauritania-IE	0.220	0.012
	North KoreanB	0.307	0.015	North KoreanE	0.219	0.013
Sal-IB	Sal-IE	0.211	0.011	-	-	-

The number of nucleotide differences per site was estimated as well as the standard error regarding the *pvmsp-7E* and *pvmsp-7B* reference sequences and the *pcmsp-7E* and *pcmsp-7B* sequences.
