# Supplementary material for: Heterogeneous genetic diversity pattern in Plasmodium vivax genes encoding merozoite surface proteins (MSP) -7E, −7F and -7L
Source: Malar J. 2014 Dec 13;13:495. doi: 10.1186/1475-2875-13-495 (PMC4300842; doi:10.1186/1475-2875-13-495)
Supplement: Supplementary file 3 — Additional file 3: DNA polymorphism measurements at the 5′-end, central region and 3′-end for pvmsp - 7 genes in the Colombian population. Ss: number of segregating sites, S: number of singleton sites, Ps: number of parsimony-informative sites, H: number of haplotypes, θW: Watterson estimator, π: nucleotide diversity. (SD): standard deviation. 5′-end (pvmsp-7E: nucleotide 1–390, pvmsp-7F: nucleotide 1–432, pvmsp-7L: nucleotide 1–381), central (pvmsp-7E: nucleotide 391–747, pvmsp-7F: nucleotide 433–1,053, pvmsp-7L: nucleotide 382–816) and 3′-end (pvmsp-7E: nucleotide 748–1,158, pvmsp-7F: nucleotide 1,054–1,449, pvmsp-7L: nucleotide 817–1,275). Numbers based on Additional files 4, 9 and 10. (PDF 212 KB) [file 12936_2014_3635_MOESM3_ESM.pdf]

Heterogeneous genetic diversity pattern in *Plasmodium vivax* genes encoding merozoite surface proteins (MSP) -7E, -7F and -7L

Additional file 3 DNA polymorphism measurements at the 5'-end, central region and 3'-end for *pvmsp-7s* genes in the Colombian population.

n	Gene	Sites	Ss	S	Ps	H	θ^w (se)	π (se)
				**5'-end**				
31	***msp-7E***	338	21	0	21	5	0.0155 (0.0034)	0.0188 (0.0051)
36	***msp-7F***	360	0	0	0	1	0.000	0.000
31	***msp-7L***	348	0	0	0	1	0.000	0.000
				**Central**				
31	***msp-7E***	313	98	0	98	11	0.0760 (0.0077)	0.1309 (0.0072)
36	***msp-7F***	429	1	0	1	2	0.0006 (0.0006)	0.0008 (0.0002)
31	***msp-7L***	423	2	0	2	3	0.0012 (0.0008)	0.0008 (0.0002)
				**3'-end**				
31	***msp-7E***	393	45	6	39	14	0.0286 (0.0043)	0.0295 (0.0052)
36	***msp-7F***	387	1	0	1	2	0.0006 (0.0006)	0.0012 (0.0006)
31	***msp-7L***	444	2	0	2	3	0.0011 (0.0008)	0.0009 (0.0002)

Ss: number of segregating sites, S: number of singleton sites, Ps: number of parsimony-informative sites, H: number of haplotypes, θ^W: Watterson estimator, π: nucleotide diversity. (sd): standard deviation. 5'-end (*pvmsp-7E*: nucleotide 1–390, *pvmsp-7F*: nucleotide 1–432, *pvmsp-7L*: nucleotide 1–381), central (*pvmsp-7E*: nucleotide 391–747, *pvmsp-7F*: nucleotide 433–1,053, *pvmsp-7L*: nucleotide 382–816) and 3'-end (*pvmsp-7E*: nucleotide 748–1,158, *pvmsp-7F*: nucleotide 1,054–1,449, *pvmsp-7L*: nucleotide 817–1,275). Numbers based on Additional files 4, 9 and 10.
