# Supplementary material for: Heterogeneous genetic diversity pattern in Plasmodium vivax genes encoding merozoite surface proteins (MSP) -7E, −7F and -7L
Source: Malar J. 2014 Dec 13;13:495. doi: 10.1186/1475-2875-13-495 (PMC4300842; doi:10.1186/1475-2875-13-495)
Supplement: Supplementary file 4 — Additional file 4: pvmsp-7E gene alignment. The alignment shows the 23 haplotypes found in pvmsp-7E together with the pcmsp-7E haplotype. Haplotype 1, Sal-I; haplotype 2, Brazil-I; haplotype 3, India-VII; haplotype 4, Mauritania-I; haplotype 5–23, Colombian isolates. Dots represent nucleotide identity. Codons under positive selection are shown in green (intra-species) and turquoise (inter-species) and those under negative selection are shown in yellow (intra-species) and fuchsia (inter-species). (PDF 162 KB) [file 12936_2014_3635_MOESM4_ESM.pdf]

Additional file 4 *pvmsp-7E* gene alignment.

```
               1                                                                                                                                                            120
#Haplotype_1   ATG AAA GGA GTC ACT GGG CCG ATC TGT TGT CTT TTC CTG TTT CTG TTC AGC TGC GCG TCG TCG GAA AAG TTG GGC GTC CAG AAA AAG AAA AAA AAT CTG GAG CAG GAC GCG ACG CAC GCG
#Haplotype_2   ... ... ... ... ... ... ... ... ... ... ... ... ... ... ... ... ... ... ... ... ... ... ... ... ... ... ... ... ... ... ... ... ... ... ... ... ... ... ... ...
#Haplotype_3   ... ... ... ... ... ... ... ... ... ... ... ... ... ... ... ... ... ... ... ... ... ... ... ... ... ... ... ... ... ... ... ... ... ... ... ... ... ... ... ...
#Haplotype_4   ... ... ... ... ... ... ... ... ... ... ... ..G ... C.. ... ... T.. ... ... ... ... ... ... ... ... ... ... ... .G. ... ... ... ... ... ... ... ... ... ... ...
#Haplotype_5   --- --- --- --- --- --- --- --- --- --- --- --- --- --- --- --- --- ... ... ... ... ... ... ... ... ... ... ... ... ... ... ... ... ... ... ... ... ... ... ...
#Haplotype_6   --- --- --- --- --- --- --- --- --- --- --- --- --- --- --- --- --- ... ... ... ... ... ... ... ... ... ... ... ... ... ... ... ... ... ... ... ... ... ... ...
#Haplotype_7   --- --- --- --- --- --- --- --- --- --- --- --- --- --- --- --- --- ... ... ... ... ... ... ... ... ... ... ... ... ... ... ... ... ... ... ... ... ... ... ...
#Haplotype_8   --- --- --- --- --- --- --- --- --- --- --- --- --- --- --- --- --- ... ... ... ... ... ... ... ... ... ... ... .G. ... ... ... ... ... ... ... ... ... ... ...
#Haplotype_9   --- --- --- --- --- --- --- --- --- --- --- --- --- --- --- --- --- ... ... ... ... ... ... ... ... ... ... ... ... ... ... ... ... ... ... ... ... ... ... ...
#Haplotype_10  --- --- --- --- --- --- --- --- --- --- --- --- --- --- --- --- --- ... ... ... ... ... ... ... ... ... ... ... ... ... ... ... ... ... ... ... ... ... ... ...
#Haplotype_11  --- --- --- --- --- --- --- --- --- --- --- --- --- --- --- --- --- ... ... ... ... ... ... ... ... ... ... ... ... ... ... ... ... ... ... ... ... ... ... ...
#Haplotype_12  --- --- --- --- --- --- --- --- --- --- --- --- --- --- --- --- --- ... ... ... ... ... ... ... ... ... ... ... .G. ... ... ... ... ... ... ... ... ... ... ...
#Haplotype_13  --- --- --- --- --- --- --- --- --- --- --- --- --- --- --- --- --- ... ... ... ... ... ... ... ... ... ... ... .G. ... ... ... ... ... ... ... ... ... ... ...
#Haplotype_14  --- --- --- --- --- --- --- --- --- --- --- --- --- --- --- --- --- ... ... ... ... ... ... ... ... ... ... ... .G. ... ... ... ... ... ... ... ... ... ... ...
#Haplotype_15  --- --- --- --- --- --- --- --- --- --- --- --- --- --- --- --- --- ... ... ... ... ... ... ... ... ... ... ... ... ... ... ... ... ... ... ... ... ... ... ...
#Haplotype_16  --- --- --- --- --- --- --- --- --- --- --- --- --- --- --- --- --- ... ... ... ... ... ... ... ... ... ... ... ... ... ... ... ... ... ... ... ... ... ... ...
#Haplotype_17  --- --- --- --- --- --- --- --- --- --- --- --- --- --- --- --- --- ... ... ... ... ... ... ... ... ... ... ... .G. ... ... ... ... ... ... ... ... ... ... ...
#Haplotype_18  --- --- --- --- --- --- --- --- --- --- --- --- --- --- --- --- --- ... ... ... ... ... ... ... ... ... ... ... ... ... ... ... ... ... ... ... ... ... ... ...
#Haplotype_19  --- --- --- --- --- --- --- --- --- --- --- --- --- --- --- --- --- ... ... ... ... ... ... ... ... ... ... ... ... ... ... ... ... ... ... ... ... ... ... ...
#Haplotype_20  --- --- --- --- --- --- --- --- --- --- --- --- --- --- --- --- --- ... ... ... ... ... ... ... ... ... ... ... ... ... ... ... ... ... ... ... ... ... ... ...
#Haplotype_21  --- --- --- --- --- --- --- --- --- --- --- --- --- --- --- --- --- ... ... ... ... ... ... ... ... ... ... ... ... ... ... ... ... ... ... ... ... ... ... ...
#Haplotype_22  --- --- --- --- --- --- --- --- --- --- --- --- --- --- --- --- --- ... ... ... ... ... ... ... ... ... ... ... ... ... ... ... ... ... ... ... ... ... ... ...
#Haplotype_23  --- --- --- --- --- --- --- --- --- --- --- --- --- --- --- --- --- ... ... ... ... ... ... ... ... ... ... ... ... ... ... ... ... ... ... ... ... ... ... ...
#P_cynomolgi   ... .G. A.. A.. .T. ... ... ... ... ... ... ..G ... ... ... ... T.. ... ... ... ... ... ... ... ... AC. ..T ..G ... ... ... --- T.. ... ... ..A ... .T. ..T ...

               121                                                                                                                                                          240
#Haplotype_1   TTG ATG AAG AAG TTG GAA AGT CTG TAC AAA CTC AGT GCA ACG GAC AAC AGC GAA ATT TTT AAC AAA GAA ATT GAG TCG TTG AAA AAG CAG ATT GAT CAG TTG CAC CAA CAT GGT GGG GAA
#Haplotype_2   ... ... ... ... ... ... ... ... ... ... ... ... ... ... ... ... ... ... ... ... ... ... ... ... ... ... ... ... ... ... ... ... ... ... ... ... ... ... ... ...
#Haplotype_3   ... ... ... ... ... ... ... ... ... ... ... ... ... ... ... ... ... ... ... ... ... ... ... ... ... ... ... ... ... ... ... ... ... ... ... ... ... ... ... ...
#Haplotype_4   ... ... ... ... ... ..G ... T.. ... ... ..G ..C ..G ... ... ..T ... ... ... ... ... ... ..G ..C ... ... ... ... ... ... ... ..C ... ... ... ... ... ..G ... ...
#Haplotype_5   ... ... ... ... ... ... ... ... ... ... ... ... ... ... ... ... ... ... ... ... ... ... ... ... ... ... ... ... ... ... ... ... ... ... ... ... ... ... ... ...
#Haplotype_6   ... ... ... ... ... ... ... ... ... ... ... ... ... ... ... ... ... ... ... ... ... ... ... ... ... ... ... ... ... ... ... ... ... ... ... ... ... ... ... ...
#Haplotype_7   ... ... ... ... ... ... ... ... ... ... ... ... ... ... ... ... ... ... ... ... ... ... ... ... ... ... ... ... ... ... ... ... ... ... ... ... ... ... ... ...
#Haplotype_8   ... ... ... ... ... ..G ... ... ... ... ..G ..C ..G ... ... ... ... ... ... ... ... ... ..G ..C ... ... ... ... ... ... ... ..C ... ... ... ... ... ..G ... ...
#Haplotype_9   ... ... ... ... ... ... ... ... ... ... ... ... ... ... ... ... ... ... ... ... ... ... ... ... ... ... ... ... ... ... ... ... ... ... ... ... ... ... ... ...
#Haplotype_10  ... ... ... ... ... ... ... ... ... ... ... ... ... ... ... ... ... ... ... ... ... ... ... ... ... ... ... ... ... ... ... ... ... ... ... ... ... ... ... ...
#Haplotype_11  ... ... ... ... ... ... ... ... ... ... ... ... ... ... ... ... ... ... ... ... ... ... ... ... ... ... ... ... ... ... ... ... ... ... ... ... ... ... ... ...
#Haplotype_12  ... ... ... ... ... ..G ... T.. ... ... ..G ..C ..G ... ... ..T ... ... ... ... ... ... ..G ..C ... ... ... ... ... ... ... ..C ... ... ... ... ... ..G ... ...
#Haplotype_13  ... ... ... ... ... ..G ... T.. ... ... ..G ..C ..G ... ... ..T ... ... ... ... ... ... ..G ..C ... ... ... ... ... ... ... ..C ... ... ... ... ... ..G ... ...
#Haplotype_14  ... ... ... ... ... ..G ... T.. ... ... ..G ..C ..G ... ... ..T ... ... ... ... ... ... ..G ..C ... ... ... ... ... ... ... ..C ... ... ... ... ... ..G ... ...
#Haplotype_15  ... ... ... ... ... ... ... ... ... ... ... ... ... ... ... ... ... ... ... ... ... ... ... ... ... ... ... ... ... ... ... ... ... ... ... ... ... ... ... ...
#Haplotype_16  ... ... ... ... ... ... ... ... ... ... ... ... ... ... ... ... ... ... ... ... ... ... ... ... ... ... ... ... ... ... ... ... ... ... ... ... ... ... ... ...
#Haplotype_17  ... ... ... ... ... ..G ... T.. ... ... ..G ..C ..G ... ... ..T ... ... ... ... ... ... ..G ..C ... ... ... ... ... ... ... ..C ... ... ... ... ... ..G ... ...
#Haplotype_18  ... ... ... ... ... ... ... ... ... ... ... ... ... ... ... ... ... ... ... ... ... ... ... ... ... ... ... ... ... ... ... ... ... ... ... ... ... ... ... ...
#Haplotype_19  ... ... ... ... ... ... ... ... ... ... ... ... ... ... ... ... ... ... ... ... ... ... ... ... ... ... ... ... ... ... ... ... ... ... ... ... ... ... ... ...
#Haplotype_20  ... ... ... ... ... ... ... ... ... ... ... ... ... ... ... ... ... ... ... ... ... ... ... ... ... ... ... ... ... ... ... ... ... ... ... ... ... ... ... ...
#Haplotype_21  ... ... ... ... ... ... ... ... ... ... ... ... ... ... ... ... ... ... ... ... ... ... ... ... ... ... ... ... ... ... ... ... ... ... ... ... ... ... ... ...
#Haplotype_22  ... ... ... ... ... ... ... ... ... ... ... ... ... ... ... ... ... ... ... ... ... ... ... ... ... ... ... ... ... ... ... ... ... ... ... ... ... ... ... ...
#Haplotype_23  ... ... ... ... ... ... ... ... ... ... ... ... ... ... ... ... ... ... ... ... ... ... ... ... ... ... ... ... ... ... ... ... ... ... ... ... ... ... ... ...
#P_cynomolgi   ... ... ... ... C.. ... .A. ... ..T .G. ... ..C ..G ... A.T ... ... ... G.. ..G ... ... ... ... ... ... ... ... ..A ... ... ..C ... ... ... ... ... ... ..A .T.
```

```
               241                                                                                                                                                        360
#Haplotype_1   AAC GAA GGG GAG AGT TTA GGC CAC CTT CTA GAA AGC GAG GCC GCC AAT GAG TCA GCC AAA AAG ACC ATT TTT GGA GTG GAC GAA GAC GAT TTA GAT AAC TAC GAC GCG GAC TTC ATT GGA
#Haplotype_2   ... ... ... ... ... ... ... ... ... ... ... ... ... ... ... ... ... ... ... ... ... ... ... ... ... ... ... ... ... ... ... ... ... ... ... ... ... ... ... ...
#Haplotype_3   ... ... ... ... ... ... ... ... ... ... ... ... ... ... ... ... ... ... ... ... ... ... ... ... ... ... ... ... ... ... ... ... ... ... ... ... ... ... ... ...
#Haplotype_4   ... ... .A. ... ... ... ... ... ... A.. ... ... ... ... ... ... ... ... ... ..G ... ... ... ... ... ... ... ... ... ... .C. ... ... ... ... ... ... ... ..C ..G
#Haplotype_5   ... ... ... ... ... ... ... ... ... ... ... ... ... ... ... ... ... ... ... ... ... ... ... ... ... ... ... ... ... ... ... ... ... ... ... ... ... ... ... ...
#Haplotype_6   ... ... ... ... ... ... ... ... ... ... ... ... ... ... ... ... ... ... ... ... ... ... ... ... ... ... ... ... ... ... ... ... ... ... ... ... ... ... ... ...
#Haplotype_7   ... ... ... ... ... ... ... ... ... ... ... ... ... ... ... ... ... ... ... ... ... ... ... ... ... ... ... ... ... ... ... ... ... ... ... ... ... ... ... ...
#Haplotype_8   ... ... .A. ... ... ... ... ... ... A.. ... ... ... ... ... ... ... ... ... ... ... ... ... ... ... ... ... ... ... ... .C. ... ... ... ... ... ... ... ..C ..G
#Haplotype_9   ... ... ... ... ... ... ... ... ... ... ... ... ... ... ... ... ... ... ... ... ... ... ... ... ... ... ... ... ... ... ... ... ... ... ... ... ... ... ... ...
#Haplotype_10  ... ... ... ... ... ... ... ... ... ... ... ... ... ... ... ... ... ... ... ... ... ... ... ... ... ... ... ... ... ... ... ... ... ... ... ... ... ... ... ...
#Haplotype_11  ... ... ... ... ... ... ... ... ... ... ... ... ... ... ... ... ... ... ... ... ... ... ... ... ... ... ... ... ... ... ... ... ... ... ... ... ... ... ... ...
#Haplotype_12  ... ... .A. ... ... ... ... ... ... A.. ... ... ... ... ... ... ... ... ... ..G ... ... ... ... ... ... ... ... ... ... .C. ... ... ... ... ... ... ... ..C ..G
#Haplotype_13  ... ... .A. ... ... ... ... ... ... A.. ... ... ... ... ... ... ... ... ... ..G ... ... ... ... ... ... ... ... ... ... .C. ... ... ... ... ... ... ... ..C ..G
#Haplotype_14  ... ... .A. ... ... ... ... ... ... A.. ... ... ... ... ... ... ... ... ... ..G ... ... ... ... ... ... ... ... ... ... .C. ... ... ... ... ... ... ... ..C ..G
#Haplotype_15  ... ... ... ... ... ... ... ... ... ... ... ... ... ... ... ... ... ... ... ... ... ... ... ... ... ... ... ... ... ... ... ... ... ... ... ... ... ... ... ...
#Haplotype_16  ... ... ... ... ... ... ... ... ... ... ... ... ... ... ... ... ... ... ... ... ... ... ... ... ... ... ... ... ... ... ... ... ... ... ... ... ... ... ... ...
#Haplotype_17  ... ... .A. ... ... ... ... ... ... A.. ... ... ... ... ... ... ... ... ... ..G ... ... ... ... ... ... ... ... ... ... ... ... ... ... ... ... ... ... ... ...
#Haplotype_18  ... ... ... ... ... ... ... ... ... ... ... ... ... ... ... ... ... ... ... ... ... ... ... ... ... ... ... ... ... ... ... ... ... ... ... ... ... ... ... ...
#Haplotype_19  ... ... ... ... ... ... ... ... ... ... ... ... ... ... ... ... ... ... ... ... ... ... ... ... ... ... ... ... ... ... ... ... ... ... ... ... ... ... ... ...
#Haplotype_20  ... ... ... ... ... ... ... ... ... ... ... ... ... ... ... ... ... ... ... ... ... ... ... ... ... ... ... ... ... ... ... ... ... ... ... ... ... ... ... ...
#Haplotype_21  ... ... ... ... ... ... ... ... ... ... ... ... ... ... ... ... ... ... ... ... ... ... ... ... ... ... ... ... ... ... ... ... ... ... ... ... ... ... ... ...
#Haplotype_22  ... ... ... ... ... ... ... ... ... ... ... ... ... ... ... ... ... ... ... ... ... ... ... ... ... ... ... ... ... ... ... ... ... ... ... ... ... ... ... ...
#Haplotype_23  ... ... ... ... ... ... ... ... ... ... ... ... ... ... ... ... ... ... ... ... ... ... ... ... ... ... ... ... ... ... ... ... ... ... ... ... ... ... ... ...
#P_cynomolgi   .T. ... ... ..A .A. C.. ... ..T ... ... ... ... ..A ..A ... ..C ... ... A.. ..G ... ... ... ..C ..C A.. ... ... ..T ..C C.G ... ... ... ..T .T. ... ..T .CA ...

               361                                                                                                                                                        480
#Haplotype_1   CAG AGC AAA GGC AAA ATT AAG GGG CAA GCG GAT ACA GAC AAC CAA GCT CAG AGA --- ACT GCT GAC GTG GCG GCC CAA CCC GGG GGT GTA TCA CCT TCT ACG AGT GCC CGA CCA CAG GAA
#Haplotype_2   ... ... ... ... ... ... ... ... ... ... ... ... ... ... ... ... ... ... --- ... ... ... ... ... ... ... ... ... ... ... ... ... ... G.. G.G ..A .A. T.. .G. ..C
#Haplotype_3   ... ... ... ... ... ... ... ... ... A.. ..G GG. .G. G.T .GC A.. ... --- AGC C.. ... ... ... ... ..G .C. G.. A.. ..A ... ... ... ... ... ... ... ... ... ... ...
#Haplotype_4   ... ... ... A.A ... ... ..A ... ... ... .T. G.. ... ..T G.. ... ... ... GCT C.. .AC A.. T.. C.. ..G .C. .AA ... C.. .A. .T. T.. G.. G.. ... .GG .A. ... ... ...
#Haplotype_5   ... ... ... ... ... ... ... ... ... ... ... ... ... ... ... ... ... ... --- ... ... ... ... ... ... ... ... ... ... ... ... ... ... ... ... ... ... ... ... ...
#Haplotype_6   ... ... ... ... ... ... ... ... ... ... ... ... ... ... ... ... ... ... --- ... ... ... ... ... ... ... ... ... ... ... ... ... ... ... ... ... ... ... ... ...
#Haplotype_7   ... ... ... ... ... ... ... ... ... ... ... ... ... ... ... ... ... ... --- ... ... ... ... ... ... ... ... ... ... ... ... ... ... G.. G.G ..A .A. T.. .G. ..C
#Haplotype_8   ... ... ... A.A ... ... ..A ... ... ... .T. G.. ... ..T G.. ... ... ... GCT C.. .AC A.. T.. C.. ..G .C. .AA ... C.. .A. .T. T.. G.. G.. ... .GG .A. ... ... ...
#Haplotype_9   ... ... ... ... ... ... ... ... ... A.. ..G GG. .G. G.T .GC A.. ... --- AGC C.. ... ... ... ... ..G .C. G.. A.. ..A ... ... ... ... ... ... ... ... ... ... ...
#Haplotype_10  ... ... ... ... ... ... ... ... ... ... ... ... ... ... ... ... ... ... --- ... ... ... ... ... ... ... ... ... ... ... .T. ... ... G.. G.G ..A .A. T.. .G. ..C
#Haplotype_11  ... ... ... ... ... ... ... ... ... ... ... ... ... ... ... ... ... ... --- ... ... ... ... ... ... ... ... ... ... ... .T. ... ... G.. G.G ..A .A. T.. .G. ..C
#Haplotype_12  ... ... ... A.A ... ... ..A ... ... ... .T. G.. ... ..T G.. ... ... ... GCT C.. .AC A.. T.. C.. ..G .C. .AA ... C.C .A. .T. T.. G.. G.. ... .GG .A. ... ... ...
#Haplotype_13  ... ... ... A.A ... ... ..A ... ... ... .T. G.. ... ..T G.. ... ... ... GCT C.. .AC A.. T.. C.. ..G .C. .AA ... C.. .A. .T. T.. G.. G.. ... .GG .A. ... ... ...
#Haplotype_14  ... ... ... A.A ... ... ..A ... ... ... .T. G.. ... ..T G.. ... ... ... GCT C.. .AC A.. T.. C.. ..G .C. .AA ... C.. .A. .T. T.. G.. G.. ... .GG .A. ... ... ...
#Haplotype_15  ... ... ... ... ... ... ... ... ... ... ... ... ... ... ... ... ... ... --- ... ... ... ... ... ... ... ... ... ... ... .T. ... ... G.. G.G ..A .A. T.. .G. ..C
#Haplotype_16  ... ... ... ... ... ... ... ... ... ... ... ... ... ... ... ... ... ... --- ... ... ... ... ... ... ... ... ... ... ... .T. ... ... G.. G.G ..A .A. T.. .G. ..C
#Haplotype_17  ... ... ... ... ... ... ... ... ... A.. ..G GG. .G. G.T .GC A.. ... --- AGC C.. ... ... ... ... ..G .C. .AA ... C.. .A. .T. T.. G.. G.. ... .GG .A. ... ... ...
#Haplotype_18  ... ... ... ... ... ... ... ... ... ... ... ... ... ... ... ... ... ... --- ... ... ... ... ... ... ... ... ... ... ... ... ... ... G.. G.G ..A .A. T.. .G. ..C
#Haplotype_19  ... ... ... ... ... ... ... ... ... A.. ..G GG. .G. G.T .GC A.. ... --- AGC C.. ... ... ... ... ..G .C. G.. A.. ..A ... ... ... ... ... ... ... ... ... ... ...
#Haplotype_20  ... ... ... ... ... ... ... ... ... ... ... ... ... ... ... ... ... ... --- ... ... ... ... ... ... ... ... ... ... ... .T. ... ... G.. G.G ..A .A. T.. .G. ..C
#Haplotype_21  ... ... ... ... ... ... ... ... ... ... ... ... ... ... ... ... ... ... --- ... ... ... ... ... ... ... ... ... ... ... ... ... ... G.. G.G ..A .A. T.. .G. ..C
#Haplotype_22  ... ... ... ... ... ... ... ... ... ... ... ... ... ... ... ... ... ... --- ... ... ... ... ... ... ... ... ... ... ... ... ... ... G.. G.G ..A .A. T.. .G. ..C
#Haplotype_23  ... ... ... ... ... ... ... ... ... ... ... ... ... ... ... ... ... ... --- ... ... ... ... ... ... ... ... ... ... ... ... ... ... ... ... ... ... ... ... ...
#P_cynomolgi   ... G.. ... ..A ... ... ... ... ... ..A ... ... .G. C.G GGC ... ... GA. ACT ... .GC A.. T.. ... ..G ... G.. ... ... .A. G.. T.. GA. .G. G.. .G. .A. .A. ... C..
```

```
              481                                                                                                                                                          600
#Haplotype_1  CCA GGA AAA ACC GGA GTG ACA GGT AGT CCA AAT GGC TTA GTA GAA GCA GGT TTG GTA AAC ACC AAA ACA CTT CAA AAT GTA --- --- --- --- --- --- GGA CCT AAT GGG CAA CGA GCT
#Haplotype_2  A.. .C. .GG C.. .A. .C. ... .A. ..A ... ... ... G.. ... ... AG. ... ..C ... G.. ... .G. ... ... ... ... ... --- --- --- --- --- --- ... GA. ... ... ... .C. A..
#Haplotype_3  ... ... ... ... ... ... ... ... ... ... ... ... ... ... ... ... ... ... ... ... ... ... ... ... ... ... ... --- --- --- --- --- --- ... ... ... ... ... ... ...
#Haplotype_4  T.. .C. .G. C.. CA. ... ... ... ... ... GGG A.T CA. A.. ... .G. ... ..C ... ... ... .G. ... ... G.. ... ... --- --- --- --- --- --- .AG G.. ... ... ... ... .T.
#Haplotype_5  ... ... ... ... ... ... ... ... ... ... ... ... ... ... ... ... ... ... ... ... ... ... ... ... ... ... ... --- --- --- --- --- --- ... ... ... ... ... ... ...
#Haplotype_6  ... ... ... ... ... ... ... ... ... ... ... ... ... ... ... ... ... ... ... ... ... ... ... ... ... ... ... --- --- --- --- --- --- ... ... ... ... ... ... ...
#Haplotype_7  A.. .C. .GG C.. .A. .C. ... .A. ..A ... ... ... G.. ... ... AG. ... ..C ... G.. ... .G. ... ... ... ... ... --- --- --- --- --- --- ... GA. ... ... ... .C. A..
#Haplotype_8  T.. .C. .G. C.. CA. ... ... ... ... ... GGG A.T CA. A.. ... .G. ... ..C ... ... ... .G. ... ... G.. ... ... --- --- --- --- --- --- .AG G.. ... ... ... ... .T.
#Haplotype_9  ... ... ... ... ... ... ... ... ... ... ... ... ... ... ... ... ... .C. ... ... ... .G. ... ... G.. ... ... --- --- --- --- --- --- .AG G.. ... ... ... ... .T.
#Haplotype_10 A.. .C. .GG C.. .A. .C. ... ... ... ... ... ... ... ... ... ... ... ... ... ... ... .G. ... ... G.. ... ... --- --- --- --- --- --- .AG G.. ... ... ... ... .T.
#Haplotype_11 A.. .C. .GG C.. .A. .C. ... .A. ..A ... ... ... G.. ... ... AG. ... ..C ... G.. ... .G. ... ... ... ... ... --- --- --- --- --- --- ... GA. ... ... ... .C. A..
#Haplotype_12 T.. .C. .G. C.. CA. ... ... ... ... ... GGG A.T CA. A.. ... .G. ... ..C ... ... ... .G. ... ... G.. ... ... --- --- --- --- --- --- .AG G.. ... ... ... ... .T.
#Haplotype_13 T.. .C. .G. C.. CA. ... ... ... ... ... GGG A.T CA. A.. ... .G. ... ..C ... ... ... .G. ... ... G.. ... ... --- --- --- --- --- --- .AG G.. ... ... ... ... .T.
#Haplotype_14 T.. .C. .G. ... CA. ... ... ... ... ... GGG A.A CA. A.. ... .G. ... ..C ... ... ... .G. ... ... G.. ... ... --- --- --- --- --- --- .AG G.. ... ... ... ... .T.
#Haplotype_15 A.. .C. .GG C.. .A. .C. ... .A. ..A ... ... ... G.. ... ... AG. ... ..C ... G.. ... .G. ... ... ... ... ... --- --- --- --- --- --- ... GA. ... ... ... .C. A..
#Haplotype_16 A.. .C. .GG C.. .A. .C. ... .A. ..A ... ... ... G.. ... ... AG. ... ..C ... G.. ... .G. ... ... ... ... ... --- --- --- --- --- --- ... GA. ... ... ... .C. A..
#Haplotype_17 T.. .C. .G. C.. CA. ... ... ... ... ... GGG A.T CA. A.. ... .G. ... ..C ... ... ... .G. ... ... G.. ... ... --- --- --- --- --- --- .AG G.. ... ... ... ... .T.
#Haplotype_18 A.. .C. .GG C.. .A. .C. ... .A. ..A ... ... ... G.. ... ... AG. ... ..C ... G.. ... .G. ... ... ... ... ... --- --- --- --- --- --- ... GA. ... ... ... .C. A..
#Haplotype_19 ... ... ... ... ... ... ... ... ... ... ... ... ... ... ... ... ... .C. ... ... ... .G. ... ... G.. ... ... --- --- --- --- --- --- .AG G.. ... ... ... ... .T.
#Haplotype_20 A.. .C. .GG C.. .A. .C. ... .A. ..A ... ... ... G.. ... ... AG. ... ..C ... G.. ... .G. ... ... ... ... ... --- --- --- --- --- --- ... GA. ... ... ... .C. A..
#Haplotype_21 A.. .C. .GG C.. .A. .C. ... .A. ..A ... ... ... G.. ... ... AG. ... ..C ... G.. ... .G. ... ... ... ... ... --- --- --- --- --- --- ... GA. ... ... ... .C. A..
#Haplotype_22 A.. .C. .GG C.. .A. .C. ... .A. ..A ... ... ... G.. ... ... AG. ... ..C ... G.. ... .G. ... ... ... ... ... --- --- --- --- --- --- ... GA. ... ... ... .C. A..
#Haplotype_23 ... ... ... ... ... ... ... ... ... ... ... ... ... ... ... ... ... ... ... ... ... ... ... ... ... ... ... --- --- --- --- --- --- ... ... ... ... ... ... ...
#P_cynomolgi  ... .C. .G. C.. A.C .G. T.. .A. ... ... GGG ... G.. ... ... .G. ... ... ... ..T GT. ..T ... ... ... ... ... CCA GTT CAT GTG CAA TCC TC. AGA ... ... .C. .A. A..

              601                                                                                                                                                          720
#Haplotype_1  GCG GAT CCC CAG CCA GGG CGA GCA GCA AAT CTA CCA GAA GGG CAA CGT ACA AAT GAC CCC CAA CAA --- --- GGC --- GGA AGC GAA TCA ACG GAA GGA CCA GCC GTT ACT CCG CGA CCA
#Haplotype_2  .T. ... AGT ... T.T ... ... ... ... .C. .A. ... ... ... ... ... G.. ... .G. .A. ... ... --- GAA ... --- ... ... ... ... ... ... ... ... ... ... ... ... ... ...
#Haplotype_3  ... ... ... ... ... ... ... ... ... ... ... ... ... ... ... ... ... ... ... ... ... ... --- --- ... --- ... ... ... ... ... ... ... ... ... ... ... ... ... ...
#Haplotype_4  ... ... ..T ... T.. A.. .C. ... ... .C. .A. ... ... ... ... G.. G.. ... .G. ... ... ... --- --- ... --- .A. ..A .C. C.. ... ... A.. A.. ... ..A ... ... TC. ...
#Haplotype_5  ... ... ... ... ... ... ... ... ... ... ... ... ... ... ... ... ... ... ... ... ... ... --- --- ... --- ... ... ... ... ... ... ... ... ... ... ... ... ... ...
#Haplotype_6  ... ... ... ... ... ... ... ... ... ... ... ... ... ... ... ... ... ... ... ... ... ... --- --- ... --- ... ... ... ... ... ... ... ... ... ... ... ... ... ...
#Haplotype_7  .T. ... AGT ... T.T ... ... ... ... .C. .A. ... ... ... ... ... G.. ... .G. .A. ... ... GAA GAA ... --- ... ... ... ... ... ... ... ... ... ... ... ... ... ...
#Haplotype_8  ... ... ..T ... T.. A.. .C. ... ... .C. .A. ... ... ... ... G.. G.. ... .G. ... ... ... --- --- ... --- .A. ..A .C. C.. ... ... A.. A.. ... ..A ... ... TC. ...
#Haplotype_9  ... ... ..T ... T.. A.. .C. ... ... .C. .A. ... ... ... ... G.. G.. ... .G. ... ... ... --- --- ... --- .A. ..A .C. C.. ... ... A.. A.. ... ..A ... ... TC. ...
#Haplotype_10 ... ... ..T ... T.. A.. .C. ... ... .C. .A. ... ... ... ... G.. G.. ... .G. ... ... ... --- --- ... --- .A. ..A .C. C.. ... ... A.. A.. ... ..A ... ... TC. ...
#Haplotype_11 .T. ... AGT ... T.T ... ... ... ... .C. .A. ... ... ... ... ... G.. ... .G. .A. ... ... --- GAA ... --- ... ... ... ... ... ... ... ... ... ... ... ... ... ...
#Haplotype_12 ... ... ..T ... T.. A.. .C. ... ... .C. .A. ... ... ... ... G.. G.. ... .G. ... ... ... --- --- ... --- .A. ..A .C. C.. ... ... A.. A.. ... ..A ... ... TC. ...
#Haplotype_13 ... ... ..T ... T.. A.. .C. ... ... .C. .A. ... ... ... ... G.. G.. ... .G. ... ... ... --- --- ... --- .A. ..A .C. C.. ... ... A.. A.. ... ..A ... ... TC. ...
#Haplotype_14 ... ... ..T ... T.. A.. .C. ... ... .C. .A. ... ... ... ... G.. G.. ... .G. ... ... ... --- --- ... --- .A. ..A .C. C.. ... ... A.. A.. ... ..A ... ... TC. ...
#Haplotype_15 .T. ... AGT ... T.T ... ... ... ... .C. .A. ... ... ... ... ... G.. ... .G. .A. ... ... --- GAA ... --- ... ... ... ... ... ... ... ... ... ... ... ... ... ...
#Haplotype_16 .T. ... AGT ... T.T ... ... ... ... .C. .A. ... ... ... ... ... G.. ... .G. .A. ... ... --- GAA ... --- ... ... ... ... ... ... ... ... ... ... ... ... ... ...
#Haplotype_17 ... ... ..T ... T.. A.. .C. ... ... .C. .A. ... ... ... ... G.. G.. ... .G. ... ... ... --- --- ... --- .A. ..A .C. C.. ... ... A.. A.. ... ..A ... ... TC. ...
#Haplotype_18 .T. ... AGT ... T.T ... ... ... ... .C. .A. ... ... ... ... ... G.. ... .G. .A. ... ... --- GAA ... --- ... ... ... ... ... ... ... ... ... ... ... ... ... ...
#Haplotype_19 ... ... ..T ... T.. A.. .C. ... ... .C. .A. ... ... ... ... G.. G.. ... .G. ... ... ... --- --- ... --- .A. ..A .C. C.. ... ... A.. A.. ... ..A ... ... TC. ...
#Haplotype_20 .T. ... AGT ... T.T ... ... ... ... .C. .A. ... ... ... ... ... G.. ... .G. .A. ... ... GAA GAA ... --- ... ... ... ... ... ... ... ... ... ... ... ... ... ...
#Haplotype_21 .T. ... AGT ... T.T ... ... ... ... .C. .A. ... ... ... ... ... G.. ... .G. .A. ... ... --- GAA ... --- ... ... ... ... ... ... ... ... ... ... ... ... ... ...
#Haplotype_22 .T. ... AGT ... T.T ... ... ... ... .C. .A. ... ... ... ... ... G.. ... .G. .A. ... ... GAA GAA ... --- ... ... ... ... ... ... ... ... ... ... ... ... ... ...
#Haplotype_23 ... ... ... ... ... ... ... ... ... ... ... ... ... ... ... ... ... ... ... ... ... ... --- --- ... --- ... ... ... ... ... ... ... ... ... ... ... ... ... ...
#P_cynomolgi  A.. ... ... ... ... ... .C. .T. ... ... .C. T.. ... ... ... ... G.. ... ..A A.. TC. ... --- --- ..A GTC .A. G.. ... C.. ... .T. ... ... ..T ..A .T. ... .A. ...
```

```
              721                                                                                                                                          840
#Haplotype_1  TCT TCG ACA GTC ACT --- --- --- --- CCA TCA GAT GCG AAC GAT GCG AAA ATT AAG TAC TTA GAC AAA CTG TAT GAT GAG GTT CTA ACG ACC TCA GAT AAT ACT AAT GGG ATC CAC GTG
#Haplotype_2  ... ... ... ... ... --- --- --- --- ... ... ... ... ... ... ... ... ... ... ... ... ... ... ... ... ... ... ... ... ..C ... ..G ..C .G. ..C ..G ... ... ... ...
#Haplotype_3  ... ... ... ... ..C --- --- --- --- ... ... ..C ... ... ... ... ... ... ... ... ..G ..T ..G ..C ... ... ... ... ... ..C ... ..G ..C .G. ..C ..G ... ... ... ...
#Haplotype_4  ..A .T. ... .C. ..C --- --- --- --- ... ... ..C ... ... ... ... ... ... ... ... ..G ..T ..G ..C ... ... ... ... ... ..C ... ..G ..C .G. ..C ..G ... ... ... ...
#Haplotype_5  ... ... ... ... ... --- --- --- --- ... ... ... ... ... ... ... ... ... ... ... ... ... ... ... ... ... ... ... ... ..C ... ..G ..C .G. ..C ..G ... ... ... ...
#Haplotype_6  ... ... ... ... ... --- --- --- --- ... ... ... ... ... ... ... ... ... ... ... ... ... ... ... ... ... ... ... ... ..C ... ..G ..C .G. ..C ..G ... ... ... ...
#Haplotype_7  ... ... ... ... ... --- --- --- --- ... ... ..C ... ... ... ... ... ... ... ... ..G ..T ..G ..C ... ... ... ... ... ..C ... ..G ..C .G. ..C ..G ... ... ... ...
#Haplotype_8  A.A .T. ... .C. ..C --- --- --- --- ... ... ..C ... ... ... ... ... ... ... ... ..G ..T ..G ..C ... ... ... ... ... ..C ... ..G ..C .G. ..C ..G ... ... ... ...
#Haplotype_9  A.A .T. ... .C. ..C --- --- --- --- ... ... ..C ... ... ... ... ... ... ... ... ..G ..T ..G ..C ... ... ... ... ... ..C ... ..G ..C .G. ..C ..G ... ... ... ...
#Haplotype_10 ..A .T. ... .C. ..C --- --- --- --- ... ... ..C ... ... ... ... ... ... ... ... ..G ..T ..G ..C ... ... ... ... ... ..C ... ..G ..C .G. ..C ..G ... ... ... ...
#Haplotype_11 ... ... ... ... ... --- --- --- --- ... ... ... ... ... ... ... ... ... ... ... ... ... ... ... ... ... ... ... ... ..C ... ..G ..C .G. ..C ..G ... ... ... ...
#Haplotype_12 A.A .T. ... .C. ..C --- --- --- --- ... ... ..C ... ... ... ... ... ... ... ... ..G ..T ..G ..C ... ... ... ... ... ..C ... ..G ..C .G. ..C ..G ... ... ... ...
#Haplotype_13 A.A .T. ... .C. ..C --- --- --- --- ... ... ..C ... ... ... ... ... ... ... ... ..G ..T ..G ..C ... ... ... ... ... ..C ... ..G ..C .G. ..C ..G ... ... ... ...
#Haplotype_14 A.A .T. ... .C. ..C --- --- --- --- ... ... ..C ... ... ... ... ... ... ... ... ..G ..T ..G ..C ... ... ... ... ... ..C ... ..G ..C .G. ..C ..G ... ... ... ...
#Haplotype_15 ... ... ... ... ... --- --- --- --- ... ... ... ... ... ... ... ... ... ... ... ..G ..T ..G ..C ... ... ... ... ... ..C ... ..G ..C .G. ..C ..G ... ... ... ...
#Haplotype_16 ... ... ... ... ... --- --- --- --- ... ... ... ... ... ... ... ... ... ... ... ... ... ... ... ... ... ... ... ... ..C ... ..G ..C .G. ..C ..G ... ... ... ...
#Haplotype_17 A.A .T. ... .C. ..C --- --- --- --- ... ... ..C ... ... ... ... ... ... ... ... ..G ..T ..G ..C ... ... ... ... ... ..C ... ..G ..C .G. ..C ..G ... ... ... ...
#Haplotype_18 ... ... ... ... ... --- --- --- --- ... ... ..C ... ... ... ... ... ... ... ... ..G ..T ..G ..C ... ... ... ... ... ..C ... ..G ..C .G. ..C ..G ... ... ... ...
#Haplotype_19 A.A .T. ... .C. ..C --- --- --- --- ... ... ..C ... ... ... ... ... ... ... ... ..G ..T ..G ..C ... ... ... ... ... ..C ... ..G ..C .G. ..C ..G ... ... ... ...
#Haplotype_20 ... ... ... ... ... --- --- --- --- ... ... ... ... ... ... ... ... ... ... ... ... ... ... ... ... ... ... ... ... ..C ... ..G ..C .G. ..C ..G ... ... ... ...
#Haplotype_21 ... ... ... ... ... --- --- --- --- ... ... ..C ... ... ... ... ... ... ... ... ..G ..T ..G ..C ... ... ... ... ... ..C ... ..G ..C .G. ..C ..G ... ... ... ...
#Haplotype_22 ... ... ... ... ... --- --- --- --- ... ... ... ... ... ... ... ... ... ... ... ... ... ... ... ... ... ... ... ... ..C ... ..G ..C .G. ..C ..G ... ... ... ...
#Haplotype_23 ... ... ... ... ... --- --- --- --- ... ... ... ... ... ... ... ... ... ... ... ... ..T ..G ..C ... ... ... ... ... ..C ..G ... ..C ... ..C .GC ... ... ... ...
#P_cynomolgi  A.A ... ... ..G ... ACA CCA GAA CGC A.C ..T A.C T.A ... ... .TC ... ... ... ... A.G ..T ..G ... ... ... ..T A.. ... .GT ... ..T .GC ..A ..C ..C .A. ... ... A.A

              841                                                                                                                                          960
#Haplotype_1  CCG GAC TAC CAC AGC AAA TAC AAC ACG ATA AGG CAG AAG TAT GAA TAT AGC ATG AAC CCA GTG GAG TAT GAA ATT GTG AAG AAT CTT TTT AAC GTC GGT TTT AAG AAT GAT GGG GCG GCA
#Haplotype_2  ... ..T ... ... ... ... ... ... ... ... ... ... ... ... ... ... ... ... ... ... ... ... ... ... ... ... ... ... ... ... ... ... ... ... ... ..A ..G ... .AC A.T
#Haplotype_3  ... ..T ... ... ... ... ... ... ... ... ... ... ... ... ... ... ... ... ... ... ... ... ... ... ... ... ... ... ..A ... ... ... ... ... ... ..A ..G ... .AC A.T
#Haplotype_4  ... ..T ... ... ... ... ... ... ... ... ... ... ... ... ... ... ... ... ... ... ... ... ... ... ... ... ... ... ..A ... ... ... ... ... ... ..A ..G ... .AC A.T
#Haplotype_5  ... ..T ... ... ... ... ... ... ... ... ... ... ... ... ... ... ... ... ... ... ... ... ... ... ... ... ... ... ... ... ... ... ... ... ... ... ... ... ... ...
#Haplotype_6  ... ... ... ... ... ... ... ... ... ... ... ... ... ... ... ... ... ... ... ... ... ... ... ... ... ... ... ... ... ... ... ... ... ... ... ... ... ... ... ...
#Haplotype_7  ... ..T ... ... ... ... ... ... ... ... ... ... ... ... ... ... ... ... ... ... ... ... ... ... ... ... ... ... ... ... ... ... ... ... ... ... ... ... ... ...
#Haplotype_8  ... ..T ... ... ... ... ... ... ... ... ... ... ... ... ... ... ... ... ... ... ... ... ... ... ... ... ... ... ..A ... ... ... ... ... ... ..A ..G ... .AC A.T
#Haplotype_9  ... ..T ... ... ... ... ... ... ... ... ... ... ... ... ... ... ... ... ... ... ... ... ... ... ... ... ... ... ... ... ... ... ... ... ... ... ... ... ... ...
#Haplotype_10 ... ..T ... ... ... ... ... ... ... ... ... ... ... ... ... ... ... ... ... ... ... ... ... ... ... ... ... ... ... ... ... ... ... ... ... ... ... ... ... ...
#Haplotype_11 ... ..T ... ... ... ... ... ... ... ... ... ... ... ... ... ... ... ... ... ... ... ..A ... ... ... ... ... ... ... ... ... ... ... ... ... ... ... ... ... ...
#Haplotype_12 ... ..T ... ... ... ... ... ... ... ... ... ... ... ... ... ... ... ... ... ... ... ... ... ... ... ... ... ... ..A ... ... ... ... ... ... ..A ..G ... .AC A.T
#Haplotype_13 ... ..T ... ... ... ... ... ... ... ... ... ... ... ... ... ... ... ... ... ... ... ... ... ... ... ... ... ... ..A ... ... ... ... ... ... ..A ..G ... .AC A.T
#Haplotype_14 ... ..T ... ... ... ... ... ... ... ... ... ... ... ... ... ... ... ... ... ... ... ... ... ... ... ... ... ... ..A ... ... ... ... ... ... ..A ..G ... .AC A.T
#Haplotype_15 ... ..T ... ... ... ... ... ... ... ... ... ... ... ... ... ... ... ... ... ... ... ... ... ... ... ... ... ... ... ... ... ... ... ... ... ... ... ... ... ...
#Haplotype_16 ... ..T ... ... ... ... ... ... ... ... ... ... ... ... ... ... ... ... ... ... ... ..A ... ... ... ... ... ... ... ... ... ... ... ... ... ..A ..G ... .AC A.T
#Haplotype_17 ... ..T ... ... ... ... ... ... ... ... ... ... ... ... ... ... ... ... ... ... ... ... ... ... ... ... ... ... ..A ... ... ... ... ... ... ..A ..G ... .AC A.T
#Haplotype_18 ... ..T ... ... ... ... ... ... ... ... ... ... ... ... ... ... ... ... ... ... ... ... ... ... ... ... ... ... ... ... ... ... ... ... ... ... ... ... ... ...
#Haplotype_19 ... ... ... ... ... ... ... ... ... ... ... ... ... ... ... ... ... ... ... ... ... ... ... ... ... ... ... ... ... ... ... ... ... ... ... ... ... ... ... ...
#Haplotype_20 ... ..T ... ... ... ... ... ... ... ... ... ... ... ... ... ... ... ... ... ... ... ..A ... ... ... ... ... ... ... ... ... ... ... ... ... ..A ..G ... .AC A.T
#Haplotype_21 ... ..T ... ... ... ... ... ... ... ... ... ... ... ... ... ... ... ... ... ... ... ... ... ... ... ... ... ... ... ... ... ... ... ... ... ... ... ... ... ...
#Haplotype_22 ... ... ... ... ... ... ... ... ... ... ... ... ... ... ... ... ... ... ... ... ... ... ... ... ... ... ... ... ..A ... ... ... ... ... ... ..A ..G ... .AC A.T
#Haplotype_23 ... ... ... ... ... ... ... ... ... ... ... ... ... ... ... ... ... ... ... ... ... ... ... ... ... ... ... ... ... ... ... ... ... ... ... ... ... ... ... ...
#P_cynomolgi  ..A CTT ..T ... ... ... ... ... ... ... C.. A.. G.T ..C ... CT. ... ... ..A ..T ..A ..A ..C C.. ... ... ... ... T.A ... ..T ... ..A ... ... ..A ..A ... .AA T.T
```

```
              961                                                                                                                                                    1080
#Haplotype_1  TCT TCA GAT GCT ACT CCC CTA GTT GAC GTT TTT AAA AAA GCG TTA GCT GAC GAG ACA TTT CAA GCC GAG TTT GAT AAC TTT GTA CAT GGG CTG TAC GGA TTT GCT AAA AGG CAT AAC TAC
#Haplotype_2  ..A G.T .C. ... T.C --- ..G ... ... ... ... ... ... ... ... ... ... ... ... ... ... ... ... ... ... ... ... ... ... ... ... ... ... ... ... ... ... ... ... ...
#Haplotype_3  ..A G.T .C. ... T.C --- ..G ... ... ... ... ... ... ... ... ... ... ... ... ... ... ... ... ... ... ... ... ... ... ... ... ... ... ... ... ... ... ... ... ...
#Haplotype_4  ..A G.T .C. ... T.C --- ..G ... ... ... ... ... ... ... ... ... ... ... ... ... ... ... ... ... ... ... ... ... ... ... ... ... ... ... ... ... ... ... ... ...
#Haplotype_5  ... ... ... ... ... ... ... ... ... ... ... ... ... ... ... ... ... ... ... ... ... ... ... ... ... ... ... ... ... ... ... ... ... ... ... ... ... ... ... ...
#Haplotype_6  ... ... ... ... ... ... ... ... ... ... ... ... ... ... ... ... ... ... ... ... ... ... ... ... ... ... ... ... ... ... ... ... ... ... ... ... ... ... ... ...
#Haplotype_7  ... ... ... ... ... ... ... ... ... ... ... ... ... ... ... ... ... ... ... ... ... ... ... ... ... ... ... ... ... ... ... ... ... ... ... ... ... ... ... ...
#Haplotype_8  ..A G.T .C. ... T.C --- ..G ... ... ... ... ... ... ... ... ... ... ... ... ... ... ... ... ... ... ... ... ... ... ... ... ... ... ... ... ... ... ... ... ...
#Haplotype_9  ... ... ... ... ... ... ... ... ... ... ... ... ... ... ... ... ... ... ... ... ... ... ... ... ... ... ... ... ... ... ... ... ... ... ... ... ... ... ... ...
#Haplotype_10 ... ... ... ... ... ... ... ... ... ... ... ... ... ... ... ... ... ... ... ... ... ... ... ... ... ... ... ... ... ... ... ... ... ... ... ... ... ... ... ...
#Haplotype_11 ... ... ... ... ... ... ... ... ... ... ... ... ... ... ... ... ... ... ... ... ... ... ... ... ... ... ... ... ... ... ... ... ... ... ... ... ... ... ... ...
#Haplotype_12 ..A G.T .C. ... T.C --- ..G ... ... ... ... ... ... ... ... ... ... ... ... ... ... ... ... ... ... ... ... ... ... ... ... ... ... ... ... ... ... ... ... ...
#Haplotype_13 ..A G.T .C. ... T.C --- ..G ... ... ... ... ... ... ... ... ... ... ... ... ... ... ... ... ... ... ... ... ... ... ... ... ... ... ... ... ... ... ... ... ...
#Haplotype_14 ..A G.T .C. ... T.C --- ..G ... ... ... ... ... ... ... ... ... ... ... ... ... ... ... ... ... ... ... ... ... ... ... ... ... ... ... ... ... ... ... ... ...
#Haplotype_15 ... ... ... ... ... ... ... ... ... ... ... ... ... ... ... ... ... ... ... ... ... ... ... ... ... ... ... ... ... ... ... ... ... ... ... ... ... ... ... ...
#Haplotype_16 ..A G.T .C. ... T.C --- ..G ... ... ... ... ... ... ... ... ... ... ... .A. ... ... ... ... ... ... ... ... ... ... ... ... ... ... ... ... ... ..A ..C .G. ...
#Haplotype_17 ..A G.T .C. ... T.C --- ..G ... ... ... ... ... ... ... ... ... ... ... ... ... ... ... ... ... ... ... ... ... ... ... ... ... ... ... ... ... ... ... ... ...
#Haplotype_18 ... ... ... ... ... ... ... ... ... ... ... ... ... ... ... ... ... ... ... ... ... ... ... ... ... ... ... ... ... ... ... ... ... ... ... ... ... ... ... ...
#Haplotype_19 ... ... ... ... ... ... ... ... ... ... ... ... ... ... ... ... ... ... ... ... ... ... ... ... ... ... ... ... ... ... ... ... ... ... ... ... ... ... ... ...
#Haplotype_20 ..A G.T .C. ... T.C --- ..G ... ... ... ... ... ... ... ... ... ... ... .A. ... ... ... ... ... ... ... ... ... ... ... ... ... ... ... ... ... ..A ..C .G. ...
#Haplotype_21 ... ... ... ... ... ... ... ... ... ... ... ... ... ... ... ... ... ... ... ... ... ... ... ... ... ... ... ... ... ... ... ... ... ... ... ... ... ... ... ...
#Haplotype_22 ..A G.T .C. ... T.C --- ..G ... ... ... ... ... ... ... ... ... ... ... ... ... ... ... ... ... ... ... ... ... ... ... ... ... ... ... ... ... ... ... ... ...
#Haplotype_23 ... ... ... ... ... ... ... ... ... ... ... ... ... ... ... ... ... ... ... ... ... ... ... ... ... ... ... ... ... ... ... ... ... ... ... ... ... ... ... ...
#P_cynomolgi  ..A G.T .C. AA. T.C --- ..G ACG ..A ... ... ... ... .TC ... .TA ..T ..A .A. ... ... .A. ..A ... A.C ... ... ..G ..C ... ... ..T ... ... ... ..G ..A ..C ... ..T

             1081                                                                                                     1167
#Haplotype_1  CTA AGT GAG GCG CGG ATG AAG GAT GCT GAT CGC TAC ACC AAT CTG CTA AAG AAC GCC ATC AGC TTG ATG TAC ACC ATT --- --- ---
#Haplotype_2  ... ... ... ... ... ... ... ... ... ... ... ... ... ... ... ... ... ... ... ... ... ... ... ... ... ... --- --- ---
#Haplotype_3  ... ... ... ... ... ... ... .G. ... ... ... ... ... ... ... ... ... ... ... ... ... ... ... ... ... ... --- --- ---
#Haplotype_4  ... ... ... ... ... ... ... ... ... ... ... ... ... ... ... ... ... ... ... ... ... ..C ... ... ... ... --- --- ---
#Haplotype_5  ... ... ... ... ... ... ... ... ... ... ... ... ... ... ... ... ... ... ... ... ... --- --- --- --- --- --- --- ---
#Haplotype_6  ... ... ... ... ... ... ... ... ... ... ... ... ... ... ... ... ... ... ..A ... ... --- --- --- --- --- --- --- ---
#Haplotype_7  ... ... ... ... ... ... ... ..C ... ... ... ... ... ... ... ... ... ... ..A ... ... --- --- --- --- --- --- --- ---
#Haplotype_8  ... ... ... ... ... ... ... ... ... ... ... ... ... ... ... ... ... ... ... ... ... --- --- --- --- --- --- --- ---
#Haplotype_9  ... ... ... ... ... ... ... ... ... ... ... ... ... ... ... ... ... ... ... ... ... --- --- --- --- --- --- --- ---
#Haplotype_10 ... ... ... ... ... ... ... ... ... ... ... ... ... ... ... ... ... ... ... ... ... --- --- --- --- --- --- --- ---
#Haplotype_11 ... ... ... ... ... ... ... ... ... ... ... ... ... ... ... ... ... ... ... ... ... --- --- --- --- --- --- --- ---
#Haplotype_12 ... ... ... ... ... ... ... ... ... ... ... ... ... ... ... ... ... ... ... ... ... --- --- --- --- --- --- --- ---
#Haplotype_13 ... ... ... ... ... ... ... ... ... ... ... ... ... ... ... ... ... ... ... ... ... --- --- --- --- --- --- --- ---
#Haplotype_14 ... ... ... ... ... ... ... ... ... ... ... ... ... ... ... ... ... ... ... ... ... --- --- --- --- --- --- --- ---
#Haplotype_15 ... ... ... ... ... ... ... ... ... ... ... ... ... ... ... ... ... ... ... ... ... --- --- --- --- --- --- --- ---
#Haplotype_16 T.. ... ... ..A ..A ... ... ..C AA. A.A .T. ... T.. G.. T.. ..G ..A ... ... ... ... --- --- --- --- --- --- --- ---
#Haplotype_17 ... ... ... ... ... ... ... ..C ... ... ... ... ... ... ... ... ... ... ... ... ... --- --- --- --- --- --- --- ---
#Haplotype_18 ... ... ... ... ... ... ... ..C ... ... ... ... ... ... ... ... ... ... ... ... ... --- --- --- --- --- --- --- ---
#Haplotype_19 ... ... ... ... ... ... ... ... ... ... ... ... ... ... ... ... ... ... ... ... ... --- --- --- --- --- --- --- ---
#Haplotype_20 T.. ... ... ..A ..A ... ... ..C AA. A.A .T. ... T.. G.. T.. ..G ... ... ... ... ... --- --- --- --- --- --- --- ---
#Haplotype_21 ... ... ... ... ... ... ... ..C ... ... ... ... ... ... ... ... ... ... ..A ... ... --- --- --- --- --- --- --- ---
#Haplotype_22 ... ... ... ... ... ... ... ..C ... ... ... ... ... ... ... ... ... ... ... ... ... --- --- --- --- --- --- --- ---
#Haplotype_23 ... ... ... ... ... ... ... ... ... ... ... ... ... ... ... ... ... ... ... ... ... --- --- --- --- --- --- --- ---
#P_cynomolgi  T.. G.. A.T AAA ..A C.T G.. A.C A.G ACA GT. G.. T.. G.. ... ... ... ... ... T.. ... ... ... A.. ..T C.. GAG GTG GCG
```

The alignment shows the 23 haplotypes found in *pvmsp-7E* together with the *pcmsp-7E* haplotype. Haplotype 1, Sal-I; Haplotype 2, Brazil-I; Haplotype 3, India-VII; Haplotype 4, Mauritania-I; Haplotype 5-23, Colombian isolates. Dots represent nucleotide identity. Codons under positive selection are shown in green (intra-species) and turquoise (inter-species) and those under negative selection are shown in yellow (intra-species) and fuchsia (inter-species).
