# Supplementary material for: Heterogeneous genetic diversity pattern in Plasmodium vivax genes encoding merozoite surface proteins (MSP) -7E, −7F and -7L
Source: Malar J. 2014 Dec 13;13:495. doi: 10.1186/1475-2875-13-495 (PMC4300842; doi:10.1186/1475-2875-13-495)
Supplement: Supplementary file 5 — Additional file 5: Haplotype alignment at PvMSP-7E protein level in Colombia. The alignment shows the 13 haplotypes at protein level found in PvMSP-7E. Dots represent amino acid identity. (PDF 74 KB) [file 12936_2014_3635_MOESM5_ESM.pdf]

Heterogeneous genetic diversity pattern in *Plasmodium vivax* genes encoding merozoite surface proteins (MSP) -7E, -7F and -7L

Additional file 5 Haplotype alignment at *pvmsp-7E* protein level in Colombia.

```
            18                                                                                                                  135
Haplotype_5  CASSEKLGVQ KKKKNLEQDA THALMKKLES LYKLSATDNS EIFNKEIESL KKQIDQLHQH GGENEGESLG HLLESEAANE SAKKTIFGVD EDDLDNYDAD FIGQSKGKIK GQADTDNQ
Haplotype_7  .......... .......... .......... .......... .......... .......... .......... .......... .......... .......... .......... ........
Haplotype_8  .......... .R........ .......... .......... .......... .......... .....E.... ..I....... .......... ...S...... ......R... ...VA..E
Haplotype_9  .......... .......... .......... .......... .......... .......... .......... .......... .......... .......... .......... ..TEGGDR
Haplotype_10 .......... .......... .......... .......... .......... .......... .......... .......... .......... .......... .......... ........
Haplotype_11 .......... .......... .......... .......... .......... .......... .......... .......... .......... .......... .......... ........
Haplotype_14 .......... .R........ .......... .......... .......... .......... .....E.... ..I....... .......... ...S...... ......R... ...VA..E
Haplotype_16 .......... .......... .......... .......... .......... .......... .......... .......... .......... .......... .......... ........
Haplotype_17 .......... .R........ .......... .......... .......... .......... .....E.... ..I....... .......... .......... .......... ..TEGGDR
Haplotype_18 .......... .......... .......... .......... .......... .......... .......... .......... .......... .......... .......... ........
Haplotype_20 .......... .......... .......... .......... .......... .......... .......... .......... .......... .......... .......... ........
Haplotype_22 .......... .......... .......... .......... .......... .......... .......... .......... .......... .......... .......... ........
Haplotype_23 .......... .......... .......... .......... .......... .......... .......... .......... .......... .......... .......... ........
           136                                                                                                                  264
Haplotype_5  AQ-RTADVAA QPGGVSPSTS ARPQEPGKTG VTGSPNGLVE AGLVNTKTLQ NVGPNGQRAA DPQPGRAANL PEGQRTNDPQ Q--GGSESTE GPAVTPRPSS TVTPSDANDA KIKYLDKL
Haplotype_7  ..-....... ........AG .QSRDTARPE A.DR...V.. R.F.D.R... ...D...PTV .S.S....TQ .....A.GH. .EE....... .......... .......... ........
Haplotype_8  ..RAPDNLP. PQ.RELSAA. GQ...SARPQ .....GSQI. G.F...R..E ..EA....V. ...SRP..TQ ....GA.G.. .--.ERAP.. RT....S.TL .A........ ........
Haplotype_9  T.-SP..... PAR....... .......... .......... ..S...R..E ..EA....V. ...SRP..TQ ....GA.G.. .--.ERAP.. RT....S.TL .A........ ........
Haplotype_10 ..-....... .....L..AG .QSRDTARPE A......... ......R..E ..EA....V. ...SRP..TQ ....GA.G.. .--.ERAP.. RT....S..L .A........ ........
Haplotype_11 ..-....... .....L..AG .QSRDTARPE A.DR...V.. R.F.D.R... ...D...PTV .S.S....TQ .....A.GH. .-E....... .......... .......... ........
Haplotype_14 ..RAPDNLP. PQ.RELSAA. GQ...SAR.Q .....GRQI. G.F...R..E ..EA....V. ...SRP..TQ ....GA.G.. .--.ERAP.. RT....S.TL .A........ ........
Haplotype_16 ..-....... .....L..AG .QSRDTARPE A.DR...V.. R.F.D.R... ...D...PTV .S.S....TQ .....A.GH. .-E....... .......... .......... ........
Haplotype_17 T.-SP..... PQ.RELSAA. GQ...SARPQ .....GSQI. G.F...R..E ..EA....V. ...SRP..TQ ....GA.G.. .--.ERAP.. RT....S.TL .A........ ........
Haplotype_18 ..-....... ........AG .QSRDTARPE A.DR...V.. R.F.D.R... ...D...PTV .S.S....TQ .....A.GH. .-E....... .......... .......... ........
Haplotype_20 ..-....... .....L..AG .QSRDTARPE A.DR...V.. R.F.D.R... ...D...PTV .S.S....TQ .....A.GH. .EE....... .......... .......... ........
Haplotype_22 ..-....... ........AG .QSRDTARPE A.DR...V.. R.F.D.R... ...D...PTV .S.S....TQ .....A.GH. .EE....... .......... .......... ........
Haplotype_23 ..-....... .......... .......... .......... .......... .......... .......... .......... .--....... .......... .......... ........
            265                                                                                                                381
Haplotype_5  YDEVLTTSDS TKGIHVPDYH SKYNTIRQKY EYSMNPVEYE IVKNLFNVGF KNDGAASSDA TPLVDVFKKA LADETFQAEF DNFVHGLYGF AKRHNYLSEA RMKDADRYTN LLKNAIS
Haplotype_7  .......... .......... .......... .......... .......... .......... .......... .......... .......... .......... .......... .......
Haplotype_8  .......... .......... .......... .......... .......... .KE.DT.AA. S-........ .......... .......... .......... .......... .......
Haplotype_9  .......... .......... .......... .......... .......... .......... .......... .......... .......... .......... .......... .......
Haplotype_10 .......... .......... .......... .......... .......... .......... .......... .......... .......... .......... .......... .......
Haplotype_11 .......... .......... .......... .......... .......... .......... .......... .......... .......... .......... .......... .......
Haplotype_14 .......... .......... .......... .......... .......... .KE.DT.AA. S-........ .......... .......... .......... .......... .......
Haplotype_16 .......... .......... .......... .......... .......... .KE.DT.AA. S-........ ....K..... .......... ....S..... ....NKL.SD .......
Haplotype_17 .......... .......... .......... .......... .......... .KE.DT.AA. S-........ .......... .......... .......... .......... .......
Haplotype_18 .......... .......... .......... .......... .......... .......... .......... .......... .......... .......... .......... .......
Haplotype_20 .......... .......... .......... .......... .......... .KE.DT.AA. S-........ ....K..... .......... ....S..... ....NKL.SD .......
Haplotype_22 .......... .......... .......... .......... .......... .KE.DT.AA. S-........ .......... .......... .......... .......... .......
Haplotype_23 .........N .S........ .......... .......... .......... .......... .......... .......... .......... .......... .......... .......
```

The alignment shows the 13 haplotypes at protein level found in *pvmsp-7E*. Dots represent amino acid identity.
