# Supplementary material for: Heterogeneous genetic diversity pattern in Plasmodium vivax genes encoding merozoite surface proteins (MSP) -7E, −7F and -7L
Source: Malar J. 2014 Dec 13;13:495. doi: 10.1186/1475-2875-13-495 (PMC4300842; doi:10.1186/1475-2875-13-495)
Supplement: Supplementary file 6 — Additional file 6: Neutrality, linkage disequilibrium and recombination tests at the 5′-end, central region and 3′-end for pvmsp-7 genes in the Colombian population. 5′-end (pvmsp-7E: nucleotide 1–390, pvmsp-7F: nucleotide 1–432, pvmsp-7L: nucleotide 1–381), central (pvmsp-7E: nucleotide 391–747, pvmsp-7F: nucleotide 433–1,053, pvmsp-7L: nucleotide 382–816) and 3′-end (pvmsp-7E: nucleotide 748–1,158, pvmsp-7F: nucleotide 1,054–1,449, pvmsp-7L: nucleotide 817–1,275). Numbers based on Additional files 4, 9 and 10. •: p <0.02, *: p <0.05. (PDF 203 KB) [file 12936_2014_3635_MOESM6_ESM.pdf]

Heterogeneous genetic diversity pattern in *Plasmodium vivax* genes encoding merozoite surface proteins (MSP) -7E, -7F and -7L

Additional file 6 Neutrality, linkage disequilibrium and recombination tests at the 5'-end, central region and 3'-end for *pvmsp-7* genes in the Colombian population.

	n	Gene	Tajima	Fu & Li		Fay & Wu	Fu	K-test	H-test (sd)	Z_{nS}	ZZ	RM
			D	D*	F*	H	Fs					
msp-7E		5'-end	0,7753	1,5845•	1,5597	-1,3741	7,723•	4*	0.331 (0.02) *	0,876*	0,036	1
	31	central	2,0870*	1,7901•	2,2375•	-18,086	20,896•	9*	0.855 (0.02) *	0,317*	0,293*	6
		3'-end	0,1114	0,8811	0,7404	-31,542*	1,255	14	0.872 (0.03)	0,304*	0,507*	4

	n	Gene	Tajima	Fu & Li		Fay & Wu	Fu	K-test	H-test (sd)	Z_{nS}	ZZ	RM
			D	D*	F*	H	Fs					
msp-7F		5'-end	nd	nd	nd	nd	nd	1	0.000	nd	nd	nd
	36	central	0,69854	0,57433	0,70231	0.000	1,099	2	0.346 (0.07)	nd	nd	0
		3'-end	1,4254	0,57433	0,93746	-0,365	1,642	2	0.461 (0.04)	nd	nd	0

	n	Gene	Tajima	Fu & Li		Fay & Wu	Fu	K-test	H-test (sd)	Z_{nS}	ZZ	RM
			D	D*	F*	H	Fs					
msp-7L		5'-end	nd	nd	nd	nd	nd	1	0.000	nd	nd	nd
	31	central	-0,5820	0,8019	0,47341	0,31398	-0,56	3	0.329 (0.09)	0,0102	0.000	0
		3'-end	-0,3903	0,8019	0,53615	0,34194	-0,33	3	0.370 (0.09)	0,0133	0.000	0

5'-end (*pvmsp-7E*: nucleotide 1–390, *pvmsp-7F*: nucleotide 1–432, *pvmsp-7L*: nucleotide 1–381), central (*pvmsp-7E*: nucleotide 391–747, *pvmsp-7F*: nucleotide 433–1,053, *pvmsp-7L*: nucleotide 382–816) and 3'-end (*pvmsp-7E*: nucleotide 748–1,158, *pvmsp-7F*: nucleotide 1,054–1,449, *pvmsp-7L*: nucleotide 817–1,275). Numbers based on Additional files 4, 9 and 10. •: $p < 0.02$, *: $p < 0.05$.

.
