# Supplementary figures and images for: Heterogeneous genetic diversity pattern in Plasmodium vivax genes encoding merozoite surface proteins (MSP) -7E, −7F and -7L
Source: Malar J. 2014 Dec 13;13:495. doi: 10.1186/1475-2875-13-495 (PMC4300842; doi:10.1186/1475-2875-13-495)

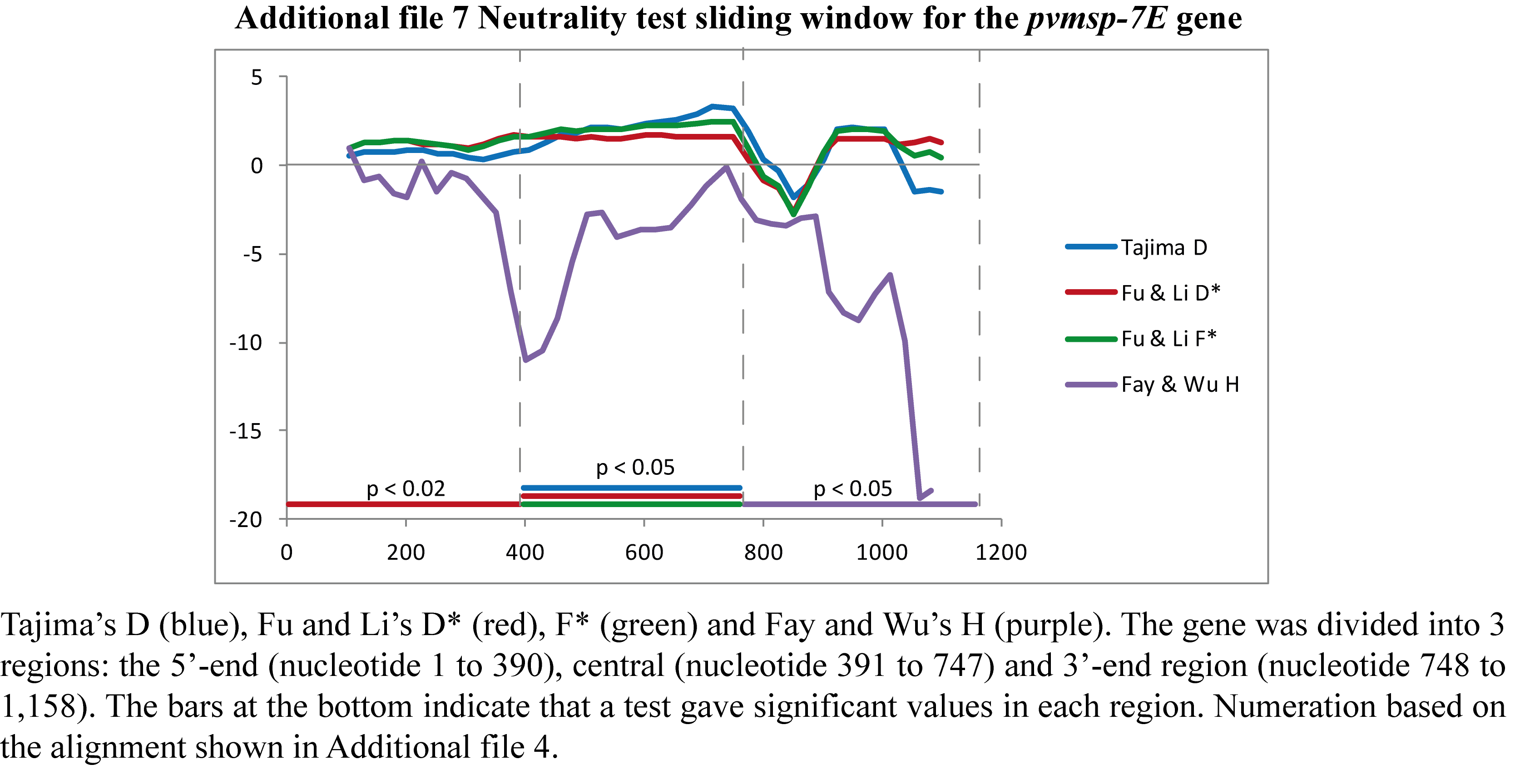

Supplement: Supplementary file 7 — Additional file 7: Neutrality test sliding window for the pvmsp-7E gene. Tajima’s D (blue), Fu and Li’s D* (red), F* (green) and Fay and Wu’s H (purple). The gene was divided into 3 regions: the 5′-end (nucleotide 1 to 390), central (nucleotide 391 to 747) and 3′-end region (nucleotide 748 to 1,158). The bars at the bottom indicate that a test gave significant values in each region. Numbering based on the alignment shown in Additional file 4. (TIFF 523 KB) [file 12936_2014_3635_MOESM7_ESM.tiff]
