# Supplementary material for: Heterogeneous genetic diversity pattern in Plasmodium vivax genes encoding merozoite surface proteins (MSP) -7E, −7F and -7L
Source: Malar J. 2014 Dec 13;13:495. doi: 10.1186/1475-2875-13-495 (PMC4300842; doi:10.1186/1475-2875-13-495)
Supplement: Supplementary file 8 — Additional file 8: Intra-species positively and negatively selected sites detected for pvmsp-7 genes. 5′-end (pvmsp-7E: nucleotide 1–390, pvmsp-7F: nucleotide 1–432, pvmsp-7L: nucleotide 1–381), central (pvmsp-7E: nucleotide 391–747, pvmsp-7F: nucleotide 433–1,053, pvmsp-7L: nucleotide 382–816) and 3′-end (pvmsp-7E: nucleotide 748–1,158, pvmsp-7F: nucleotide 1,054–1,449, pvmsp-7L: nucleotide 817–1,275). Numbers based on Additional files 4, 9 and 10. (PDF 57 KB) [file 12936_2014_3635_MOESM8_ESM.pdf]

Heterogeneous genetic diversity pattern in *Plasmodium vivax* genes encoding merozoite surface proteins (MSP) -7E, -7F and -7L

Additional file 8 Intra-species positively and negatively selected sites detected for *pvmsp-7* genes.

Positively selected sites			
**Gene**	**5'-end**	**central**	**3'-end**
msp-7E	-	132, 147, 151, 164, 171, 178, 200, 239	369
msp-7F	-	-	424
msp-7L	-	159, 260	357
**Negatively selected sites**			
**Gene**	**5'-end**	**central**	**3'-end**
msp-7E	46, 48, 52, 56, 63, 64, 72, 100, 127	149, 236, 245	252, 261, 262, 263, 264, 273, 282, 302, 309, 357, 358, 368, 377, 379
msp-7F	-	-	-
msp-7L	-	-	-

5’-end (*pvmsp-7E*: nucleotide 1–390, *pvmsp-7F*: nucleotide 1–432, *pvmsp-7L*: nucleotide 1–381), central (*pvmsp-7E*: nucleotide 391–747, *pvmsp-7F*: nucleotide 433–1,053, *pvmsp-7L*: nucleotide 382–816) and 3’-end (*pvmsp-7E*: nucleotide 748–1,158, *pvmsp-7F*: nucleotide 1,054–1,449, *pvmsp-7L*: nucleotide 817–1,275). Numbers based on Additional files 4, 9 and 10.
