# Supplementary material for: Heterogeneous genetic diversity pattern in Plasmodium vivax genes encoding merozoite surface proteins (MSP) -7E, −7F and -7L
Source: Malar J. 2014 Dec 13;13:495. doi: 10.1186/1475-2875-13-495 (PMC4300842; doi:10.1186/1475-2875-13-495)
Supplement: Supplementary file 9 — Additional file 9: pvmsp-7F gene alignment. The alignment shows the 8 haplotypes found in pvmsp-7F together with pcmsp-7F haplotype. Haplotype 1, Sal-I; haplotype 2, Brazil-I and North Korea; haplotype 3, India-VII; haplotype 4, Mauritania-I; haplotypes 5–8, Colombian isolates. Dots represent nucleotide identity. Codons under positive selection are shown in green (intra-species) and turquoise (inter-species) and those under negative selection are shown in fuchsia (inter-species). (PDF 99 KB) [file 12936_2014_3635_MOESM9_ESM.pdf]

Additional file 9 *pvmsp-7F* gene alignment

```
             1                                                                                                                      120
#Haplotype_1 ATG GGG GGA AAC AAA AAT GCT CTC TTC CTC TCC TTG CTG ATA CTC CTC --- CTC --- TGC TGC GCA TGG TGT GAA AAA CCC GGA GTG GAA AAA AAA AAA AAG TTG GAG GAA GAC GTC ATA
#Haplotype_2 ... ... ... ... ... ... ... ... ... ... ... ... ... ... ... ... --- ... --- ... ... ... ... ... ... ... ... ... ... ... ... ... ... ... ... ... ... ... ... ...
#Haplotype_3 ... ... ... ... ... .G. ... ... ... ... ... ... ... ... ... ... --- ... --- ... ... ... ... ... ... ... ... ... ... ... ... ... ... ... ... ... ... ... ... ...
#Haplotype_4 ... ... ... ... ... ... ... ... ... ... ... ... ... ... ... ... --- ... --- ... ... ... ... ... ... ... ... ... ... ... ... ... ... ... ... ... ... ... ... ...
#Haplotype_5 --- --- --- --- --- --- --- --- --- --- --- --- --- --- --- --- --- ... --- ... ... ... ... ... ... ... ... ... ... ... ... ... ... ... ... ... ... ... ... ...
#Haplotype_6 --- --- --- --- --- --- --- --- --- --- --- --- --- --- --- --- --- ... --- ... ... ... ... ... ... ... ... ... ... ... ... ... ... ... ... ... ... ... ... ...
#Haplotype_7 --- --- --- --- --- --- --- --- --- --- --- --- --- --- --- --- --- ... --- ... ... ... ... ... ... ... ... ... ... ... ... ... ... ... ... ... ... ... ... ...
#Haplotype_8 --- --- --- --- --- --- --- --- --- --- --- --- --- --- --- --- --- ... --- ... ... ... ... ... ... ... ... ... ... ... ... ... ... ... ... ... ... ... ... ...
#P_cynomolgi ... ..T .A. ... ... .T. ..A ... ... ... ... ACC G.. ... ... ... CTT ... TGC ... ... .T. ... ... ... ... T.T ... ... ... ..T ... ... ..A ... ... ... ..T ... ...
           121                                                                                                                      240
#Haplotype_1 GGC ATA CTG AGG AGG AAG CTG GAG AGT TTG CAA AAA CGC AGC TTA ACC AAT TCG GAT GGA AAA TTG AAG AAA GAA ATT GAA TTG GTG AAG AAG CAA ATT CAA GAA TTG CAG AAA TAT GAA
#Haplotype_2 ... ... ... ... ... ... ... ... ... ... ... ... ... ... ... ... ... ... ... ... ... ... ... ... ... ... ... ... ... ... ... ... ... ... ... ... ... ... ... ...
#Haplotype_3 ... ... ... ... ... ... ... ... ... ... ... ... ... ... ... ... ... ... ... ... ... ... ... ... ... ... ... ... ... ... ... ... ... ... ... ... ... ... ... ...
#Haplotype_4 ... ... ... ... ... ... ... ... ... ... ... ... ... ... ... ... ... ... ... ... ... ... ... ... ... ... ... ... ... ... ... ... ... ... ... ... ... ... ... ...
#Haplotype_5 ... ... ... ... ... ... ... ... ... ... ... ... ... ... ... ... ... ... ... ... ... ... ... ... ... ... ... ... ... ... ... ... ... ... ... ... ... ... ... ...
#Haplotype_6 ... ... ... ... ... ... ... ... ... ... ... ... ... ... ... ... ... ... ... ... ... ... ... ... ... ... ... ... ... ... ... ... ... ... ... ... ... ... ... ...
#Haplotype_7 ... ... ... ... ... ... ... ... ... ... ... ... ... ... ... ... ... ... ... ... ... ... ... ... ... ... ... ... ... ... ... ... ... ... ... ... ... ... ... ...
#Haplotype_8 ... ... ... ... ... ... ... ... ... ... ... ... ... ... ... ... ... ... ... ... ... ... ... ... ... ... ... ... ... ... ... ... ... ... ... ... ... ... ... ...
#P_cynomolgi A.. ... ... ... ... ..A ..C ... ... ... ... ... ..T ... .TA ... ... ... ... ... ... ..T ..A ... ... ... ... ... ... ... .T. ... ... ... ..C ... ... ... ... ...
           241                                                                                                                      360
#Haplotype_1 AAG GGA GAG GCT GGC AAA AAG GTG GAC GCC ACT TTG GGG GAG GAA CCA GGA GTC GAG TCT GCA GAG GAG CAG CCT TTA AGT GTG GAG GAG GCA GGA GAC ACA CAA GAT GAA GAT --- ---
#Haplotype_2 ... ... ... ... ... ... ... ... ... ... ... ... ... ... ... ... ... ... ... ... ... ... ... ... ... ... ... ... ... ... ... ... ... ... ... ... ... ... --- ---
#Haplotype_3 ... ... ... ... ... ... ... ... ... ... ... ... ... ... ... ... ... ... ... ... ... ... ... ... ... ... ... ... ... ... ... ... ... ... ... ... ... ... --- ---
#Haplotype_4 ... ... ... ... ... ... ... ... ... ... ... ... ... ... ... ... ... ... ... ... ... ... ... ... ... ... ... ... ... ... ... ... ... ... ... ... ... ... --- ---
#Haplotype_5 ... ... ... ... ... ... ... ... ... ... ... ... ... ... ... ... ... ... ... ... ... ... ... ... ... ... ... ... ... ... ... ... ... ... ... ... ... ... --- ---
#Haplotype_6 ... ... ... ... ... ... ... ... ... ... ... ... ... ... ... ... ... ... ... ... ... ... ... ... ... ... ... ... ... ... ... ... ... ... ... ... ... ... --- ---
#Haplotype_7 ... ... ... ... ... ... ... ... ... ... ... ... ... ... ... ... ... ... ... ... ... ... ... ... ... ... ... ... ... ... ... ... ... ... ... ... ... ... --- ---
#Haplotype_8 ... ... ... ... ... ... ... ... ... ... ... ... ... ... ... ... ... ... ... ... ... ... ... ... ... ... ... ... ... ... ... ... ... ... ... ... ... ... --- ---
#P_cynomolgi ... ..G .GT .T. ..A ... ... ... ... T.. ... ... ... ... ... T.G ... ... C.. ... .T. A.. .GA ..A ... ..G ... T.. ..A ... ... ... ... ... ... ... ... ... CGT ATG
           361                                                                                                                      480
#Haplotype_1 --- --- --- --- CGT TTG GAT GAA TTA GAA GGT GTA GAA GAC TTT GAG GAG GAG AAC CTT GAG CAA TCG GAG CAA GTG GAG GAG GCA GAG GTA GTC GAA GAA GCG GAG GAA GAA GCG GGG
#Haplotype_2 --- --- --- --- ... ... ... ... ... ... ... ... ... ... ... ... ... ... ... ... ... ... ... ... ... ... ... ... ... ... ... ... ... ... ... ... ... ... ... ...
#Haplotype_3 --- --- --- --- ... ... ... ... ... ... ... ... ... ... ... ... ... ... ... ... ... ... ... ... ... ... ... ... ... ... ... ... ... ... ... --- --- --- --- ...
#Haplotype_4 --- --- --- --- ... ... ... ... ... ... ... ... ... ... ... ... ... ... ... ... ... ... ... ... ... ... ... ... ... ... ... ... ... ... ... ... ... ... ... ...
#Haplotype_5 --- --- --- --- ... ... ... ... ... ... ... ... ... ... ... ... ... ... ... ... ... ... ... ... ... ... ... ... ... ... ... ... ... ... ... ... ... ... ... ...
#Haplotype_6 --- --- --- --- ... ... ... ... ... ... ... ... ... ... ... ... ... ... ... ... ... ... ... ... ... ... ... ... ... ... ... ... ... ... ... ... ... ... ... ...
#Haplotype_7 --- --- --- --- ... ... ... ... ... ... ... ... ... ... ... ... ... ... ... ... ... ... ... ... ... ... ... ... ... ... ... ... ... ... ... ... ... ... ... ...
#Haplotype_8 --- --- --- --- ... ... ... ... ... ... ... ... ... ... ... ... ... ... ... ... ... ... ... ... ... ... ... ... ... ... ... ... ... ... ... ... ... ... ... ...
#P_cynomolgi CAT GAT TCA TAT ..A ... ... .GC ... ... .C. ... ... ... .A. ... ... ... .T. ... .G. ... G.. ... .C. ... ... .C. ..T A.. ... ..T ... ... ... --- --- --- --- ...
           481                                                                                                                      600
#Haplotype_1 GAT GCG GAA GAG GAG CAG CCC GCG GAG GCT GAG GAG GAT GGC AGC TTA CTA GAA GAG GCT CCC AAC TCG GTA GAA AGA AAA GCA GAA GGT GCC ATC GCT GAA TTT GAA GAG GCA GAT GTG
#Haplotype_2 ... ... ... ... ... ... ... ... ... ... ... ... ... ... ... ... ... ... ... ... ... ... ... ... ... ... ... ... ... ... ... ... ... ... ... ... ... ... ... ...
#Haplotype_3 ... ... ... ... ... ... ... ... ... ... ... ... ... ... ... ... ... ... ... ... ... ... ... ... ... ... ... ... ... ... ... ... ... ... ... ... ... ... ... ...
#Haplotype_4 ... ... ... ... ... ... ... ... ... ... ... ... ... ... ... ... ... ... ... ... ... ... ... ... ... ... ... ... ... ... ... ... ... ... ... ... ... ... ... ...
#Haplotype_5 ... ... ... ... ... ... ... ... ... ... ... ... ... ... ... ... ... ... ... ... ... ... ... ... ... ... ... ... ... ... ... ... ... ... ... ... ... ... ... ...
#Haplotype_6 ... ... ... ... ... ... ... ... ... ... ... ... ... ... ... ... ... ... ... ... ... ... ... ... ... ... ... ... ... ... ... ... ... ... ... ... ... ... ... ...
#Haplotype_7 ... ... ... ... ... ... ... ... ... ... ... ... ... ... ... ... ... ... ... ... ... ... ... ... ... ... ... ... ... ... ... ... ... ... ... ... ... ... ... ...
#Haplotype_8 ... ... ... ... ... ... ... ... ... ... ... ... ... ... ... ... ... ... ... ... ... ... ... ... ... ... ... ... ... ... ... ... ... ... ... ... ... ... ... ...
#P_cynomolgi ... ... ... ... ... G.. .T. ..T ..T .GC ... .T. ... AA. ... ... GC. ..C .C. .TC ..T G.. ..C ... ... ... .CC .A. ... ... .TA ..G .A. ... ..C ... ... ... ..A .AA
           601                                                                                                                      720
#Haplotype_1 GAA GAA GGT GCG GAA GCG GAT GAA GGT GTC GAA ACG GAT GAA GGG --- --- --- --- --- --- --- --- --- --- --- --- --- --- --- --- --- --- --- --- --- --- --- --- ---
#Haplotype_2 ... ... ... ... ... ... ... ... ... ... ... G.. ... ... ... --- --- --- --- --- --- --- --- --- --- --- --- --- --- --- --- --- --- --- --- --- --- --- --- ---
#Haplotype_3 ... ... ... ... ... ... ... ... ... ... ... G.. ... ... ... --- --- --- --- --- --- --- --- --- --- --- --- --- --- --- --- --- --- --- --- --- --- --- --- ---
#Haplotype_4 ... ... ... ... ... ... ... ... ... ... ... G.. ... ... ... --- --- --- --- --- --- --- --- --- --- --- --- --- --- --- --- --- --- --- --- --- --- --- --- ---
#Haplotype_5 ... ... ... ... ... ... ... ... ... ... ... ... ... ... ... --- --- --- --- --- --- --- --- --- --- --- --- --- --- --- --- --- --- --- --- --- --- --- --- ---
#Haplotype_6 ... ... ... ... ... ... ... ... ... ... ... G.. ... ... ... --- --- --- --- --- --- --- --- --- --- --- --- --- --- --- --- --- --- --- --- --- --- --- --- ---
#Haplotype_7 ... ... ... ... ... ... ... ... ... ... ... G.. ... ... ... --- --- --- --- --- --- --- --- --- --- --- --- --- --- --- --- --- --- --- --- --- --- --- --- ---
#Haplotype_8 ... ... ... ... ... ... ... ... ... ... ... ... ... ... ... --- --- --- --- --- --- --- --- --- --- --- --- --- --- --- --- --- --- --- --- --- --- --- --- ---
#P_cynomolgi ..C ... ..G ..A ... .AA ..C ... ... .CA ..T TT. ... ... .CT AAC TTA GAA AGT TCC ACT TCG GCA AGC AGT GAG CTA GTA GGG GGA TCC GCA GTG GGA GGA AGC ACA GAG GAG GAG
           721                                                                                                                      840
#Haplotype_1 --- --- --- --- --- --- --- --- --- --- --- --- --- --- --- --- --- --- --- --- --- --- --- --- --- --- --- --- --- --- --- --- --- --- --- --- --- --- --- GCA
#Haplotype_2 --- --- --- --- --- --- --- --- --- --- --- --- --- --- --- --- --- --- --- --- --- --- --- --- --- --- --- --- --- --- --- --- --- --- --- --- --- --- --- ...
#Haplotype_3 --- --- --- --- --- --- --- --- --- --- --- --- --- --- --- --- --- --- --- --- --- --- --- --- --- --- --- --- --- --- --- --- --- --- --- --- --- --- --- ...
#Haplotype_4 --- --- --- --- --- --- --- --- --- --- --- --- --- --- --- --- --- --- --- --- --- --- --- --- --- --- --- --- --- --- --- --- --- --- --- --- --- --- --- ...
#Haplotype_5 --- --- --- --- --- --- --- --- --- --- --- --- --- --- --- --- --- --- --- --- --- --- --- --- --- --- --- --- --- --- --- --- --- --- --- --- --- --- --- ...
#Haplotype_6 --- --- --- --- --- --- --- --- --- --- --- --- --- --- --- --- --- --- --- --- --- --- --- --- --- --- --- --- --- --- --- --- --- --- --- --- --- --- --- ...
#Haplotype_7 --- --- --- --- --- --- --- --- --- --- --- --- --- --- --- --- --- --- --- --- --- --- --- --- --- --- --- --- --- --- --- --- --- --- --- --- --- --- --- ...
#Haplotype_8 --- --- --- --- --- --- --- --- --- --- --- --- --- --- --- --- --- --- --- --- --- --- --- --- --- --- --- --- --- --- --- --- --- --- --- --- --- --- --- ...
#P_cynomolgi GAG GAG AAA GAG AAG GAA AAA GAA GAA GAA CAG GGA GAA GAA GAC CTG CAG GGG ATC TTC CAG TTA GAG GAA GAA CAG GGA GAA GAA GAC CTG CAG GGG ATC TTC CAG TTA GAG GAA .A.
```

```
           841                                                                                                                                                                           960
#Haplotype_1 GAT GCT GAC GAA GCT AGC TTG GGA AGT TTC GAC TTA GAG GGA GAA CTG ATA GAG GAA GAT CTA CAG GAG AGC TTC GAC TTA GAG GGA GAA CAG GAA GAA GAA GAC CTG CAG GAG GGC TTC
#Haplotype_2 ... ... ... ... ... ... ... ... ... ... ... ... ... ... ... ... ... ... ... ... ... ... ... ... ... ... ... ... ... ... ... ... ... ... ... ... ... ... ... ...
#Haplotype_3 ... ... ... ... ... ... ... ... ... ... ... ... ... ... ... ... ... ... ... ... ... ... ... ... ... ... ... ... ... ... ... ... ... ... ... ... ... ... ... ...
#Haplotype_4 ... ... ... ... ... ... ... ... ... ... ... ... ... ... .G. ... ... ... ... ... ... ... ... ... ... ... ... ... ... ... ... ... ... ... ... ... ... ... ... ...
#Haplotype_5 ... ... ... ... ... ... ... ... ... ... ... ... ... ... ... ... ... ... ... ... ... ... ... ... ... ... ... ... ... ... ... ... ... ... ... ... ... ... ... ...
#Haplotype_6 ... ... ... ... ... ... ... ... ... ... ... ... ... ... ... ... ... ... ... ... ... ... ... ... ... ... ... ... ... ... ... ... ... ... ... ... ... ... ... ...
#Haplotype_7 ... ... ... ... ... ... ... ... ... ... ... ... ... ... ... ... ... ... ... ... ... ... ... ... ... ... ... ... ... ... ... ... ... ... ... ... ... ... ... ...
#Haplotype_8 ... ... ... ... ... ... ... ... ... ... ... ... ... ... ... ... ... ... ... ... ... ... ... ... ... ... ... ... ... ... ... ... ... ... ... ... ... ... ... ...
#P_cynomolgi C.A .G. ..A ... .AC CTG CA. ..G ..C ... ..G ... ... .A. ... .A. GG. ..A ... ..C ..G ... .G. .T. ... C.G ... ... .A. ... ... .G. ... ... ... ... ... .G. A.. ...
           961                                                                                                                                                                          1080
#Haplotype_1 AAA TCA GAG GAA GAG GCC AAC CAG GGA GGG CAA CTC CCC CGA GAG ATA CCA CCA CAC GGA GAG GAA GCC GTA GAA CCT CCC CTG AGG GGA AAC AAA CCG AGC ATG GAA TAC GTG GGC AAT
#Haplotype_2 ... ... ... ... ... ... ... ... ... ... ... ... ... ... ... ... ... ... ... ... ... ... ... ... ... ... ... ... ... ... ... ... ... ... ... ... ... ... ... ...
#Haplotype_3 ... ... ... ... ... ... ... ... ... ... ... ... ... ... ... ... ... ... ... ... ... ... ... ... ... ... ... ... ... ... ... ... ... ... ... ... ... ... ... ...
#Haplotype_4 ... ... ... ... ... ... ... ... ... ... ... ... ... ... ... ... ... ... ... ... ... ... ... ... ... ... ... ... ... ... ... ... ... ... ... ... ... ... ... ...
#Haplotype_5 ... ... ... ... ... ... ... ... ... ... ... ... ... ... ... ... ... ... ... ... ... ... ... ... ... ... ... ... ... ... ... ... ... ... ... ... ... ... ... ...
#Haplotype_6 ... ... ... ... ... ... ... ... ... ... ... ... ... ... ... ... ... ... ... ... ... ... ... ... ... ... ... ... ... ... ... ... ... ... ... ... ... ... ... ...
#Haplotype_7 ... ... ... ... ... ... ... ... ... ... ... ... ... ... ... ... ... ... ... ... ... ... ... ... ... ... ... ... ... ... ... ... ... ... ... ... ... ... ... ...
#Haplotype_8 ... ... ... ... ... ... ... ... ... ... ... ... ... ... ... ... ... ... ... ... ... ... ... ... ... ... ... ... ... ... ... ... ... ... ... ... ... ... ... ...
#P_cynomolgi G.G .T. ... ... ..A ..T ..G ... ... .AA ... ..T .G. .A. ... .C. T.. T.. G.. ... ..C ... ... A.. ... ... ... ... ..T ... CT. ... ... .AT .C. ... ... C.. AA. G.A
          1081                                                                                                                                                                          1200
#Haplotype_1 CTG CAC AGC GAC GTA GGC CCA ACT GAA GGC AGT GCC AAC CAG ATA AGC CCC CCC TCA GTT GAC GAA AAA GGG AAG GAA GAT GGC GAC AAA TAT AAA TCT GCA TCT CAG GAT GGG GGC AAC
#Haplotype_2 ... ... ... ... ... ... ... ... ... ... ... ... ... ... ... ... ... ... ... ... ... ... ... ... ... ... ... ... ... ... ... ... ... ... ... ... ... ... ... ...
#Haplotype_3 ... ... ... ... ... ... ... ... ... ... ... ... ... ... ... ... ... ... ... ... ... ... ... ... ... ... ... ... ... ... ... ... ... ... ... ... ... ... ... ...
#Haplotype_4 ... ... ... ... ... ... ... ... ... ... ... ... ... ... ... ... ... ... ... ... ... ... ... ... ... ... ... ... ... ... ... ... ... ... ... ... ... ... ... ...
#Haplotype_5 ... ... ... ... ... ... ... ... ... ... ... ... ... ... ... ... ... ... ... ... ... ... ... ... ... ... ... ... ... ... ... ... ... ... ... ... ... ... ... ...
#Haplotype_6 ... ... ... ... ... ... ... ... ... ... ... ... ... ... ... ... ... ... ... ... ... ... ... ... ... ... ... ... ... ... ... ... ... ... ... ... ... ... ... ...
#Haplotype_7 ... ... ... ... ... ... ... ... ... ... ... ... ... ... ... ... ... ... ... ... ... ... ... ... ... ... ... ... ... ... ... ... ... ... ... ... ... ... ... ...
#Haplotype_8 ... ... ... ... ... ... ... ... ... ... ... ... ... ... ... ... ... ... ... ... ... ... ... ... ... ... ... ... ... ... ... ... ... ... ... ... ... ... ... ...
#P_cynomolgi ... ..G .A. ... ... .A. TT. ... ... .A. ... ... ..G ... ... .A. .TT ... CA. A.. ... ..C ... ..T ... ... ... .AA C.. G.. ... ... .T. .AG A.. ... A.. ... .A. TT.
          1201                                                                                                                                                                          1320
#Haplotype_1 TCA GTG GGG ATA AAT AAC TTC GGA GGC TGC TTC CAG GGG GGC AAT TCA AAT GGC ATC TGT CCT CTG GAT ATT TTT AAA AAG GTG CTA GAA GAC GAA AAT TTC TTG CAA GAG TTT GAC AGC
#Haplotype_2 ... ... ... ... ... ... ... ... ... ... ... ... ... ... ... ... ... ... ... ... ... ... ... ... ... ... ... ... ... ... ... ... ... ... ... ... ... ... ... ...
#Haplotype_3 ... ... ... ... ... ... ... ... ... ... ... ... ... ... ... ... ... ... ... ... ... ... ... ... ... ... ... ... ... ... ... ... ... ... ... ... ... ... ... ...
#Haplotype_4 ... ... ... ... ... ... ... ... ... ... ... ... ... ... ... ... ... ... ... ... ... ... ... ... ... ... ... ... ... ... ... ... ... ... ... ... ... ... ... ...
#Haplotype_5 ... ... ... ... ... ... ... ... ... ... ... ... ... ... ... ... ... ... ... ... ... ... ... ... ... ... ... ... ... ... ... ... ... ... ... ... ... ... ... ...
#Haplotype_6 ... ... ... ... ... ... ... ... ... ... ... ... ... ... ... ... ... ... ... ... ... ... ... T.. ... ... ... ... ... ... ... ... ... ... ... ... ... ... ... ...
#Haplotype_7 ... ... ... ... ... ... ... ... ... ... ... ... ... ... ... ... ... ... ... ... ... ... ... ... ... ... ... ... ... ... ... ... ... ... ... ... ... ... ... ...
#Haplotype_8 ... ... ... ... ... ... ... ... ... ... ... ... ... ... ... ... ... ... ... ... ... ... ... T.. ... ... ... ... ... ... ... ... ... ... ... ... ... ... ... ...
#P_cynomolgi ..C ... ... .C. ... ..T ... ... ... ... ... ... ... ..T ... ... ... ... GC. ... ..A ..A ... ... ... ... ... ... ... ... ..A ... ... ... ..T ... ... ... .CT ...
          1321                                                                                                                                                                          1440
#Haplotype_1 TTT ATT CAT AAC CTG TAT GGA TCC TCT AAG AAC AAC ACC CCC TGG GGA GGA GAC AAA ATG GGA AAT GAA AAC CTC TAC ATG GAT CTT TTC ACC AAC GCG CTA AGT TTC CTC AAC ACG ATT
#Haplotype_2 ... ... ... ... ... ... ... ... ... ... ... ... ... ... ... ... ... ... ... ... ... ... ... ... ... ... ... ... ... ... ... ... ... ... ... ... ... ... ... ...
#Haplotype_3 ... ... ... ... ... ... ... ... ... ... ... ... ... ... ... ... ... ... ... ... ... ... ... ... ... ... ... ... ... ... ... ... ... ... ... ... ... ... ... ...
#Haplotype_4 ... ... ... ... ... ... ... ... ... ... ... ... ... ... ... ... ... ... ... ... ... ... ... ... ... ... ... ... ... ... ... ... ... ... ... ... ... ... ... ...
#Haplotype_5 ... ... ... ... ... ... ... ... ... ... ... ... ... ... ... ... ... ... ... ... ... ... ... ... ... ... ... ... ... ... ... ... ... ... ... ... ... ... ... ...
#Haplotype_6 ... ... ... ... ... ... ... ... ... ... ... ... ... ... ... ... ... ... ... ... ... ... ... ... ... ... ... ... ... ... ... ... ... ... ... ... ... ... ... ...
#Haplotype_7 ... ... ... ... ... ... ... ... ... ... ... ... ... ... ... ... ... ... ... ... ... ... ... ... ... ... ... ... ... ... ... ... ... ... ... ... ... ... ... ...
#Haplotype_8 ... ... ... ... ... ... ... ... ... ... ... ... ... ... ... ... ... ... ... ... ... ... ... ... ... ... ... ... ... ... ... ... ... ... ... ... ... ... ... ...
#P_cynomolgi ... ..C G.. ... ... ... ... .G. ..G ... ..G G.. ... ... ..T ... A.. ... C.. ... ..G ... ..G ... ... ... ... ... ..C ... ..A ... ... ... ... ... ... ... ... ...
          1441       1449
#Haplotype_1 GAA GTG ATT
#Haplotype_2 ... ... ...
#Haplotype_3 ... ... ...
#Haplotype_4 ... ... ...
#Haplotype_5 --- --- ---
#Haplotype_6 --- --- ---
#Haplotype_7 --- --- ---
#Haplotype_8 --- --- ---
#P_cynomolgi ... ... .G.
```

The alignment shows the 8 haplotypes found in *pvmsp-7F* together with *pcmsp-7F* haplotype. Haplotype 1, Sal-I; Haplotype 2, Brazil-I and North Korea; Haplotype 3, India-VII; Haplotype 4, Mauritania-I; Haplotypes 5-8, Colombian isolates. Dots represent nucleotide identity. Codons under positive selection are shown in green (intra-species) and turquoise (inter-species) and those under negative selection are shown in fuchsia (inter-species).
