# Supplementary material for: Heterogeneous genetic diversity pattern in Plasmodium vivax genes encoding merozoite surface proteins (MSP) -7E, −7F and -7L
Source: Malar J. 2014 Dec 13;13:495. doi: 10.1186/1475-2875-13-495 (PMC4300842; doi:10.1186/1475-2875-13-495)
Supplement: Supplementary file 10 — Additional file 10: pvmsp-7L gene alignment. The alignment shows the 7 haplotypes found in pvmsp-7L together with pcmsp-7L haplotype. Haplotype 1, Sal-I; haplotype 2, Brazil-I, India-VII and North Korea; haplotype 3, Mauritania-I; haplotypes 4–7, Colombian isolates. The dots represent nucleotide identity. Codons under positive selection are shown in green (intra-species) and in turquoise (inter-species) and those under negative selection are shown in fuchsia (inter-species). (PDF 91 KB) [file 12936_2014_3635_MOESM10_ESM.pdf]

Additional file 10 *pvmsp-7L* gene alignment

```
              1                                                                                                                                                        120
#Haplotype_1 ATG AAA ACA AAA GTA CTA TTC TTC TTG CCG TCC ATT TTG CTG CTA CCA CAT TCG GTA TGG TCA GAA ACG AAA GGT CCA TCT GGG CCC CCC CCA AAT AAG AAG CTA AAT GCT AAC GCA CTA
#Haplotype_2 ... ... ... ... ... ... ... ... ... ... C.. ... ... ... ... ... ... ... ... ... ... ... ... ... ... ... ... ... ... ... ... ... ... ... ... ... ... ... ... ...
#Haplotype_3 ... ... ... ... ... ... ... ... ... ... C.. ... ... ... ... ... ... ... ... ... ... ... ... ... ..A ... ... ... ... ... ... ... ... ... ... ... ... ... ... ...
#Haplotype_4 --- --- --- --- --- --- --- --- --- --- --- ... ... ... ... ... ... ... ... ... ... ... ... ... ... ... ... ... ... ... ... ... ... ... ... ... ... ... ... ...
#Haplotype_5 --- --- --- --- --- --- --- --- --- --- --- ... ... ... ... ... ... ... ... ... ... ... ... ... ... ... ... ... ... ... ... ... ... ... ... ... ... ... ... ...
#Haplotype_6 --- --- --- --- --- --- --- --- --- --- --- ... ... ... ... ... ... ... ... ... ... ... ... ... ... ... ... ... ... ... ... ... ... ... ... ... ... ... ... ...
#Haplotype_7 --- --- --- --- --- --- --- --- --- --- --- ... ... ... ... ... ... ... ... ... ... ... ... ... ... ... ... ... ... ... ... ... ... ... ... ... ... ... ... ...
#P_cynomolgi ... ... .T. ... ... ... ... A.. ... ..A C.. ... ... T.. ... ... ... ... ... ... ... ... ... ... ... ... C.. ... ..T ... AA. .C. C.. .T. .C. ... ... ... A.. ...
            121                                                                                                                                                        240
#Haplotype_1 CAT TTC TTA AGG GGC AAA TTA GAG TTA CTA AAT AAA ATC AGC GAG GAG CAG GTA GTT TCT CCA GAT TTT AAA AAA AAC GTA GAA CTG CTG AAA AAG AAA ATT GAG GAA CTA CAG GGT AAG
#Haplotype_2 ... ... ... ... ... ... ... ... ... ... ... ... ... ... ... ... ... ... ... ... ... ... ... ... ... ... ... ... ... ... ... ... ... ... ... ... ... ... ... ...
#Haplotype_3 ... ... ... ... ... ... ... ... ... ... ... ... ... ... ... ... ... ... ... ... ... ... ... ... ... ... ... ... ... ... ... ... ... ... ... ... ... ... ... ...
#Haplotype_4 ... ... ... ... ... ... ... ... ... ... ... ... ... ... ... ... ... ... ... ... ... ... ... ... ... ... ... ... ... ... ... ... ... ... ... ... ... ... ... ...
#Haplotype_5 ... ... ... ... ... ... ... ... ... ... ... ... ... ... ... ... ... ... ... ... ... ... ... ... ... ... ... ... ... ... ... ... ... ... ... ... ... ... ... ...
#Haplotype_6 ... ... ... ... ... ... ... ... ... ... ... ... ... ... ... ... ... ... ... ... ... ... ... ... ... ... ... ... ... ... ... ... ... ... ... ... ... ... ... ...
#Haplotype_7 ... ... ... ... ... ... ... ... ... ... ... ... ... ... ... ... ... ... ... ... ... ... ... ... ... ... ... ... ... ... ... ... ... ... ... ... ... ... ... ...
#P_cynomolgi ... ..T C.. .AA ... ... ... ..A CAC ... ... ... ... ... A.A ... A.T .C. ..A ... ... ... ..A ... ... ... A.. ... ... ..A ... ..A ... ..C ... ... ... G.A ..G ...
            241                                                                                                                                                        360
#Haplotype_1 GCA GAA AAG GAT AAA AGT AAA ACG GAT GGG GAG GAT ACC ACA CCC AAG GAA CAG CAA GAA GAT CAA AAT GTG AGT CAA AAC GGA TTG GAA GAG CAG GCC CCA AGT GAC AGT AAC GAG GGA
#Haplotype_2 ... ... ... ... ... ... ... ... ... ... ... ... ... ... ... ... ... ... ... ... ... ... ... ... ... ... ... ... ... ... ... ... ... ... ... ... ... ... ... ...
#Haplotype_3 ... ... ... ... ... ... ... ... ... ... ... ... ... ... ... ... ... ... ... ... ... ... ... ... ... ... ... ... ... ... ... ... ... ... ... ... ... ... ... ...
#Haplotype_4 ... ... ... ... ... ... ... ... ... ... ... ... ... ... ... ... ... ... ... ... ... ... ... ... ... ... ... ... ... ... ... ... ... ... ... ... ... ... ... ...
#Haplotype_5 ... ... ... ... ... ... ... ... ... ... ... ... ... ... ... ... ... ... ... ... ... ... ... ... ... ... ... ... ... ... ... ... ... ... ... ... ... ... ... ...
#Haplotype_6 ... ... ... ... ... ... ... ... ... ... ... ... ... ... ... ... ... ... ... ... ... ... ... ... ... ... ... ... ... ... ... ... ... ... ... ... ... ... ... ...
#Haplotype_7 ... ... ... ... ... ... ... ... ... ... ... ... ... ... ... ... ... ... ... ... ... ... ... ... ... ... ... ... ... ... ... ... ... ... ... ... ... ... ... ...
#P_cynomolgi ... ... ..A ... ... --- --- CA. ... ... .T. ... ..T .T. ... .GT .G. ... ... .C. TG. G.. ... ... ... ... ... ... ..A ... ... A.. A.A ... T.. .G. ..C ..T ... ...
            361                                                                                                                                                        480
#Haplotype_1 GAA GCG CAG GAA GAA AAC ACT CAA GTC AAA AAT GTC ATT TTT ACG GAG AAG GAA GAA GCG GTA GAT GAA GAA GCT GAA AAA GAA GAC ACC GCT GTC ATA AGT GAA AAG GCG AAT TTT CCA
#Haplotype_2 ... ... ... ... ... ... ... ... ... ... ... ... ... ... ... ... ... ... ... ... ... ... ... ... ... ... ... ... ... ... ... ... ... ... ... ... ... ... ..G ...
#Haplotype_3 ... ... ... ... ... ... ... ... ... ... ... ... ... ... ... ... ... ... ... ... ... ... ... ... ... ... ... ... ... ... ... ... ... ... ... ... ... ... ..G ...
#Haplotype_4 ... ... ... ... ... ... ... ... ... ... ... ... ... ... ... ... ... ... ... ... ... ... ... ... ... ... ... ... ... ... ... ... ... ... ... ... ... ... ..G ...
#Haplotype_5 ... ... ... ... ... ... ... ... ... ... ... ... ... ... ... ... ... ... ... ... ... ... ... ... ... ... ... ... ... ... ... ... ... ... ... ... ... ... ..G ...
#Haplotype_6 ... ... ... ... ... ... ... ... ... ... ... ... ... ... ... ... ... ... ... ... ... ... ... ... ... ... ... ... ... ... ... ... ... ... ... ... ... ... ... ...
#Haplotype_7 ... ... ... ... ... ... ... ... ... ... ... ... ... ... ... ... ... ... ... ... ... ... ... ... ... ... ... ... ... ... ... ... ... ... ... ... ... ... ..G ...
#P_cynomolgi A.. ... G.. ... A.G ..A ... ... ... ... ... ... ... ... .T. A.A ... ..G A.. ... A.C ... ... ... .T. .GG ..C A.. ... ... ... A.. ... .C. ... ... AA. ..A ..G ...
            481                                                                                                                                                        600
#Haplotype_1 AAT GAG GAA TCG CAA GGA AAT GAT GAA ACG CAA ACG CAG GAG AGC ATC GAG GGA GAA GCT TCT CCC GGA GTT GTA GTC GAC GAG ACA GAT GAC TCA CCA GAA GGC GAA CCC CTA TCC GGA
#Haplotype_2 ... ... ... ... ... ... ... ... ... ... ... ... ... ... ... ... ... ... ... ... ... ... ... ... ... ... ... ... ... ... ... ... ... ... ... ... ... ... ... ...
#Haplotype_3 ... ... ... ... ... ... ... ... ... ... ... ... ... ... ... ... ... ... ... ... ... ... ... ... ... ... ... ... ... ... ... ... ... ... ... ... ... ... ... ...
#Haplotype_4 ... ... ... ... ... ... ... ... ... ... ... ... ... ... ... ... ... ... ... ... ... ... ... ... ... ... ... ... ... ... ... ... ... ... ... ... ... ... ... ...
#Haplotype_5 ... ... ... ... ... ... ... ... ... ... ... ... ... ... ... ... ... ... ... ... ... ... ... ... ... ... ... ... ... ... ... ... ... ... ... ... ... ... ... ...
#Haplotype_6 ... ... ... ... ... ... ... ... ... ... ... ... ... ... ... ... ... ... ... ... ... ... ... ... ... ... ... ... ... ... ... ... ... ... ... ... ... ... ... ...
#Haplotype_7 ... ... ... ... ... ... ... ... ... ... ... ... ... ... ... ... ... ... ... ... ... ... ... ... ... ... ... ... ... ... ... ... ... ... ... ... ... ... ... ...
#P_cynomolgi .T. ... ... ... .CG ... .TG ... .G. ... ..G G.. A.. ... ..T ... ... ... ... ..G .TA ... ... ... A.. ..G ... A.. ... .G. ..T ... ... A.G ..A ... AT. ... ... ...
            601                                                                                                                                                        720
#Haplotype_1 TTG GAA ACG GAA GGC AAT TCT TCA GCT GAG AGT GCC CCA AAT GAG CCG GAC GTT AAC ACC ACA CAT ACG GCA GTA GAT ACG CAC ATG CCA GCA GAT GCG AAC ATA GGA GTA --- --- ---
#Haplotype_2 ... ... ... ... ... ... ... ... ... ... ... ... ... ... ... ... ... ... ... ... ... ... ... ... ... ... ... ... ... ... ... ... ... ... ... ... ... --- --- ---
#Haplotype_3 ... ... ... ... ... ... ... ... ... ... ... ... ... ... ... ... ... ... ... ... ... ... ... ... ... ... ... ... ... ... ... ... ... ... ... ... ... --- --- ---
#Haplotype_4 ... ... ... ... ... ... ... ... ... ... ... ... ... ... ... ... ... ... ... ... ... ... ... ... ... ... ... ... ... ... ... ... ... ... ... ... ... --- --- ---
#Haplotype_5 ... ... ... ... ... ... ... ... ... ... ... ... ... ... ... ... ... ... ... ... ... ... ... ... ... ... ... ... ... ... ... ... ... ... ... ... ... --- --- ---
#Haplotype_6 ... ... ... ... ... ... ... ... ... ... ... ... ... ... ... ... ... ... ... ... ... ... ... ... ... ... ... ... ... ... ... ... ... ... ... ... ... --- --- ---
#Haplotype_7 ... ... ... ... ... ... ... ... ... ... ... ... ... ... ... ... ... ... ... ... ... ... ... ... ... ... ... ... ... ... ... ... ... ... ... ... ... --- --- ---
#P_cynomolgi .C. ... ... ... ..T ..G C.. ... ... ..A ..C A.T ... ... ..A ... A.T ... ... .T. .T. ... --- --- --- --- --- --- ..A ... ... C.C A.A G.. TC. ..C AC. CAC ACA CAC
            721                                                                                                                                                        840
#Haplotype_1 --- GAC ACA AAT ATG CCA TTT GAC ACA CCT CCC CAC CCA AGC GGC GAA AAC CCC GGC GCA CCC CAA GAG ACG CAC CTT CCC TCC ATT GAC GAA AAT GCA AAT AGA AGA GCA TCA CGA ATG
#Haplotype_2 --- ... ... ... ... ... ... ... ... ... ... ... ... ... ... ... ... ... ... ... ... ... ... ... ... ... ... ... ... ... ... ... ... ... ... ... ... ... ... ...
#Haplotype_3 --- ... ... ... ... ... ... ... ... ... ... ... ... ... ... ... ... ... ... ... ... ... ... ... ... ... ... ... ... ... ... ... ... ... ... ... ... ... ... ...
#Haplotype_4 --- ... ... ... ... ... ... ... ... ... ... ... ... ... ... ... ... ... ... A.. ... ... ... ... ... ... ... ... ... ... ... ... ... ... ... ... ... ... ... ...
#Haplotype_5 --- ... ... ... ... ... ... ... ... ... ... ... ... ... ... ... ... ... ... ... ... ... ... ... ... ... ... ... ... ... ... ... ... ... ... ... ... ... ... ...
#Haplotype_6 --- ... ... ... ... ... ... ... ... ... ... ... ... ... ... ... ... ... ... ... ... ... ... ... ... ... ... ... ... ... ... ... ... ... ... ... ... ... ... ...
#Haplotype_7 --- ... ... ... ... ... ... ... ... ... ... ... ... ... ... ... ... ... ... ... ... ... ... ... ... ... ... ... ... ... ... ... ... ... ... ... ... ... ... ...
#P_cynomolgi TCA ..T ... ..C .CA GAC AAA C.. ... GAC A.A ... ... ... ... .G. .GT ... A.. ... ... ... A.. ..A .C. T.. T.. A.. ... T.. A.. ... ..T ..C ... ... ..G ... A.. .CT
```

```
             841                                                                                                                                                                      960
#Haplotype_1 AAA CAT ATG AGC AGC TTC CTA AAT GGC CTC TTA ACC AAT CAA AGC AAT AAC AAA AAG GAA ATA --- TTT TTC CAC CCA TAT TAT GGC CCC TAT TTT AAC CAC GGC GGG TAC TAT AAC TAT
#Haplotype_2 ... ... ... ... ... ... ... ... ... ... ... ... ... ... ... ... ... ... ... ... ... --- ... ... ... ... ... ... ... ... ... ... ... ... ... ... ... ... ... ...
#Haplotype_3 ... ... ... ... ... ... ... ... ... ... ... ... ... ... ... ... ... ... ... ... ... --- ... ... ... ... ... ... ... ... ... ... ... ... ... ... ... ... ... ...
#Haplotype_4 ... ... ... ... ... ... ... ... ... ... ... ... ... ... ... ... ... ... ... ... ... --- ... ... ... ... ... ... ... ... ... ... ... ... ... ... ... ... ... ...
#Haplotype_5 ... ... ... ... ... ... ... ... ... ... ... ... ... ... ... ... ... ... ... ... ... --- ... ... ... ... ... ... ... ... ... ... ... ... ... ... ... ... ... ...
#Haplotype_6 ... ... ... ... ... ... ... ... ... ... ... ... ... ... ... ... ... ... ... ... ... --- ... ... ... ... ... ... ... ... ... ... ... ... ... ... ... ... ... ...
#Haplotype_7 ... ... ... ... ... ... ... ... ... ... ... ... ... ... ... ... ... ... ... ... ... --- ... ... ... ... ... ... ... ... ... ... ... ... ... ... ... ... ... ...
#P_cynomolgi ... ..C ... .A. C.. ... ... G.. ... ... ... ... ... ... ... ..A ..A ... --. ... ..G --T A.. .-. ..T ... ... ... ... ... ... ... ... ..T CA. .CA ... ... ... ...
             961                                                                                                                                                                     1080
#Haplotype_1 GAC CCC TAT TAT AAT TAT GCC CCA GCG TAC AAC CCA TTT GTG AGC CAA GCA AGG GAT TAC GAA GTG ATT AAA AAG TTG CTT GAT GCC TGC TTT AAC AAA GGG GAA GGA GCC GAT CCA AAT
#Haplotype_2 ... ... ... ... ... ... ... ... ... ... ... ... ... ... ... ... ... ... ... ... ... ... ... ... ... ... ... ... ... ... ... ... ... ... ... ... ... ... ... ...
#Haplotype_3 ... ... ... ... ... ... ... ... ... ... ... ... ... ... ... ... ... ... ... ... ... ... ... ... ... ... ... ... ... ... ... ... ... ... ... ... ... ... ... ...
#Haplotype_4 ... ... ... ... ... ... ... ... ... ... ... ... ... ... ... ... ... ... ... ... ... ... ... ... ... ... ... ... ... ... ... ... ... ... ... ... ... ... ... ...
#Haplotype_5 ... ... ... ... ... ... ... ... ... ... ... ... ... ... ... ... ... ... ... ... ... ... ... ... ... ... ... ... ... ... ... ... ... ... ... ... A.. ... ... ...
#Haplotype_6 ... ... ... ... ... ... ... ... ... ... ... ... ... ... ... ... ... ... ... ... ... ... ... ... ... ... ... ... ... ... ... ... ... ... ... ... ... ... ... ...
#Haplotype_7 ... ... ... ... ... ... ... ... ... ... ... ... ... ... ... ... ... ... ... ... ... ... ... ... ... ... ... ... ... ... ... ... ... ... ... ... ... ... ... ...
#P_cynomolgi T.. ... ... ... ... ... C.. ..G .T. ..T ... ... .A. ... .C. ... A.. .A. ... ... ... ... G.. ... ..A ... A.. ... ..T ... ... ... ... ... ... ... ... ... ... ...
            1081                                                                                                                                                                     1200
#Haplotype_1 GTG CCC TGC ATA ATT GAC ATA TTC AAA AAA GTG CTA GAC GAC GAA CGG TTT CGA AAC GAA CTA AAA ACT TTT ATG TAT GAC CTT TAC GAA TTT TTG AAA AAG AAT GAC GTC TTA AGT GAT
#Haplotype_2 ... ... ... ... ... ... ... ... ... ... ... ... ... ... ... ... ... ... ... ... ..T ... ... ... ... ... ... ... ... ... ... ... ... ... ... ... ... ... ... ...
#Haplotype_3 ... ... ... ... ... ... ... ... ... ... ... ... ... ... ... ... ... ... ... ... ..T ... ... ... ... ... ... ... ... ... ... ... ... ... ... ... ... ... ... ...
#Haplotype_4 ... ... ... ... ... ... ... ... ... ... ... ... ... ... ... ... ... ... ... ... ..T ... ... ... ... ... ... ... ... ... ... ... ... ... ... ... ... ... ... ...
#Haplotype_5 ... ... ... ... ... ... ... ... ... ... ... ... ... ... ... ... ... ... ... ... ..T ... ... ... ... ... ... ... ... ... ... ... ... ... ... ... ... ... ... ...
#Haplotype_6 ... ... ... ... ... ... ... ... ... ... ... ... ... ... ... ... ... ... ... ... ..T ... ... ... ... ... ... ... ... ... ... ... ... ... ... ... ... ... ... ...
#Haplotype_7 ... ... ... ... ... ... ... ... ... ... ... ... ... ... ... ... ... ... ... ... ... ... ... ... ... ... ... ... ... ... ... ... ... ... ... ... ... ... ... ...
#P_cynomolgi ..A ... ... ... ... ... T.. ... ... ... ... T.. ... ... ... G.. ... ... ... ... T.T ... ... ..C ... ... A.. ... ... ... ... GCC ... ... ... ... ... ... ... ...
            1201                                                                                     1275
#Haplotype_1 GAT GAA AAG AAA AAC GAG TTG ATG AGA TTT TTC TTT GAC AAT GCC TTT CAG TTG GTC AAC CCG ATG TTT TAC TAC
#Haplotype_2 ... ... ... ... ... ... ... ... ... ... ... ... ... ... ... ... ... ... ... ... ... ... ... ... ...
#Haplotype_3 ... ... ... ... ... ... ... ... ... ... ... ... ... ... ... ... ... ... ... ... ... ... ... ... ...
#Haplotype_4 ... ... ... ... ... ... ... ... ... ... ... ... ... ... ... ... ... ... ... ... --- --- --- --- ---
#Haplotype_5 ... ... ... ... ... ... ... ... ... ... ... ... ... ... ... ... ... ... ... ... --- --- --- --- ---
#Haplotype_6 ... ... ... ... ... ... ... ... ... ... ... ... ... ... ... ... ... ... ... ... --- --- --- --- ---
#Haplotype_7 ... ... ... ... ... ... ... ... ... ... ... ... ... ... ... ... ... ... ... ... --- --- --- --- ---
#P_cynomolgi .GC .G. .G. ... ... ..A ... ... .A. ..C ... ... ... ... ... C.. .G. ... A.. ... A.. ... ..G C.. ..-
```

The alignment shows the 7 haplotypes found in *pvmsp-7L* together with *pcmsp-7L* haplotype. Haplotype 1, Sal-I; Haplotype 2, Brazil-I, India-VII and North Korea; Haplotype 3, Mauritania-I; Haplotypes 4-7, Colombian isolates. The dots represent nucleotide identity. Codons under positive selection are shown in green (intra-species) and in turquoise (inter-species) and those under negative selection are shown in fuchsia (inter-species).
