# Supplementary material for: Heterogeneous genetic diversity pattern in Plasmodium vivax genes encoding merozoite surface proteins (MSP) -7E, −7F and -7L
Source: Malar J. 2014 Dec 13;13:495. doi: 10.1186/1475-2875-13-495 (PMC4300842; doi:10.1186/1475-2875-13-495)
Supplement: Supplementary file 11 — Additional file 11: Inter-species positively and negatively selected sites detected for msp-7 genes. 5′-end (msp-7E: nucleotide 1–390, msp-7F: nucleotide 1–432, msp-7L: nucleotide 1–381), central (msp-7E: nucleotide 391–747, msp-7F: nucleotide 433–1,053, msp-7L: nucleotide 382–816) and 3′-end (msp-7E: nucleotide 748–1,158, msp-7F: nucleotide 1,054–1,449, msp-7L: nucleotide 817–1,275). Numbers based on Additional files 4, 9 and 10. (PDF 58 KB) [file 12936_2014_3635_MOESM11_ESM.pdf]

Heterogeneous genetic diversity pattern in *Plasmodium vivax* genes encoding merozoite surface proteins (MSP) -7E, -7F and -7L

Additional file 11 Inter-species positively and negatively selected sites detected for *pvmsp-7* genes.

Positively selected sites			
**Gene**	**5' end**	**central**	**3' end**
msp-7E	-	132, 133, 135, 136, 147, 151, 154, 161, 162, 164, 165, 169, 171, 172, 176, 178, 195, 200, 203, 211, 217, 239	319, 323, 371
msp-7F	6	212, 295	424
msp-7L	11, 49	159, 246, 247, 260	357
**Negatively selected sites**			
**Gene**	**5' end**	**central**	**3' end**
msp-7E	28, 39. 46, 48, 49, 52, 53, 56, 63, 64, 69, 72, 84, 88, 93, 96, 100, 104, 109, 110, 115, 118, 119, 127	156, 236, 245	252, 262, 263, 264, 272, 282, 283, 294, 300, 302, 303, 309, 311, 321, 327, 337, 338, 343, 349, 352, 356, 357, 358, 360, 361, 365, 366, 368, 377, 379
msp-7F	2, 7, 34, 38, 46, 104, 109	149, 168, 183, 195, 204, 207, 289, 298, 300, 325	384, 401, 406, 421, 449, 463, 471
msp-7L	25, 42, 48, 59, 72, 74, 79, 83, 117, 118	138, 175, 180, 186, 191, 195, 210, 211, 215, 236, 242, 244, 245	273, 274, 279, 280, 282, 305, 314, 330, 345, 381, 384, 406, 410

5'-end (*pvmsp-7E*: nucleotide 1–390, *pvmsp-7F*: nucleotide 1–432, *pvmsp-7L*: nucleotide 1–381), central (*pvmsp-7E*: nucleotide 391–747, *pvmsp-7F*: nucleotide 433–1,053, *pvmsp-7L*: nucleotide 382–816) and 3'-end (*pvmsp-7E*: nucleotide 748–1,158, *pvmsp-7F*: nucleotide 1,054–1,449, *pvmsp-7L*: nucleotide 817–1,275). Numbers based on Additional files 4, 9 and 10.
