# Supplementary material for: Heterogeneous genetic diversity pattern in Plasmodium vivax genes encoding merozoite surface proteins (MSP) -7E, −7F and -7L
Source: Malar J. 2014 Dec 13;13:495. doi: 10.1186/1475-2875-13-495 (PMC4300842; doi:10.1186/1475-2875-13-495)
Supplement: Supplementary file 12 — Additional file 12: pcmsp-7L putative donor and acceptor sites. An alignment was made between the Sal-I strain pvmsp-7L sequences, pcmsp-7L and the sequence resulting from GeneScan analysis (pcmsp-7L_mRNA). The red arrows indicate the putative donor and acceptor sites in pcmsp-7L. (PDF 62 KB) [file 12936_2014_3635_MOESM12_ESM.pdf]

Additional file 12 *pcmsp-7L* putative donor and acceptor sites.

```
                            *        20         *        40         *        60         *
Sal-I_pvmsp-7L : ATGAAAACAAAAGTACTATTCTTCTTGCCGTCCATTTTGCTGCTACCACATTCGGTATGGTCAGAAACGAAAGGTC :  76
Pcmsp-7L_DNA   : ATGAAAATAAAAGTACTATTCATCTTGCCACCCATTTTGTTGCTACCACATTCGGTATGGTCAGAAACGAAAGGTC :  76
Pcmsp-7L_mRNA  : ATGAAAATAAAAGTACTATTCATCTTGCCACCCATTTTGTTGCTACCACATTCGGTATGGTCAGAAACGAAAGGTC :  76

                   80         *       100         *       120         *       140         *
Sal-I_pvmsp-7L : CATCTGGGCCCCCCCCAAATAAGAAGCTAAATGCTAACGCACTACATTTCTTAAGGGGCAAATTAGAGTTACTAAA :  152
Pcmsp-7L_DNA   : CACCTGGGCCTCCCAAAACTCAGATGCCAAATGCTAACACACTACATTTTCTAAAAGGCAAATTAGAACACCTAAA :  152
Pcmsp-7L_mRNA  : CACCTGGGCCTCCCAAAACTCAGATGCCAAATGCTAACACACTACATTTTCTAAAAGGCAAATTAGAACACCTAAA :  152

                      160         *       180         *       200         *       220
Sal-I_pvmsp-7L : TAAAATCAGCGAGGAGCAGGTAGTTTCTCCAGATTTTAAAAAAAACGTAGAACTGCTGAAAAAGAAAATTGAGGAA :  228
Pcmsp-7L_DNA   : TAAAATCAGCAAAGAGAATGCAGTATCTCCAGATTTAAAAAAAAACATAGAACTGCTAAAAAAAAAAATCGAGGAA :  228
Pcmsp-7L_mRNA  : TAAAATCAGCAAAGAGAATGCAGTATCTCCAGATTTAAAAAAAAACATAGAACTGCTAAAAAAAAAAATCGAGGAA :  228

                  *       240         *       260         *       280         *       300
Sal-I_pvmsp-7L : CTACAGGGTAAGGCAGAAAAGGATAAAAGTAAAACGGATGGGGAGGATACCACACCCAAGGAACAGCAAGAAGATC :  304
Pcmsp-7L_DNA   : CTAGAAGGGAAGGCAGAAAAAGATAAACA------GGATGGGGTGGATACTATACCCAGTGGACAGCAAGCATGTG :  298
Pcmsp-7L_mRNA  : CTAGAAGGGAAGGCAGAAAAAGATAAACA------GGATGGGGTGGATACTATACCCAGTGGACAGCAAGCATGTG :  298

                      *       320         *       340         *       360         *       380
Sal-I_pvmsp-7L : AAAATGTGAGTCAAAACGGATTGGAAGAGCAGGCCCCAAGTGACAGTAACGAGGGAGAAGCGCAGGAAGAAAACAC :  380
Pcmsp-7L_DNA   : AAAATGTGAGTCAAAACGGATTAGAAGAGAAGACACCATGTGGCAGCAATGAGGGAAAAGCGGAGGAAAAGAAAAC :  374
Pcmsp-7L_mRNA  : AAAATGTGAGTCAAAACGGATTAGAAGAGAAGACACCATGTGGCAGCAATGAGGGAAAAGCGGAGGAAAAGAAAAC :  374

                          *       400         *       420         *       440         *
Sal-I_pvmsp-7L : TCAAGTCAAAAATGTCATTTTTACGGAGAAGGAAGAAGCGGTAGATGAAGAAGCTGAAAAAGAAGACACCGCTGTC :  456
Pcmsp-7L_DNA   : TCAAGTCAAAAATGTCATTTTTATGAAAAAGGAGAAAGCGATCGATGAAGAAGTTGGGAACAAAGACACCGCTATC :  450
Pcmsp-7L_mRNA  : TCAAGTCAAAAATGTCATTTTTATGAAAAAGGAGAAAGCGATCGATGAAGAAGTTGGGAACAAAGACACCGCTATC :  450
```

```
                       460         *       480         *       500         *       520         *
Sal-I_pvmsp-7L : ATAAGTGAAAAGGCGAATTTTCCAAATGAGGAATCGCAAGGAAATGATGAAACGCAAACGCAGGAGAGCATCGAGG :  532
Pcmsp-7L_DNA   : ATAACTGAAAAGAAGAAATTGCCAATTGAGGAATCGCCGGGAATGGATGGAACGCAGGCGAAGGAGAGTATCGAGG :  526
Pcmsp-7L_mRNA  : ATAACTGAAAAGAAGAAATTGCCAATTGAGGAATCGCCGGGAATGGATGGAACGCAGGCGAAGGAGAGTATCGAGG :  526

                           540         *       560         *       580         *       600
Sal-I_pvmsp-7L : GAGAAGCTTCTCCCGGAGTTGTAGTCGACGAGACAGATGACTCACCAGAAGGCGAACCCCTATCCGGATTGGAAAC :  608
Pcmsp-7L_DNA   : GAGAAGCGTTACCCGGAGTTATAGTGGACAAGACAGGTGATTCACCAAAGGGAGAAATCCTATCCGGATCGGAAAC :  602
Pcmsp-7L_mRNA  : GAGAAGCGTTACCCGGAGTTATAGTGGACAAGACAGGTGATTCACCAAAGGGAGAAATCCTATCCGGATCGGAAAC :  602

                   *           620         *       640         *       660         *       680
Sal-I_pvmsp-7L : GGAAGGCAATTCTTCAGCTGAGAGTGCCCCAAATGAGCCGGACGTTAACACCACACATACGGCAGTAGATACGCAC :  684
Pcmsp-7L_DNA   : GGAAGGTAAGCCTTCAGCTGAAAGCACTCCAAATGAACCGAATGTTAACATCATACATATACCAGCACACACAGAC :  678
Pcmsp-7L_mRNA  : GGAAG----------------------------------------------------------------------- :  607

                       *       700         *       720         *       740         *       760
Sal-I_pvmsp-7L : ATGCCAGCAGATGCGAACATAGGAGTAGACACAAATATGCCATTTGACACACCTCCCCACCCAAGCGGCGAAAACC :  760
Pcmsp-7L_DNA   : TCAGGCACACACACACACACTCAGATACAAACACAGACAAAC------ACACAGACACACACCCAAGCGGCGGAAGTC :  748
Pcmsp-7L_mRNA  : ---------------------------------------------------------------------------- :    -

                           *       780         *       800         *       820         *
Sal-I_pvmsp-7L : CCGGCGCACCCCAAGAGACGCACCTTCCCTCCATTGACGAAAATGCAAATAGAAGAGCATCACGAATGAAACATAT :  836
Pcmsp-7L_DNA   : CCAGCGCACCCCAAAAGACACCCTTTTCCACCATTTACAAAAATGCTAACAGAAGAGCGTCAAGAACTAAACACAT :  824
Pcmsp-7L_mRNA  : ---------------------------------------------------------------------------- :    -

                       840         *       860         *       880         *       900         *
Sal-I_pvmsp-7L : GAGCAGCTTCCTAAATGGCCTCTTAACCAATCAAAGCAATAACAAAAAGGAAATATTTTTCCACCCATATTATGGC :  912
Pcmsp-7L_DNA   : GAACCGCTTCCTAGATGGCCTCTTAACCAATCAAAGCA--AAAAAAAAGGAAATGTATTTCCATCCATATTATGGC :  898
Pcmsp-7L_mRNA  : ---------------------------------------------------------------------------- :    -
```

```
                         920         *        940         *        960         *        980
Sal-I_pvmsp-7L : CCCTATTTTAACCACGGCGGGTACTATAACTATGACCCCTATTATAATTATGCCCCAGCGTACAACCCATTTGTGA :  988
Pcmsp-7L_DNA   : CCCTATTTTAACCATCACGCATACTATAACTATTACCCCTATTATAATTATCCCCCGGTGTATAACCCATATGTGA :  974
Pcmsp-7L_mRNA  : ---------------------------------------------------------------------------- :    -

                  *      1000         *       1020         *       1040         *       1060
Sal-I_pvmsp-7L : GCCAAGCAAGGGATTACGAAGTGATTAAAAAGTTGCTTGATGCCTGCTTTAACAAAGGGGAAGGAGCCGATCCAAA : 1064
Pcmsp-7L_DNA   : CCCAAACAAAGGATTACGAAGTGGTTAAAAAATTGATTGATGCTTGCTTTAACAAAGGGGAAGGAGCCGATCCAAA : 1050
Pcmsp-7L_mRNA  : ---------------------------------------------------------GGGAAGGAGCCGATCCAAA :  626

                      *      1080         *       1100         *       1120         *       1140
Sal-I_pvmsp-7L : TGTGCCCTGCATAATTGACATATTCAAAAAAGTGCTAGACGACGAACGGTTTCGAAACGAACTAAAAACTTTTATG : 1140
Pcmsp-7L_DNA   : TGTACCCTGCATAATTGACTTATTCAAAAAAGTGTTAGACGACGAAGGGTTTCGAAACGAATTTAAAACTTTCATG : 1126
Pcmsp-7L_mRNA  : TGTACCCTGCATAATTGACTTATTCAAAAAAGTGTTAGACGACGAAGGGTTTCGAAACGAATTTAAAACTTTCATG :  702

                          *      1160         *       1180         *       1200         *
Sal-I_pvmsp-7L : TATGACCTTTACGAATTTTTGAAAAAGAATGACGTCTTAAGTGATGATGAAAAGAAAAACGAGTTGATGAGATTTT : 1216
Pcmsp-7L_DNA   : TATAACCTTTACGAATTTGCCAAAAAGAATGACGTCTTAAGTGATGGCGGAAGGAAAAACGAATTGATGAAATTCT : 1202
Pcmsp-7L_mRNA  : TATAACCTTTACGAATTTGCCAAAAAGAATGACGTCTTAAGTGATGGCGGAAGGAAAAACGAATTGATGAAATTCT :  778

                 1220          *      1240         *       1260
Sal-I_pvmsp-7L : TCTTTGACAATGCCTTTCAGTTGGTCAACCCGATGTTTTACTACTGA : 1263
Pcmsp-7L_DNA   : TCTTTGACAATGCCCTTCGGTTGATCAACACGATGTTGCACTACTGA : 1249
Pcmsp-7L_mRNA  : TCTTTGACAATGCCCTTCGGTTGATCAACACGATGTTGCACTACTGA :  825
```

An alignment was made between the Sal-I strain *pvmsp-7L* sequences, *pcmsp-7L* and the sequence resulting from GeneScan analysis (*pcmsp-7L*_mRNA). The red arrows indicate the putative donor and acceptor sites in *pcmsp-7L*.
